# An *IL1RL1* genetic variant lowers soluble ST2 levels and the risk effects of *APOE*-ε4 in female patients with Alzheimer’s disease

**DOI:** 10.1038/s43587-022-00241-9

**Published:** 2022-07-15

**Authors:** Yuanbing Jiang, Xiaopu Zhou, Hiu Yi Wong, Li Ouyang, Fanny C. F. Ip, Vicky M. N. Chau, Shun-Fat Lau, Wei Wu, Daniel Y. K. Wong, Heukjin Seo, Wing-Yu Fu, Nicole C. H. Lai, Yuewen Chen, Yu Chen, Estella P. S. Tong, Alzheimer’s Disease Neuroimaging Initiative, Michael W. Weiner, Paul Aisen, Ronald Petersen, Clifford R. Jack, William Jagust, John Q. Trojanowski, Arthur W. Toga, Laurel Beckett, Robert C. Green, Andrew J. Saykin, John Morris, Leslie M. Shaw, Zaven Khachaturian, Greg Sorensen, Lew Kuller, Marcus Raichle, Steven Paul, Peter Davies, Howard Fillit, Franz Hefti, David Holtzman, Marek M. Mesulam, William Potter, Peter Snyder, Adam Schwartz, Tom Montine, Ronald G. Thomas, Michael Donohue, Sarah Walter, Devon Gessert, Tamie Sather, Gus Jiminez, Danielle Harvey, Matthew Bernstein, Paul Thompson, Norbert Schuff, Bret Borowski, Jeff Gunter, Matt Senjem, Prashanthi Vemuri, David Jones, Kejal Kantarci, Chad Ward, Robert A. Koeppe, Norm Foster, Eric M. Reiman, Kewei Chen, Chet Mathis, Susan Landau, Nigel J. Cairns, Erin Householder, Lisa Taylor-Reinwald, Virginia Lee, Magdalena Korecka, Michal Figurski, Karen Crawford, Scott Neu, Tatiana M. Foroud, Steven G. Potkin, Li Shen, Kelley Faber, Sungeun Kim, Kwangsik Nho, Leon Thal, Neil Buckholtz, Marylyn Albert, Richard Frank, John Hsiao, Jeffrey Kaye, Joseph Quinn, Betty Lind, Raina Carter, Sara Dolen, Lon S. Schneider, Sonia Pawluczyk, Mauricio Beccera, Liberty Teodoro, Bryan M. Spann, James Brewer, Helen Vanderswag, Adam Fleisher, Judith L. Heidebrink, Joanne L. Lord, Sara S. Mason, Colleen S. Albers, David Knopman, Kris Johnson, Rachelle S. Doody, Javier Villanueva-Meyer, Munir Chowdhury, Susan Rountree, Mimi Dang, Yaakov Stern, Lawrence S. Honig, Karen L. Bell, Beau Ances, Maria Carroll, Sue Leon, Mark A. Mintun, Stacy Schneider, Angela Oliver, Daniel Marson, Randall Griffith, David Clark, David Geldmacher, John Brockington, Erik Roberson, Hillel Grossman, Effie Mitsis, Leyla de Toledo-Morrell, Raj C. Shah, Ranjan Duara, Daniel Varon, Maria T. Greig, Peggy Roberts, Chiadi Onyike, Daniel D’Agostino, Stephanie Kielb, James E. Galvin, Brittany Cerbone, Christina A. Michel, Henry Rusinek, Mony J. de Leon, Lidia Glodzik, Susan De Santi, P. Murali Doraiswamy, Jeffrey R. Petrella, Terence Z. Wong, Steven E. Arnold, Jason H. Karlawish, David Wolk, Charles D. Smith, Greg Jicha, Peter Hardy, Partha Sinha, Elizabeth Oates, Gary Conrad, Oscar L. Lopez, MaryAnn Oakley, Donna M. Simpson, Anton P. Porsteinsson, Bonnie S. Goldstein, Kim Martin, Kelly M. Makino, M. Saleem Ismail, Connie Brand, Ruth A. Mulnard, Gaby Thai, Catherine McAdams-Ortiz, Kyle Womack, Dana Mathews, Mary Quiceno, Ramon Diaz-Arrastia, Richard King, Myron Weiner, Kristen Martin-Cook, Michael DeVous, Allan I. Levey, James J. Lah, Janet S. Cellar, Jeffrey M. Burns, Heather S. Anderson, Russell H. Swerdlow, Liana Apostolova, Kathleen Tingus, Ellen Woo, Daniel H. S. Silverman, Po H. Lu, George Bartzokis, Neill R. Graff-Radford, Francine Parfitt, Tracy Kendall, Heather Johnson, Martin R. Farlow, Ann Marie Hake, Brandy R. Matthews, Scott Herring, Cynthia Hunt, Christopher H. van Dyck, Richard E. Carson, Martha G. MacAvoy, Howard Chertkow, Howard Bergman, Chris Hosein, Ging-Yuek Robin Hsiung, Howard Feldman, Benita Mudge, Michele Assaly, Charles Bernick, Donna Munic, Andrew Kertesz, John Rogers, Dick Trost, Diana Kerwin, Kristine Lipowski, Chuang-Kuo Wu, Nancy Johnson, Carl Sadowsky, Walter Martinez, Teresa Villena, Raymond Scott Turner, Kathleen Johnson, Brigid Reynolds, Reisa A. Sperling, Keith A. Johnson, Gad Marshall, Meghan Frey, Barton Lane, Allyson Rosen, Jared Tinklenberg, Marwan N. Sabbagh, Christine M. Belden, Sandra A. Jacobson, Sherye A. Sirrel, Neil Kowall, Ronald Killiany, Andrew E. Budson, Alexander Norbash, Patricia Lynn Johnson, Joanne Allard, Alan Lerner, Paula Ogrocki, Leon Hudson, Evan Fletcher, Owen Carmichael, John Olichney, Charles DeCarli, Smita Kittur, Michael Borrie, T-Y. Lee, Rob Bartha, Sterling Johnson, Sanjay Asthana, Cynthia M. Carlsson, Adrian Preda, Dana Nguyen, Pierre Tariot, Stephanie Reeder, Vernice Bates, Horacio Capote, Michelle Rainka, Douglas W. Scharre, Maria Kataki, Anahita Adeli, Earl A. Zimmerman, Dzintra Celmins, Alice D. Brown, Godfrey D. Pearlson, Karen Blank, Karen Anderson, Robert B. Santulli, Tamar J. Kitzmiller, Eben S. Schwartz, Kaycee M. Sink, Jeff D. Williamson, Pradeep Garg, Franklin Watkins, Brian R. Ott, Henry Querfurth, Geoffrey Tremont, Stephen Salloway, Paul Malloy, Stephen Correia, Howard J. Rosen, Bruce L. Miller, Jacobo Mintzer, Kenneth Spicer, David Bachman, Stephen Pasternak, Irina Rachinsky, Dick Drost, Nunzio Pomara, Raymundo Hernando, Antero Sarrael, Susan K. Schultz, Laura L. Boles Ponto, Hyungsub Shim, Karen Elizabeth Smith, Norman Relkin, Gloria Chaing, Lisa Raudin, Amanda Smith, Kristin Fargher, Balebail Ashok Raj, Thomas Neylan, Jordan Grafman, Melissa Davis, Rosemary Morrison, Jacqueline Hayes, Shannon Finley, Karl Friedl, Debra Fleischman, Konstantinos Arfanakis, Olga James, Dino Massoglia, J. Jay Fruehling, Sandra Harding, Elaine R. Peskind, Eric C. Petrie, Gail Li, Jerome A. Yesavage, Joy L. Taylor, Ansgar J. Furst, Vincent C. T. Mok, Timothy C. Y. Kwok, Kin Y. Mok, Maryam Shoai, Benoit Lehallier, Patricia Morán Losada, Eleanor O’Brien, Tenielle Porter, Simon M. Laws, John Hardy, Tony Wyss-Coray, Colin L. Masters, Amy K. Y. Fu, Nancy Y. Ip

**Affiliations:** 1 Division of Life Science, State Key Laboratory of Molecular Neuroscience, Molecular Neuroscience Center, The Hong Kong University of Science and Technology, Clear Water Bay, Hong Kong, China; 2 Hong Kong Center for Neurodegenerative Diseases, Hong Kong Science Park, Hong Kong, China; 3 Guangdong Provincial Key Laboratory of Brain Science, Disease and Drug Development; Shenzhen–Hong Kong Institute of Brain Science, HKUST Shenzhen Research Institute, Shenzhen, China; 4 The Brain Cognition and Brain Disease Institute, Shenzhen Institutes of Advanced Technology, Chinese Academy of Sciences; Shenzhen–Hong Kong Institute of Brain Science–Shenzhen Fundamental Research Institutions, Shenzhen, China; 5 Gerald Choa Neuroscience Centre, Lui Che Woo Institute of Innovative Medicine, Therese Pei Fong Chow Research Centre for Prevention of Dementia, Division of Neurology, Department of Medicine and Therapeutics, The Chinese University of Hong Kong, Hong Kong, China; 6 Therese Pei Fong Chow Research Centre for Prevention of Dementia, Division of Geriatrics, Department of Medicine and Therapeutics, The Chinese University of Hong Kong, Hong Kong, China; 7 Department of Neurodegenerative Disease, UCL Queen Square Institute of Neurology, London, UK; 8 UK Dementia Research Institute, University College London, London, UK; 9 Department of Neurology and Neurological Sciences, Stanford University School of Medicine, Stanford, California USA; 10 Wu Tsai Neurosciences Institute, Stanford University, Stanford, California USA; 11 Centre for Precision Health, Edith Cowan University, Joondalup, Australia; 12 Collaborative Genomics and Translation Group, School of Medical and Health Sciences, Edith Cowan University, Joondalup, Australia; 13 School of Pharmacy and Biomedical Sciences, Faculty of Health Sciences, Curtin Health Innovation Research Institute, Curtin University, Bentley, Australia; 14 Institute for Advanced Study, The Hong Kong University of Science and Technology, Clear Water Bay, Hong Kong, China; 15 The Phil and Penny Knight Initiative for Brain Resilience, Stanford University, Stanford, California USA; 16 The Florey Institute of Neuroscience and Mental Health, The University of Melbourne, Melbourne, Australia; 17 UC San Francisco, San Francisco, California USA; 18 UC San Diego, San Diego, California USA; 19 Mayo Clinic, Rochester, New York USA; 20 UC Berkeley, Berkeley, California USA; 21 University of Pennsylvania, Philadelphia, Pennsylvania USA; 22 University of Southern California, Los Angeles, California USA; 23 UC Davis, Davis, California USA; 24 Brigham and Women’s Hospital/Harvard Medical School, Boston, Massachusetts, USA; 25 Indiana University, Bloomington, Indiana USA; 26 Washington University in St. Louis, St. Louis, Missouri USA; 27 Prevent Alzheimer’s Disease 2020, Rockville, Maryland USA; 28 Siemens, Munich, Germany; 29 University of Pittsburgh, Pittsburgh, Pennsylvania USA; 30 Weill Cornell Medical College, Cornell University, New York City, New York USA; 31 Albert Einstein College of Medicine, Yeshiva University, Bronx, New York USA; 32 Alzheimer’s Drug Discovery Foundation, New York City, New York USA; 33 Acumen Pharmaceuticals, Livermore, California USA; 34 Northwestern University, Evanston and Chicago, Evanston, Illinois USA; 35 National Institute of Mental Health, Rockville, Maryland USA; 36 Brown University, Providence, Rhode Island USA; 37 Eli Lilly, Indianapolis, Indiana, USA; 38 University of Washington, Seattle, Washington USA; 39 UCLA, Los Angeles, California USA; 40 University of Michigan, Ann Arbor, Michigan USA; 41 University of Utah, Salt Lake City, Utah USA; 42 Banner Alzheimer’s Institute, Phoenix, Arizona USA; 43 UC Irvine, Irvine, California, USA; 44 National Institute on Aging, Bethesda, Maryland USA; 45 Johns Hopkins University, Baltimore, Maryland USA; 46 Richard Frank Consulting, Washington, USA; 47 Oregon Health and Science University, Portland, Oregon USA; 48 Baylor College of Medicine, Houston, Texas USA; 49 University of Alabama, Birmingham, Alabama USA; 50 Mount Sinai School of Medicine, New York City, New York USA; 51 Rush University Medical Center, Chicago, Illinois USA; 52 Wien Center, Miami, Florida USA; 53 New York University, New York City, New York USA; 54 Duke University Medical Center, Durham, North Carolina USA; 55 University of Kentucky, Lexington, Kentucky USA; 56 University of Rochester Medical Center, Rochester, New York, USA; 57 University of Texas Southwestern Medical School, Dallas, Texas USA; 58 Emory University, Atlanta, Georgia USA; 59 Medical Center, University of Kansas, Kansas City, Kansas USA; 60 Mayo Clinic, Jacksonville, Florida USA; 61 Yale University School of Medicine, New Haven, Connecticut USA; 62 McGill University/Montreal-Jewish General Hospital, Montreal, Quebec Canada; 63 University of British Columbia Clinic for Alzheimer’s Disease and Related Disorders, Vancouver, British Columbia Canada; 64 Cleveland Clinic Lou Ruvo Center for Brain Health, Las Vegas, Nevada USA; 65 St Joseph’s Health Care, London, Ontario Canada; 66 Premiere Research Institute, Palm Beach Neurology, Miami, Florida USA; 67 Georgetown University Medical Center, Washington, D.C. USA; 68 Banner Sun Health Research Institute, Sun City, Arizona USA; 69 Boston University, Boston, Massachusetts USA; 70 Howard University, Washington, D.C. USA; 71 Case Western Reserve University, Cleveland, Ohio USA; 72 Neurological Care of CNY, Liverpool, New York USA; 73 Parkwood Hospital, London, Ontario Canada; 74 University of Wisconsin, Madison, Wisconsin USA; 75 Dent Neurologic Institute, Amherst, New York USA; 76 Ohio State University, Columbus, Ohio USA; 77 Albany Medical College, Albany, New York USA; 78 Olin Neuropsychiatry Research Center, Hartford Hospital, Hartford, Connecticut USA; 79 Dartmouth-Hitchcock Medical Center, Lebanon, New Hampshire USA; 80 Wake Forest University Health Sciences, Winston-Salem, North Carolina USA; 81 Rhode Island Hospital, Providence, Rhode Island USA; 82 Butler Hospital, Providence, Rhode Island USA; 83 Medical University South Carolina, Charleston, South Carolina USA; 84 Nathan Kline Institute, Orangeburg, New York USA; 85 University of Iowa College of Medicine, Iowa City, Iowa USA; 86 USF Health Byrd Alzheimer’s Institute, University of South Florida, Tampa, Florida USA; 87 Department of Defense, Arlington, Virginia USA; 88 Stanford University, Stanford, California USA; 89 Present Address: Alkahest Inc, San Carlos, California USA

**Keywords:** Alzheimer's disease, Neuroimmunology, Genetics of the nervous system, Ageing

## Abstract

Changes in the levels of circulating proteins are associated with Alzheimer’s disease (AD), whereas their pathogenic roles in AD are unclear. Here, we identified soluble ST2 (sST2), a decoy receptor of interleukin-33–ST2 signaling, as a new disease-causing factor in AD. Increased circulating sST2 level is associated with more severe pathological changes in female individuals with AD. Genome-wide association analysis and CRISPR–Cas9 genome editing identified rs1921622, a genetic variant in an enhancer element of *IL1RL1*, which downregulates gene and protein levels of sST2. Mendelian randomization analysis using genetic variants, including rs1921622, demonstrated that decreased sST2 levels lower AD risk and related endophenotypes in females carrying the Apolipoprotein E (*APOE*)-ε4 genotype; the association is stronger in Chinese than in European-descent populations. Human and mouse transcriptome and immunohistochemical studies showed that rs1921622/sST2 regulates amyloid-beta (Aβ) pathology through the modulation of microglial activation and Aβ clearance. These findings demonstrate how sST2 level is modulated by a genetic variation and plays a disease-causing role in females with AD.

## Abstract (web summary)

This study finds that sST2 is a disease-causing factor for Alzheimer’s disease. Higher sST2 levels impair microglial Aβ clearance in APOE4^+^ female individuals. A genetic variant, rs1921622, is associated with a reduction in sST2 level and protects against AD in APOE4^+^ female individuals.

## Main

AD is the most common neurodegenerative disease and a leading cause of mortality in older people^1^. Its pathological hallmarks include the extracellular accumulation of Aβ peptides, which form Aβ plaques, and intracellular neurofibrillary tangles composed of hyperphosphorylated tau protein (P-tau)^2^. While the pathophysiological mechanisms underlying AD remain unclear, genome-wide association studies (GWASs) reveal more than 40 AD-associated genes linked with microglial functions (for example*, APOE*, *TREM2*, *BIN1* and *CD33*), suggesting that microglia play a key role in AD pathogenesis^3–5^. In particular, *APOE*-ε4, the strongest known risk factor for sporadic AD after chronological age^3^, affects Aβ accumulation in AD^6,7^ through regulating the clustering of microglia around Aβ and the subsequent degradation of Aβ plaques^4,8,9^. This suggests that microglial dysfunction plays an essential causative role in AD.

Recent studies show that, besides genetic factors, changes in secreted proteins in the brain milieu and/or circulatory system may also disrupt microglial activities and contribute to AD pathogenesis^10,11^. For example, soluble TREM2 protein (sTREM2) level is increased in the cerebrospinal fluid (CSF) of individuals with AD^12–14^, and injection of sTREM2 in transgenic mouse models of amyloidosis alleviates Aβ accumulation by enhancing the interaction between microglia and Aβ and subsequent Aβ phagocytosis^15,16^. Moreover, the soluble form of a full-length VCAM1 protein in endothelial cells, sVCAM1, is increased in the plasma and CSF of individuals with AD^17,18^; this is suggested to mediate reduced hippocampal neurogenesis and a pro-inflammatory response by microglia during aging^19^.

**Extended Data Fig. 1:**
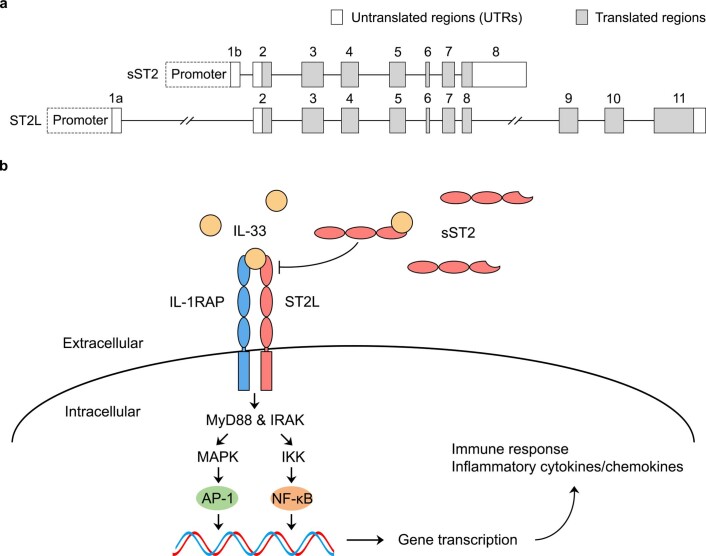
Diagrams illustrating IL-33–ST2 signaling. (a) Schematic showing the soluble ST2 (sST2) and full-length ST2 (ST2L) transcripts. (b) The IL-33/ST2 signaling pathways. IL-33, interleukin 33.

Other secreted proteins that contribute to AD pathogenesis include soluble cytokine receptors comprising the ectodomains of membrane-bound cytokine receptors, which function as decoy receptors to attenuate cytokine-mediated signaling^20,21^. In particular, sST2 is a secreted isoform of the interleukin-33 (IL-33) receptor ST2L (full-length ST2) that is produced by alternative promoter activation^22^ (Extended Data Fig. 1). ST2L is expressed by microglia in the brain^23^, and activation of IL-33–ST2 signaling decreases Aβ accumulation via enhanced microglial Aβ-clearance capacity in transgenic mouse models of amyloidosis^24,25^. Meanwhile, sST2 acts as a decoy receptor of IL-33 and inhibits IL-33–ST2 signaling^26,27^. Altered sST2 level in plasma is a biomarker of several inflammatory and cardiac diseases^28–31^. Notably, recent evidence shows that sST2 levels are also elevated in the blood of individuals with mild cognitive impairment (MCI) or AD^24,32^. Nonetheless, it is unclear what regulatory mechanisms underlie sST2 dysregulation and whether sST2 plays a pathological role in AD.

In the present study, we investigated sST2 regulation in AD and its roles in disease pathogenesis. We showed that sST2 level increases in the blood and brains of females with AD and is positively associated with disease progression. Moreover, we identified a single-nucleotide polymorphism (SNP), rs1921622, in *IL1RL1* (the gene encoding sST2 and ST2L) that is associated with decreased sST2 expression in human endothelial cells and decreased plasma and CSF sST2 levels. Mendelian randomization (MR) analysis showed that, in female *APOE*-ε4 carriers, decreased sST2 level results in reduced AD risk and less-severe AD-associated endophenotypes, suggesting a causal effect of sST2 in AD. Subsequent single-nucleus transcriptomic profiling revealed that the rs1921622 A allele is associated with enhanced microglial activation toward Aβ and lowered Aβ plaque load in female *APOE*-ε4 carriers with AD. Together, our findings suggest that a circulating protein, sST2, in the brain milieu plays a key role in *APOE*-ε4-dependent AD pathogenesis in females—by modulating the activation and Aβ-clearance capacity of microglia—and therefore might be a novel target for AD therapy.

## Results

### sST2 is associated with Alzheimer’s disease and its pathological changes

**Fig. 1:**
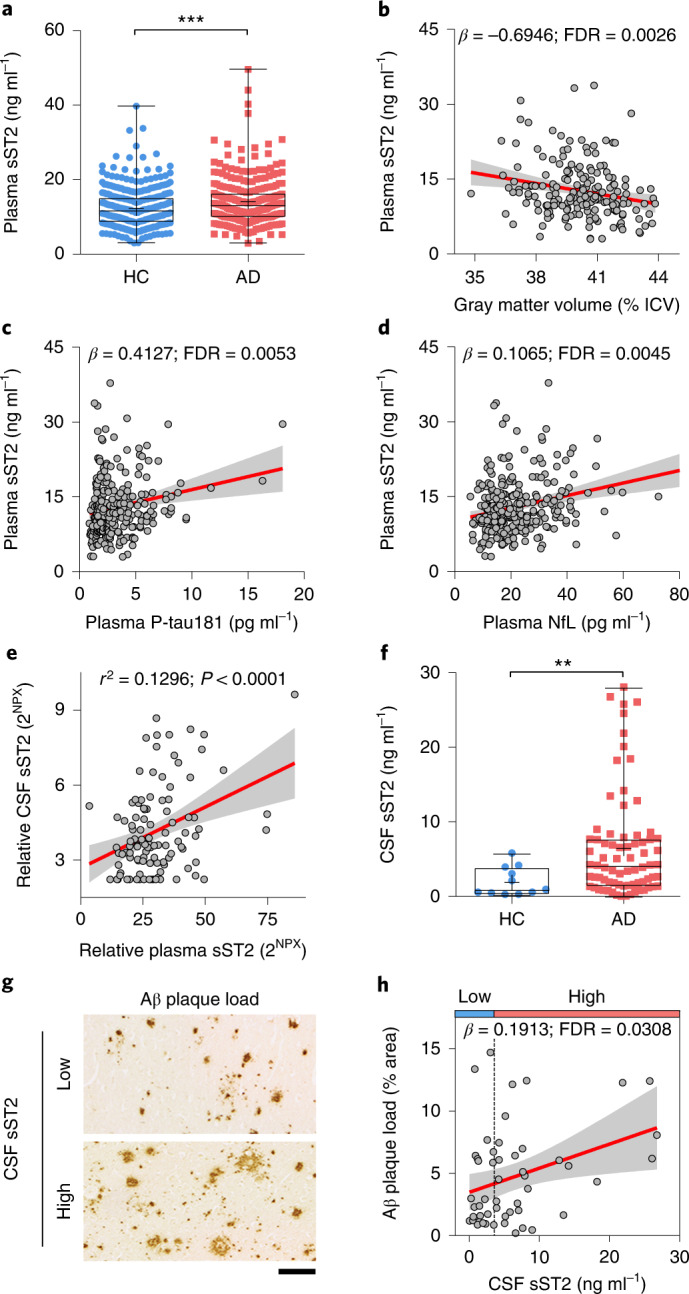
Soluble ST2 levels are associated with Alzheimer’s disease and its pathological changes. **a**, Individual plasma sST2 levels stratified by disease phenotype (*n* = 336 HCs, *n* = 277 individuals with AD; Chinese_cohort_1). *β* = 2.072. **b**–**d**, Associations between plasma sST2 level and AD-associated endophenotypes in Chinese_cohort_1. **b**, The intracranial volume (ICV)-normalized gray matter volume (*n* = 192). **c**, plasma P-tau181 levels (*n* = 290). **d**, plasma NfL levels (*n* = 289). **e**, Correlation between CSF and plasma levels of sST2 (*n* = 66 HCs, *n* = 23 individuals with MCI, and *n* = 18 individuals with AD; ADRC cohort). Linear regression test, adjusted for age, sex and disease diagnosis. *β* = 0.049; *r*^2^, Pearson’s correlation coefficient. 2^NPX^, linear form of normalized protein expression level. **f**, Individual CSF sST2 levels stratified by disease phenotype (*n* = 11 HCs, *n* = 75 individuals with AD; UKBBN cohort). *β* = 6.605. **g**,**h**, Associations between Aβ staining in the postmortem frontal cortex and CSF sST2 levels in individuals with AD (*n* = 51 individuals; UKBBN cohort). Individuals were stratified into two groups according to CSF sST2 levels: low, ≤3.6 ng ml^−1^; high, >3.6 ng ml^−1^. The vertical dashed line in **h** indicates the CSF sST2 level (3.6 ng ml^−1^) with the largest Youden’s index value for distinguishing HCs from individuals with AD. Representative images of Aβ staining in individuals with AD who had low and high CSF sST2 levels (**g**) and association analysis results (**h**). Scale bar, 100 μm. Data in box-and-whisker plots are presented with maximum, 75th percentile, median, 25th percentile and minimum values; plus signs denote mean values; data in regression lines are presented as the slope (red) and 95% confidence intervals (CIs; gray). Statistical tests for plasma sST2 were performed by linear regression analysis, adjusted for age, sex, CVD status, BMI and education level, with multiple testing correction. Statistical tests for CSF sST2 were performed by linear regression analysis, adjusted for age, sex and PMD, with multiple testing correction. *FDR < 0.05, **FDR < 0.01, ***FDR < 0.001.

**Extended Data Fig. 2:**
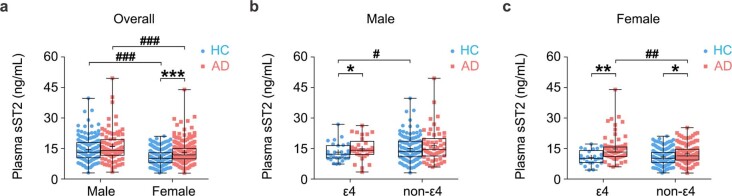
Associations between plasma soluble ST2 levels and Alzheimer’s disease in the Chinese_cohort_1, stratified by sex and *APOE*-ε4 genotypes. (a) Individual plasma soluble ST2 (sST2) levels stratified by sex and disease phenotype (male: *n* = 132 healthy controls [HCs], *n* = 84 individuals with Alzheimer’s disease [AD]; female: *n* = 204 HCs, *n* = 193 individuals with AD). Test for effects of sex: *β* = −3.848 and −3.257 in HCs and individuals with AD, respectively; test for effects of AD: *β* = 1.926 and 2.235 in males and females, respectively. (b, c) Individual plasma sST2 levels stratified by *APOE*-ε4 genotypes and disease phenotype in (b) males (male ε4: *n* = 28 HCs, *n* = 29 individuals with AD; male non-ε4: *n* = 104 HCs, *n* = 55 individuals with AD), and (c) females (female ε4: *n* = 25 HCs, *n* = 71 individuals with AD; female non-ε4: *n* = 179 HCs, *n* = 122 individuals with AD). Test for effects of *APOE*-ε4: *β* = −1.809, −1.499, −0.248, and 2.425 in male HCs, male individuals with AD, female HCs, and female individuals with AD, respectively; test for effects of AD: *β* = 2.034, 1.098, 3.833, and 1.811 in male ε4, male non-ε4, female ε4, and female non-ε4, respectively. ε4, *APOE*-ε4 carriers; non-ε4, *APOE*-ε4 noncarriers. Data are presented as box-and-whisker plots including maximum, 75^th^ percentile, median, 25^th^ percentile, and minimum values; plus signs (+) denote corresponding mean values. Linear regression test, adjusted for age, cardiovascular disease status, body mass index (BMI), and education level, with multiple testing correction; ^#^*FDR* < 0.05, ^##^*FDR* < 0.01, ^###^*FDR* < 0.001; *FDR < 0.05, **FDR < 0.01, ***FDR < 0.001. *FDR*, false discovery rate.

To investigate how sST2 is involved in AD pathogenesis, we examined the associations between sST2 level and AD and its related endophenotypes. We measured the plasma sST2 level of Chinese individuals with AD (that is, those having Alzheimer’s dementia with a Montreal Cognitive Assessment (MoCA) score < 21) and healthy controls (HCs) recruited in Hong Kong (the ‘Chinese_cohort_1’ hereafter; *n* = 345 HCs and *n* = 345 individuals with AD; Supplementary Table 1). We then performed a linear regression analysis between plasma sST2 level and AD and its related endophenotypes, adjusting for age, sex, status of cardiovascular diseases (CVDs; that is, heart disease, hypertension, diabetes mellitus and hyperlipidemia), body mass index (BMI) and education level, followed by multiple testing correction. The results show that plasma sST2 level was higher in individuals with AD when compared to HCs (*β* (effect size) = 2.072, false discovery rate (FDR) < 0.001; Fig. 1a). Moreover, plasma sST2 level was positively associated with the AD-related endophenotypes we examined—namely, the decreased volumes of gray matter (*β* = −0.695, FDR = 0.003; Fig. 1b) and increased levels of plasma biomarkers corresponding to AD (that is, P-tau181 (ref. ^33^), *β* = 0.413, FDR = 0.005; Fig. 1c) and neurodegeneration (that is, neurofilament light polypeptide (NfL)^34^, *β* = 0.107, FDR = 0.004; Fig. 1d). Notably, we found a differential regulation of plasma sST2 levels between sexes: while plasma sST2 level is lower in females than that in males, it exhibits a greater increase in AD in females (females, *β* = 2.235, FDR = 6.97 × 10^−4^; males, *β* = 1.926, FDR = 0.148; Extended Data Fig. 2a). In particular, female *APOE*-ε4 carriers with AD had the greatest increase of plasma sST2 level compared to that in other subgroups (*β* = 3.833, FDR = 0.004; Extended Data Fig. 2b,c and Supplementary Table 2). Moreover, females showed stronger associations between plasma sST2 level and AD-related endophenotypes (including gray matter volumes, plasma P-tau181 levels and plasma NfL levels) than those in males (Supplementary Table 2). These results suggest that plasma sST2 level is associated with AD and its related endophenotypes in a female-specific manner.

**Extended Data Fig. 3:**
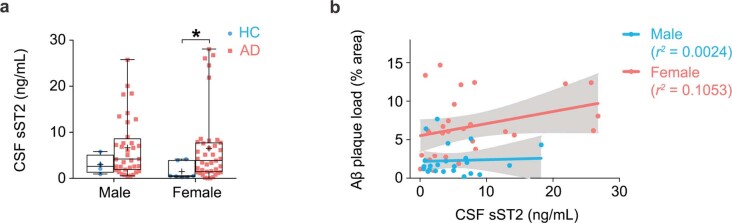
Associations between cerebrospinal fluid soluble ST2 levels and Alzheimer’s disease and amyloid-beta depositions in the UKBBN cohort, stratified by sex. (a) Individual cerebrospinal fluid (CSF) soluble ST2 (sST2) levels stratified by sex and disease phenotype (*n* = 4 male healthy controls [HCs], *n* = 36 male individuals with Alzheimer’s disease [AD], *n* = 7 female HCs, *n* = 39 female individuals with AD). Test for effects of sex: *β* = −1.307 and −0.675 in HCs and individuals with AD, respectively; test for effects of AD: *β* = 4.019 and 7.766 in males and females, respectively. Data are presented as box-and-whisker plots including maximum, 75^th^ percentile, median, 25^th^ percentile, and minimum values; plus signs (+) denote corresponding mean values. Linear regression test, adjusted for age and postmortem duration (PMD), with multiple testing correction; *FDR < 0.05. (b) Associations between amyloid-beta (Aβ) staining in the postmortem frontal cortex and CSF sST2 levels in male and female individuals with AD (*n* = 23 males and 28 females in the UKBBN cohort, respectively). The regression lines and 95% confidence intervals are indicated in red/blue and gray, respectively. Linear regression test, adjusted for age and PMD, with multiple testing correction. Test in males: *β* = 0.0256, Pearson’s *r*^*2*^ = 0.0024, *FDR* = 0.7866; test in females: *β* = 0.2188, Pearson’s *r*^*2*^ = 0.1053, *FDR* = 0.0407. *FDR*, false discovery rate.

We further showed that plasma and CSF sST2 levels are positively correlated within the same individual (*R*^*2*^ = 0.130, *P* < 0.0001; *n* = 107 individuals from the Stanford Alzheimer’s Disease Research Center (ADRC) cohort^35^; Fig. 1e). Accordingly, we performed a linear regression analysis between CSF sST2 level and AD and Aβ plaque load, adjusting for age, sex and postmortem duration (PMD), followed by multiple testing correction. Concordant with the regulation of plasma sST2 level in AD, in the UK Brain Banks Network (UKBBN) cohort (*n* = 11 HCs, *n* = 75 individuals with AD; Supplementary Data 1), the CSF sST2 level was higher in individuals with AD than in HCs (*β* = 6.605, FDR < 0.01; Fig. 1f), with a greater increase in females than that in males (females, *β* = 7.766, FDR = 0.034; males, *β* = 4.019, FDR = 0.241; Extended Data Fig. 3a). Moreover, CSF sST2 level was positively associated with Aβ plaque load in the frontal cortex in individuals with AD (*β* = 0.191, FDR = 0.031; Fig. 1g,h and Supplementary Fig. 1); this association was also stronger in females than in males (females, *β* = 0.219, FDR = 0.041; males, *β* = 0.026, FDR = 0.787; Extended Data Fig. 3b). Taken together, these findings suggest that sST2 levels in both the blood and CSF are increased in AD and associated with disease progression, specifically in female populations.

### sST2 level is associated with a genetic variant of *IL1RL1*

**Extended Data Fig. 4:**
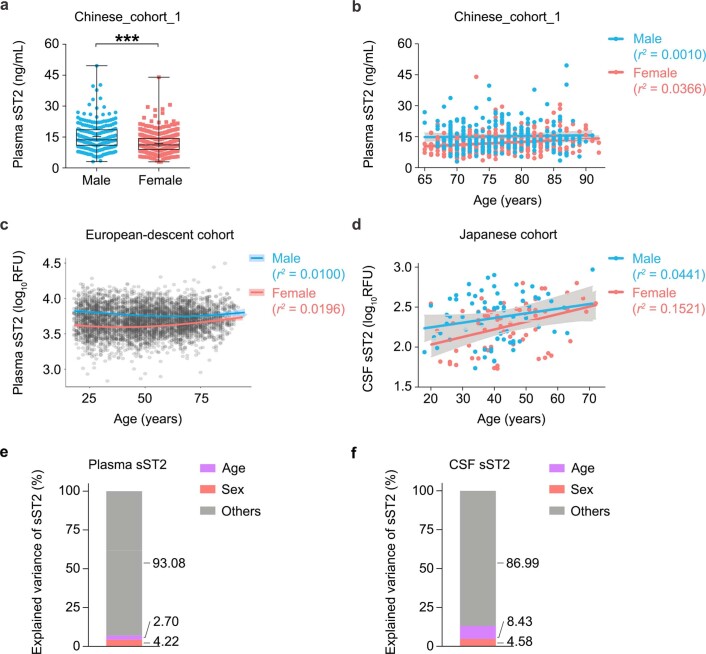
Effects of sex and age on soluble ST2 levels. (a) Individual plasma sST2 levels stratified by sex (*n* = 216 males, and 397 females from Chinese_cohort_1). *β* = −3.577 for females vs. males, *P* = 1.58E − 12. (b) Associations between plasma sST2 levels and age in males and females (*n* = 216 males, and 397 females from Chinese_cohort_1). Test in males: *β* = 0.0134, Pearson’s *r*^*2*^ = 0.0010, *P* = 0.8661; test in females: *β* = 0.1256, Pearson’s *r*^*2*^ = 0.0366, *P* = 0.0016. (c) Correlations between plasma sST2 level and age in males and females from the INTERVAL and LonGenity cohort (*n* = 1,685 males, and 1,616 females from INTERVAL cohort; *n* = 432 males, and 530 females from LonGenity cohort). Test in INTERVAL cohort (ages 18–76), males: Pearson’s *r*^*2*^ = 0.0169, *P* < 0.0001; females: Pearson’s *r*^*2*^ = 0.0001, *P* = 0.7715. Test in LonGenity cohort (ages 65–94), males: Pearson’s *r*^*2*^ = 0.0100, *P* = 0.0093; females: Pearson’s *r*^*2*^ = 0.0196, *P* = 0.0001. (d) Correlations between CSF sST2 level and age in males and females from the Japanese cohort (*n* = 68 males, *n* = 65 females). Test in males: Pearson’s *r*^*2*^ = 0.0441, *P* = 0.0812; test in females: Pearson’s *r*^*2*^ = 0.1521, *P* = 0.0014. (e, f) Contributions of age and sex to the variance of sST2 levels. Numbers denote the proportions of plasma sST2 (e) and CSF sST2 (f) variance explained by age, sex, and other factors in the Chinese_cohort_1 and Japanese cohort, respectively. Data in box-and-whisker plots are presented with maximum, 75^th^ percentile, median, 25^th^ percentile, and minimum values; plus signs (+) denote mean values; data in regression lines are presented as slope (red/blue) and 95% CIs (gray). Statistical tests were performed by linear regression analysis, additionally adjusted for AD diagnosis, CVD status, BMI, education level in the Chinese_cohort_1. *P < 0.05, **P < 0.01, ***P < 0.001.

To understand how sST2 is regulated in AD, we examined the contribution of various factors to the changes of sST2 levels. Specifically, we examined the associations between plasma/CSF sST2 levels and sex/age in three independent cohorts including Chinese_cohort_1, comprising HCs and individuals with AD, and a Japanese cohort^36^ and a European-descent cohort (that is, INTERVAL and LonGenity cohorts^37^) comprising cognitively normal individuals. Our results show that plasma sST2 level was lower in females than in males (*β* = −3.577, *P* < 0.001) and, compared to males (Chinese, *R*^2^ = 0.001, *P* = 0.866; European descent, *R*^2^ = 0.010, *P* = 0.009), females show a greater association between plasma sST2 level and age (Chinese, *R*^2^ = 0.037, *P* = 0.002; European descent, *R*^2^ = 0.020, *P* < 0.001; Extended Data Fig. 4a–c). Furthermore, CSF sST2 level was associated with both age and sex in the Japanese cohort, and the association with age, specifically, was greater in females (*R*^*2*^ = 0.152, *P* = 0.001) than in males (*R*^2^ = 0.044, *P* = 0.081; Extended Data Fig. 4d). These data suggest that sST2 level is modulated by age and sex in both East Asian and European-descent populations. However, factors such as age and sex accounted for only 6.92% and 13.01% of the variance of plasma sST2 levels and CSF sST2 levels, respectively (Extended Data Fig. 4e,f), suggesting that other factors modulate such differences.

**Fig. 2:**
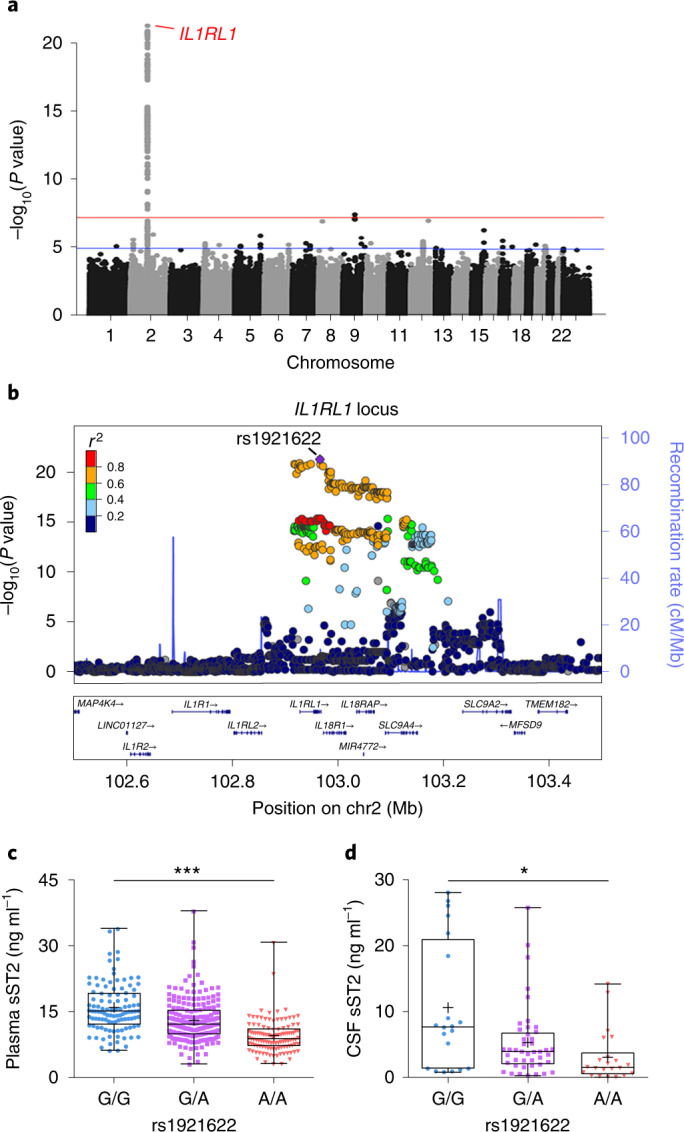
The rs1921622 A allele is associated with lower soluble ST2 level. **a**, Manhattan plot showing genetic variants at the *IL1RL1* locus that are associated with plasma sST2 level, as identified by a GWAS of plasma sST2 levels in Chinese_cohort_1. Horizontal lines indicate the suggestive threshold (*P* = 1 × 10^−5^, blue) and genome-wide threshold (*P* = 5 × 10^−8^, red). Linear regression test, adjusted for age, sex, AD diagnosis and population structure. **b**, Regional association plot of genetic variants at the *IL1RL1* locus and plasma sST2 level. The purple diamond indicates the sentinel variant rs1921622. The color scale indicates the linkage disequilibrium (LD; measured as *r*^2^) between rs1921622 and its neighboring variants. **c**,**d**, Plasma (**c**) and CSF (**d**) sST2 levels in individuals stratified by rs1921622 genotype. Measurement of plasma sST2 level (*n* = 107, 206 and 114 G/G, G/A and A/A carriers, respectively; Chinese_cohort_1). Linear regression test, adjusted for age, sex, AD diagnosis and population structure; *β* = −3.346, *P* = 5.35 × 10^−22^. Measurement of CSF sST2 level (*n* = 20, 44 and 22 G/G, G/A and A/A carriers, respectively; UKBBN cohort). Linear regression test, adjusted for age, sex, AD diagnosis and PMD; *β* = −2.244, *P* = 1.06 × 10^−2^. Data in box-and-whisker plots include maximum, 75th percentile, median, 25th percentile and minimum values; plus signs denote corresponding mean values. *P < 0.05, **P < 0.01, ***P < 0.001. Source data

Pilot association studies have identified several genetic variants in the *IL1RL1* gene that are associated with plasma sST2 level^38,39^, suggesting that genetic factors contribute to the regulation of sST2 level. Nonetheless, given that these identified SNPs form haplotype structures in this gene region^40^, these SNPs might simply be inherited together with the causal variants. Therefore, to identify the key genetic modulator(s) of sST2, we used our whole-genome sequencing (WGS) dataset^41^ of Chinese_cohort_1 to perform a GWAS of plasma sST2 levels, adjusting for age, sex, AD diagnosis and population structure. Accordingly, we identified 575 genetic variants that were significantly associated with plasma sST2 level (*P* < 1 × 10^−5^) and found that these variants accounted for 54.86% of the variation thereof (Fig. 2a and Supplementary Fig. 2a). Among these 575 variants, 79 in or near *IL1RL1* that form a haplotype were most strongly associated with sST2 level (Fig. 2b, Supplementary Fig. 2b and Supplementary Data 2), and our fine-mapping analysis identified the sentinel variant rs1921622 (G/A) as the putative causal variant (with 99.9% probability; Supplementary Fig. 2c and Supplementary Table 3). In Chinese_cohort_1, the rs1921622 A allele was associated with a 20% decrease in plasma sST2 level in an allele dose-dependent manner (*β* = −3.346, *P* < 0.001; Fig. 2c). Moreover, CSF sST2 level was lower in rs1921622 A allele carriers than in noncarriers (UKBBN cohort; *β* = −2.244, *P* < 0.05; Fig. 2d). Notably, rs1921622 alone accounted for 18.04% and 18.29% of the variance in plasma and CSF sST2 levels, respectively, which is greater than the contributions of age and sex. Hence, our fine-mapping analysis using WGS data identified rs1921622 as a key genetic factor that modulates the plasma and CSF levels of sST2.

### The rs1921622 locus regulates sST2 in brain endothelial cells

**Fig. 3:**
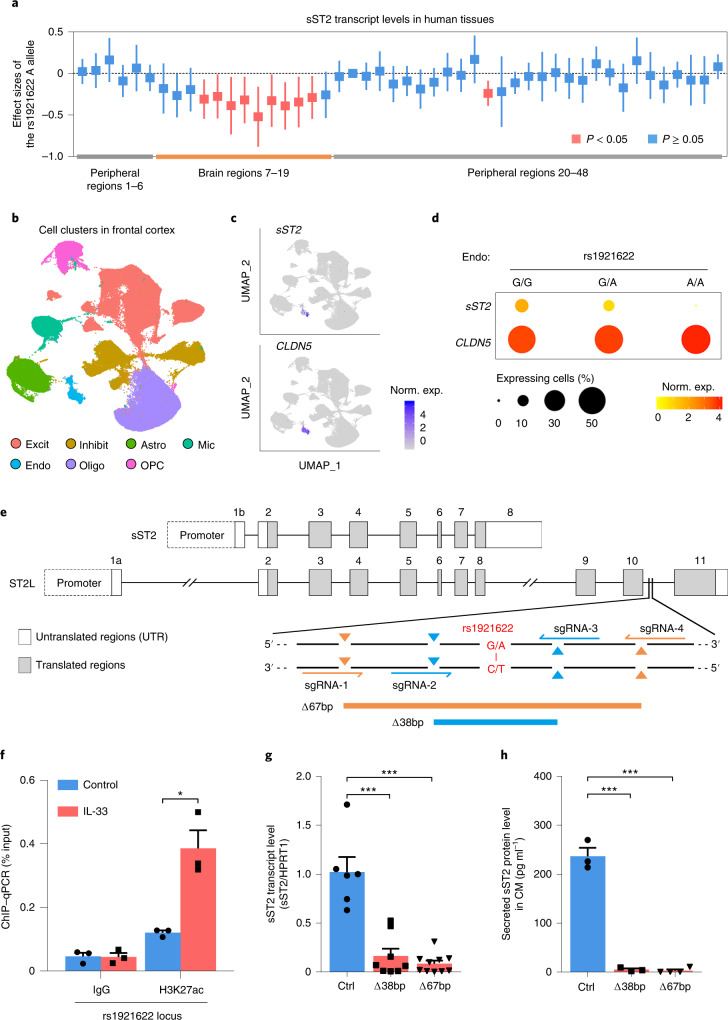
Target deletion at rs1921622 decreases soluble ST2 expression and secretion in brain endothelial cells. **a**, Effects of rs1921622 on sST2 transcript levels in human tissues. Boxes and lines indicate the effect size and 95% CIs of the rs1921622 A allele for each tissue, respectively (Supplementary Table 4). Red and blue indicate significant (*P* < 0.05) and nonsignificant (*P* ≥ 0.05) associations, respectively. Linear regression test, adjusted for age, sex, RNA integrity and population structure. **b**–**d**, snRNA-seq analysis revealed an association between rs1921622 and sST2 transcript level in brain endothelial cells. **b**, Uniform manifold approximation and projection (UMAP) plot showing cell types in the human frontal cortex (*n* = 169,496 cells from 21 individuals; UKBBN cohort). Excit, excitatory neurons; Inhibit, inhibitory neurons; Astro, astrocytes; Mic, microglia; Endo, endothelial cells; Oligo, oligodendrocytes; OPCs, oligodendrocyte progenitor cells. **c**,**d**, Expression profiles (**c**) and dot plots (**d**) of *sST2* and *CLDN5* transcripts in the endothelial cells, stratified by rs1921622 genotype. Norm. exp., normalized expression. **e**, CRISPR–Cas9 genome-editing strategy and locations of the two single-guide RNA (sgRNA) pairs for 67-bp deletion (Δ67 bp) and 38-bp deletion (Δ38 bp) targeting the rs1921622-harboring region (red). **f**, H3K27ac analysis of the rs1921622 locus in hCMEC/D3 cells after IL-33 administration for 24 h (*n* = 3 per group). *T* = 4.593, *P* = 1.01 × 10^−2^. **g**,**h**, Deletion of the rs1921622 locus decreases transcript level and protein secretion of sST2 in hCMEC/D3 cells. **g**, sST2 transcript levels (*n* = 6, 8 and 10 clones for isogenic control, Δ38 bp and Δ67 bp, respectively). For Δ38 bp versus control, *T* = − 5.444, *P* = 1.00 × 10^−4^; Δ67 bp versus control, *T* = − 7.612, *P* < 1.00 × 10^−4^. **h**, Levels of sST2 protein in conditioned medium (CM; *n* = 3, 3 and 4 clones for isogenic control, Δ38 bp and Δ67 bp, respectively). For Δ38 bp versus control: *T* = −13.450, *P* = 2.00 × 10^−4^; Δ67 bp versus control: *T* = −16.030, *P* < 1.00 × 10^−4^. Data in bar charts are the mean + s.e.m. Statistical tests for **f**–**h** were performed as two-sided unpaired Student’s *t*-tests. *P < 0.05, **P < 0.01, ***P < 0.001.

As rs1921622 is a noncoding variant located in the intronic region of ST2L, which is downstream of the region encoding sST2, we examined whether it modulates the expression of sST2 and ST2L. Association analysis using the Genotype-Tissue Expression (GTEx) dataset^42,43^ showed that, compared to noncarriers, rs1921622 A allele carriers exhibited a lower transcript level of sST2, but not ST2L, in certain brain regions (for example, hippocampus and frontal cortex; *P* < 0.05; Fig. 3a and Supplementary Table 4). Furthermore, an analysis of our previously released human frontal cortex single-nucleus RNA-sequencing (snRNA-seq) dataset^44^ revealed that sST2 is exclusively expressed by endothelial cells (that is, *CLDN5*-expressing cells; Fig. 3b,c). In addition, cell-type-specific association analysis showed that, compared to noncarriers, rs1921622 A allele carriers had a lower endothelial cell sST2 transcript level (*P* < 0.01) and fewer sST2-expressing endothelial cells (*P* < 0.05; Fig. 3d). These results collectively indicate that the rs1921622 variant is associated with decreased sST2 expression in human brain endothelial cells.

To investigate the specific role of rs1921622 in the decreased sST2 expression in endothelial cells, we examined whether rs1921622 and the surrounding genomic region (Fig. 3e) regulate sST2 transcription. Given that noncoding variants commonly modulate gene expression by functioning as enhancer elements^45^, we examined enhancer activity at the rs1921622 locus in the human cerebral microvascular endothelial cell line (hCMEC/D3). Administration of the cytokine IL-33 increased the expression and secretion of sST2 in hCMEC/D3 cells (both *P* < 0.001; Supplementary Fig. 3a,b). Moreover, chromatin immunoprecipitation (ChIP) assay showed that these IL-33-treated hCMEC/D3 cells exhibit increased occupancy of an active enhancer histone mark (that is, acetylated histone H3 Lys27 (H3K27ac)) at the rs1921622 locus, with a concomitant higher level of trimethylated histone H3 Lys4 (H3K4me3) histone modification (indicating active promoter regions) at the sST2 promoter region (both *P* < 0.05; Fig. 3f and Supplementary Fig. 3c). These results suggest that rs1921622 is located at a potential enhancer element of the sST2 gene.

To demonstrate that the genomic region harboring the rs1921622 locus contributes to the regulation of sST2 expression, we deleted this region in hCMEC/D3 cells with a CRISPR–Cas9-based approach. We generated two different hCMEC/D3 cell lines with biallelic 38- or 67-base-pair (bp) deletions (Δ38 bp and Δ67 bp, respectively) encompassing the rs1921622 locus (Fig. 3e and Supplementary Fig. 4). Notably, loss of the 38 or 67 bp flanking the rs1921622 locus decreased the sST2 transcript level in hCMEC/D3 cells (*P* < 0.001; Fig. 3g) and concomitantly abolished sST2 protein secretion (*P* < 0.001; Fig. 3h). These results suggest that the rs1921622-containing region plays a putative regulatory role as an enhancer element that regulates sST2 expression in endothelial cells.

### The rs1921622 A allele protects against Alzheimer’s disease in female *APOE*-ε4 carriers

**Fig. 4:**
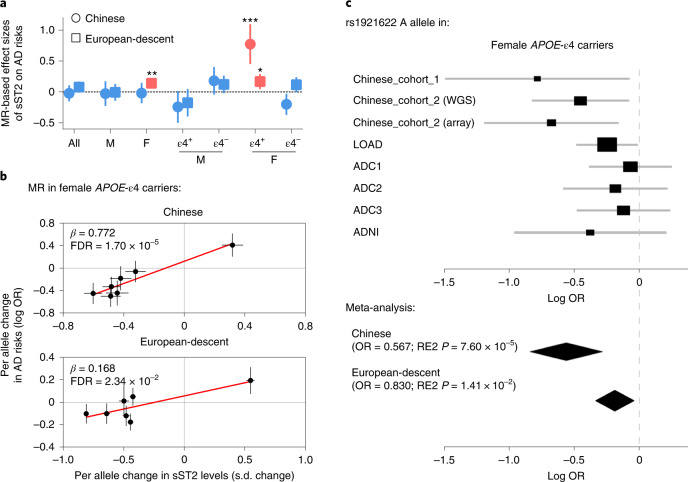
The rs1921622 A allele is associated with decreased Alzheimer’s disease risk in female *APOE*-ε4 carriers. **a**, Two-sample MR analysis showing the effects of sST2 levels on AD risk in Chinese (circle) and European-descent (box) populations. Circles/boxes and lines indicate the effect sizes of sST2 and 95% CIs in each subgroup, respectively (Supplementary Table 6). Red and blue indicate significant (FDR < 0.05) and nonsignificant (FDR ≥ 0.05) associations, respectively. All, overall population; M, male; F, female; ε4^+^, *APOE*-ε4 carriers; ε4^−^, *APOE*-ε4 noncarriers. **b**, Two-sample MR analysis showing the associations between sST2 level and AD risk in female *APOE*-ε4 carriers in Chinese and European-descent populations. Circles and lines indicate the effect sizes and standard errors of each SNP, respectively. **c**, Forest plot showing the meta-analysis results of the rs1921622 A allele on AD risk in female *APOE*-ε4 carriers (*n* = 912 HCs, *n* = 1,898 individuals with AD). Rectangles and diamonds denote the effect sizes (log OR) obtained from independent datasets and meta-analysis, respectively. For the independent datasets, horizontal lines indicate 95% CIs, and rectangle size is proportional to the weight used in the meta-analysis. RE2 *P*, meta-analysis *P* value using Han and Eskin’s random-effects model. *FDR < 0.05, **FDR < 0.01, ***FDR < 0.001.

Dysregulation of a specific protein in a disease may indicate that the protein is a causative factor of the disease, or its altered level may simply be due to tissue damage/reaction. Notably, for a protein that is regulated by genetic variants, MR analysis examining the associations between the protein with its genetic modulators and a disease would help illustrate whether the protein has disease-causing effects^46^. Therefore, we investigated the causality between sST2 level and AD risk by examining the associations between the genetic modulators of sST2 (including rs1921622) and AD risk and its related endophenotypes. Specifically, we performed the two-sample MR analysis of sST2 on AD risk using eight AD datasets as the discovery cohorts: the Chinese_cohort_1 dataset, the WGS and array datasets of the Chinese_cohort_2 (ref. ^47^) and five public datasets from populations of European descent (that is, the Late Onset Alzheimer’s Disease (LOAD)^48^, Alzheimer’s Disease Center 1–3 (ADC1–3)^49,50^ and Alzheimer’s Disease Neuroimaging Initiative (ADNI) datasets; *n* = 5,477 HCs, *n* = 5,910 individuals with AD; Supplementary Tables 1 and 5). The results show that while sST2 level was not associated with AD risk in all individuals (Chinese, *β* = −0.023, FDR = 0.861; European descent, *β* = 0.077, FDR = 0.131; Fig. 4a and Supplementary Table 6), increased sST2 level was associated with increased AD risk in female *APOE-*ε4 carriers in both Chinese and European-descent populations (Chinese, *β* = 0.772, FDR = 1.70 × 10^−5^; European-descent, *β* = 0.168, FDR = 0.023; Fig. 4a,b and Supplementary Table 6). In particular, among the genetic modulators of sST2, the top variant, the rs1921622 A allele, exerted an AD-protective effect in female *APOE-*ε4 carriers (odds ratio (OR) = 0.757, meta-analysis *P* value using Han and Eskin’s random-effects model (RE2 *P*)^51^ = 7.78 × 10^−5^, FDR = 5.44 × 10^−4^; Fig. 4c), whereas this effect is absent in the overall population (OR = 0.942, RE2 *P* = 0.044, FDR = 0.102) or other subgroups including male *APOE-*ε4 carriers (OR = 1.086, RE2 *P* = 0.412, FDR = 0.577), male *APOE-*ε4 noncarriers (OR = 0.960, RE2 *P* = 0.586, FDR = 0.684) and female *APOE-*ε4 noncarriers (OR = 0.961, RE2 *P* = 0.233, FDR = 0.408; Supplementary Tables 7 and 8). Interestingly, both the MR analysis of sST2 and the meta-analysis of rs1921622 showed that the associations between AD risk and sST2/rs1921622 in female *APOE-*ε4 carriers are stronger in the Chinese population than those in the European-descent populations (Chinese versus European descent, *P* = 5.55 × 10^−4^ and 0.017 for the MR analysis of sST2 and meta-analysis of rs1921622, respectively; Fig. 4a–c and Supplementary Tables 6–8), suggesting an ethnic-specific effect of sST2/rs1921622 in modulating AD risk.

**Fig. 5:**
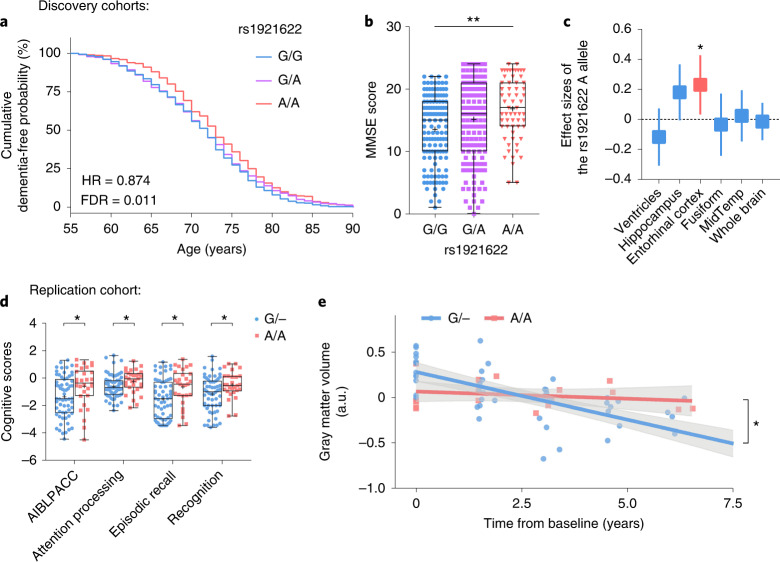
The rs1921622 A allele is associated with changes of Alzheimer’s disease-related endophenotypes in female *APOE*-ε4 carriers. **a**, Cumulative dementia-free probability in female *APOE*-ε4 carriers with AD stratified by rs1921622 genotype (*n* = 314, 568 and 300 G/G, G/A and A/A carriers, respectively; LOAD and ADC1–3 cohorts). Cox regression test, adjusted for population structure, with multiple testing correction. **b**, Individual Mini-Mental State Exam (MMSE) scores of female *APOE*-ε4 carriers with AD stratified by rs1921622 genotype (*n* = 131, 158 and 61 G/G, G/A and A/A carriers, respectively; Chinese_cohort_2). Linear regression test, adjusted for age and population structure, with multiple testing correction. *β* = 1.622. **c**, Effect size of the rs1921622 A allele on brain region volume in female *APOE-*ε4 carriers with cognitive impairment (*n* = 38, 86 and 57 G/G, G/A and A/A carriers, respectively; ADNI cohort). Linear regression test, adjusted for age, ICV, magnetic resonance imaging (MRI) platforms, dementia stages and population structure, with multiple testing correction. Data are presented as effect sizes (boxes) and 95% CIs (lines). Red and blue indicate significant (FDR < 0.05) and nonsignificant (FDR ≥ 0.05) associations, respectively. Fusiform, fusiform gyrus; MidTemp, middle temporal gyrus. **d**, Individual cognitive scores in female Aβ^+^ *APOE*-ε4 carriers stratified by rs1921622 genotype (*n* = 59 G carriers (G/−), *n* = 29 A/A carriers; AIBL cohort). Wilcoxon rank-sum test, with multiple testing correction. *W* = 1087.0, 1115.5, 1083.0 and 916.5 for AIBLPACC, attention processing, episodic recall and recognition scores, respectively. **e**, Longitudinal gray matter volume in female Aβ^+^ *APOE*-ε4 carriers stratified by rs1921622 genotype (*n* = 50 and 19 data points from 12 G carriers (G/−) and 5 A/A carriers, respectively; AIBL cohort). Linear mixed model test; adjusted for baseline age and MRI scanners, with multiple testing correction. *β* = −0.159 (G/−) and −0.027 (A/A); *F* = 8.804. Data in box-and-whisker plots are presented with maximum, 75th percentile, median, 25th percentile and minimum values; plus signs denote mean values; data in regression lines are presented as the slope (red/blue) and 95% CIs (gray). *FDR < 0.05, **FDR < 0.01, ***FDR < 0.001. a.u., arbitrary units.

Next, we examined the effects of the rs1921622 A allele on the AD-associated endophenotypes in the discovery cohorts. Consistent with its AD-protective effects (Supplementary Table 9), the rs1921622 A allele was associated with delayed onset age of dementia (hazard ratio (HR) = 0.874, FDR = 0.011; Fig. 5a), better cognitive scores (*β* = 1.622, FDR = 0.001; Fig. 5b) and larger entorhinal cortex volumes (*β* = 0.214, FDR = 0.027; Fig. 5c) in female *APOE-*ε4 carriers.

**Extended Data Fig. 5:**
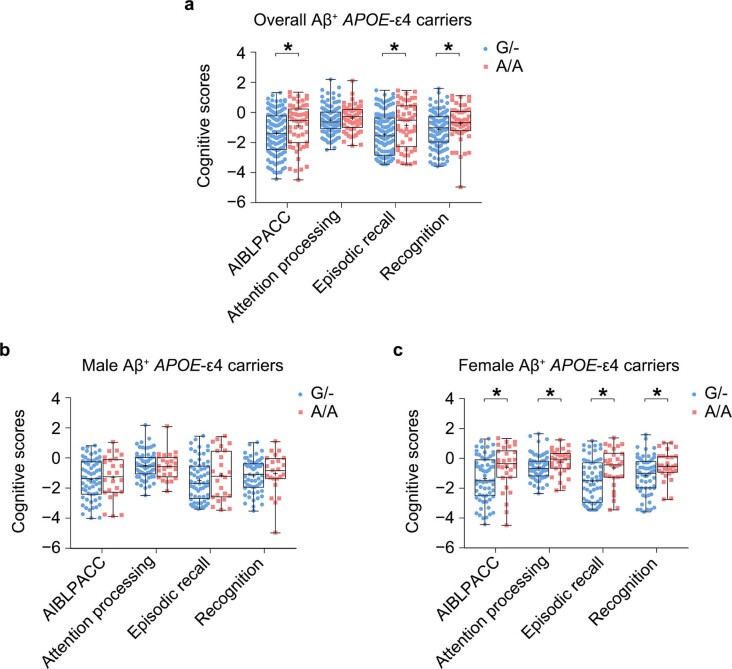
The rs1921622 A allele is associated with better cognitive performance in female *APOE*-ε4 carriers in the AIBL Aβ^+^ cohort. Individual cognitive scores in (a) overall Aβ^+^ *APOE*-ε4 carriers (*n* = 122 G carriers [G/-], *n* = 53 A/A carriers), (b) male Aβ^+^ *APOE*-ε4 carriers (*n* = 63 G carriers [G/-], *n* = 24 A/A carriers), and (c) female Aβ^+^ *APOE*-ε4 carriers (*n* = 59 G carriers [G/-], *n* = 29 A/A carriers) stratified according to rs1921622 genotype. Test in the overall Aβ^+^ *APOE*-ε4 carriers, *W* = 3782.0, 3671.0, 3842.0, and 3319.0 for the effects of rs1921622 genotype on AIBL Preclinical Alzheimer Cognitive Composite (AIBLPACC) score, attention processing score, episodic recall score, and recognition score, respectively; test in the male Aβ^+^ *APOE*-ε4 carriers, *W* = 784.0, 727.0, 823.5, and 740.0 for the effects of rs1921622 genotype on AIBLPACC score, attention processing score, episodic recall score, and recognition score, respectively; test in the female Aβ^+^ *APOE*-ε4 carriers, *W* = 1087.0, 1115.5, 1083.0, and 916.5 for the effects of rs1921622 genotype on AIBLPACC score, attention processing score, episodic recall score, and recognition score, respectively. Data in box-and-whisker plots are presented with maximum, 75^th^ percentile, median, 25^th^ percentile, and minimum values; plus signs (+) denote mean values. Statistical tests are performed by Wilcoxon rank-sum test, with multiple testing correction. *FDR < 0.05, **FDR < 0.01, ***FDR < 0.001. *FDR*, false discovery rate.

**Extended Data Fig. 6:**
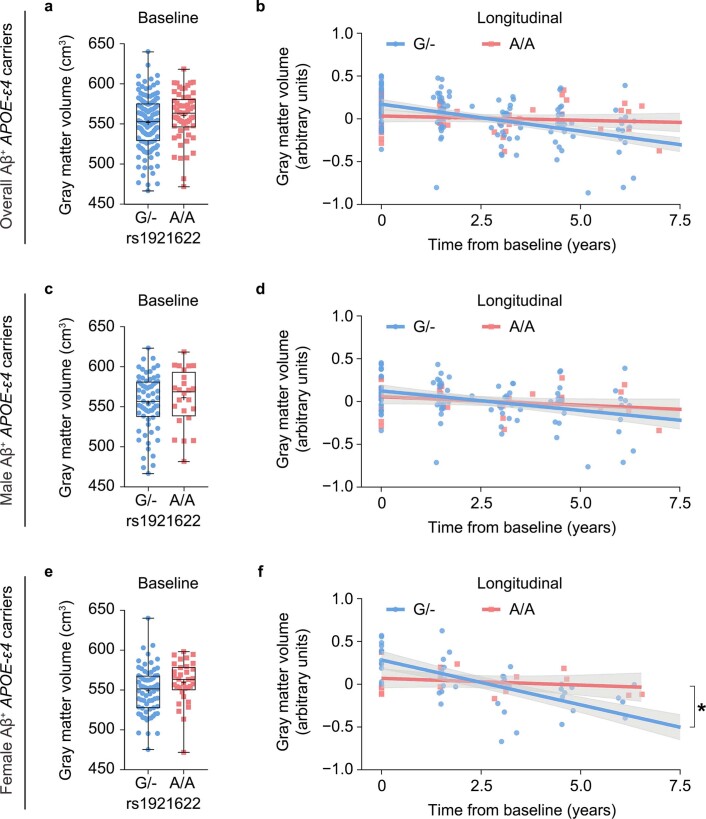
The rs1921622 A allele is associated with slower gray matter atrophy in female *APOE*-ε4 carriers in the AIBL Aβ^+^ cohort. (a, b) Individual baseline (a: *n* = 128 G carriers [G/-], and 55 A/A carriers) and longitudinal gray matter volume (b: *n* = 148 datapoints from 37 G/- carriers, and 55 datapoints from 14 A/A carriers) in overall Aβ^+^ *APOE*-ε4 carriers stratified by rs1921622 genotype. Test on baseline data: *W* = 4109.0, *FDR* = 0.109. Test on longitudinal data: *β* = −0.089 and −0.051 in G/- and A/A carriers, respectively; *F* = 3.830, *FDR* = 0.087. (c, d) Individual baseline (c: *n* = 64 G/- carriers, and 24 A/A carriers) and longitudinal gray matter volume (d: *n* = 98 datapoints from 25 G/- carriers, and 36 datapoints from 9 A/A carriers) in male Aβ^+^ *APOE*-ε4 carriers stratified by rs1921622 genotype. Test on baseline data: *W* = 852.0, *FDR* = 0.434. Test on longitudinal data: *β* = −0.069 and −0.053 in G/- and A/A carriers, respectively; *F* = 0.272, *FDR* = 0.607. (e, f) Individual baseline (e: *n* = 64 G/- carriers, and 31 A/A carriers) and longitudinal gray matter volume (f: *n* = 50 datapoints from 12 G/- carriers, and 19 datapoints from 5 A/A carriers) in female Aβ^+^ *APOE*-ε4 carriers stratified by rs1921622 genotype. Test on baseline data: *W* = 1240.0, *FDR* = 0.109. Test on longitudinal data: *β* = −0.159 and −0.027 in G/- and A/A carriers, respectively; *F* = 8.804. Data in box-and-whisker plots are presented with maximum, 75^th^ percentile, median, 25^th^ percentile, and minimum values; plus signs (+) denote mean values; data in regression lines are presented as slope (red/blue) and 95% confidence intervals (gray). Statistical tests on baseline data were performed by Wilcoxon rank-sum test, with multiple testing correction; and statistical tests on longitudinal data were performed by linear mixed model test, adjusted for baseline age and MRI scanners, with multiple testing correction. *FDR < 0.05, **FDR < 0.01, ***FDR < 0.001.

To further confirm the AD-protective effects of the rs1921622 A allele, we examined AD-related endophenotypes in an independent replication cohort: the Australian Imaging, Biomarkers and Lifestyle cohort (AIBL; *n* = 190), in which the Aβ deposition (that is, Aβ^+^) in the brains of individuals was confirmed by positron emission tomography^52^. Concordant with our findings from the discovery cohorts, in female Aβ^+^ *APOE-*ε4 carriers, the presence of the rs1921622 A allele was associated with improved cognitive performance as indicated by AIBL Preclinical Alzheimer Cognitive Composite (AIBLPACC) score and scores related to cognitive subprocesses including attention processing, episodic recall and recognition (all FDR < 0.05; Fig. 5d and Extended Data Fig. 5). Importantly, in a subgroup of female Aβ^+^ *APOE-*ε4 carriers whose gray matter volume was traced for 7 years, rs1921622 A allele carriers showed a slower progression of gray matter atrophy (*β* = −0.027) than that in noncarriers (*β* = −0.159; FDR < 0.05; Fig. 5e and Extended Data Fig. 6). Thus, these results validate the protective effects of the rs1921622 A allele against cognitive decline and gray matter atrophy among female *APOE-*ε4 carriers in an independent Aβ^+^ cohort.

### The rs1921622 A allele is associated with microglial activation in female *APOE*-ε4 carriers with Alzheimer’s disease

**Fig. 6:**
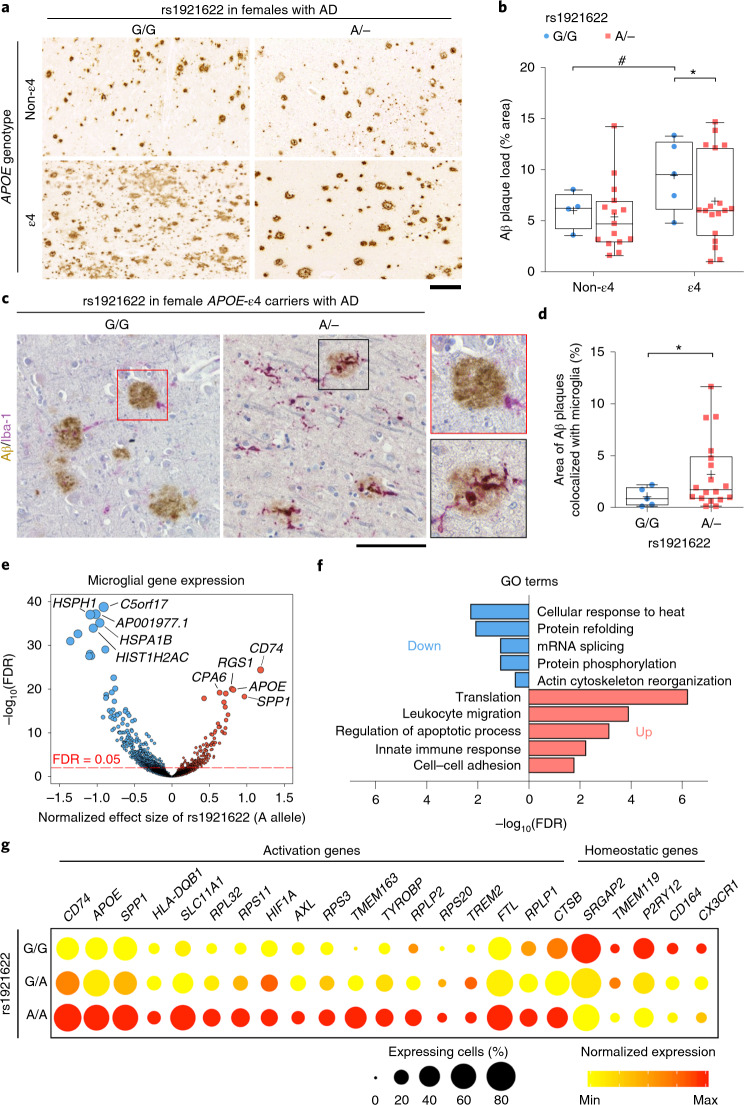
The rs1921622 A allele enhances microglial activities toward Aβ in female *APOE*-ε4 carriers with Alzheimer’s disease. **a**,**b**, Representative images (**a**) and quantification (**b**) showing Aβ plaque area in the frontal cortices of females with AD stratified by *APOE*-ε4 and rs1921622 genotypes (*n* = 4 G/G carriers and 15 A carriers (A/−) among *APOE*-ε4 noncarriers (non-ε4); *n* = 5 G/G carriers and 18 A/− carriers among *APOE*-ε4 carriers (ε4); UKBBN cohort). Test for effects of *APOE*-ε4: *β* = 3.448 (G/G) and 1.549 (A/−), *P* = 4.83 × 10^−2^ (G/G) and 2.54 × 10^−1^ (A/−); test for effects of rs1921622, *β* = 0.288 (non-ε4) and −2.262 (ε4), *P* = 7.02 × 10^−^^1^ (non-ε4) and 3.71 × 10^−^^2^ (ε4). Scale bar, 200 μm. **c**,**d**, Representative images (**c**) and quantification (**d**) showing colocalization between Aβ plaques (brown) and Iba-1^+^ microglia (purple) in the frontal cortices of female *APOE*-ε4 carriers with AD stratified by rs1921622 genotype (*n* = 5 G/G carriers, *n* = 18 A/− carriers; UKBBN cohort). *β* = 2.017, *P* = 2.53 × 10^−2^. Scale bar, 100 μm. **e**, Volcano plot showing the associations between rs1921622 and microglial genes in the frontal cortices of female *APOE*-ε4 carriers with AD (*n* = 2,636 microglia of eight individuals; UKBBN cohort). Blue and red dots indicate microglial genes that were negatively and positively associated with the rs1921622 A allele, respectively. Dot size is proportional to FDR (log_10_ scale). The top five negatively and positively associated genes are labeled. **f**, Representative GO terms enriched in the rs1921622-associated microglial genes. Blue and red indicate GO terms enriched for the downregulated and upregulated genes, respectively, in rs1921622 A allele carriers. **g**, Dot plot showing the expression levels of microglial activation genes and homeostatic genes stratified by rs1921622 genotype. Data in box-and-whisker plots are presented with maximum, 75th percentile, median, 25th percentile and minimum values; plus signs denote mean values. Statistical tests were performed by linear regression analysis, adjusted for age and PMD. *P < 0.05, **P < 0.01, ***P < 0.001; ^#^*P* < 0.05, ^##^*P* < 0.01, ^###^*P* < 0.001. Source data

**Extended Data Fig. 7:**
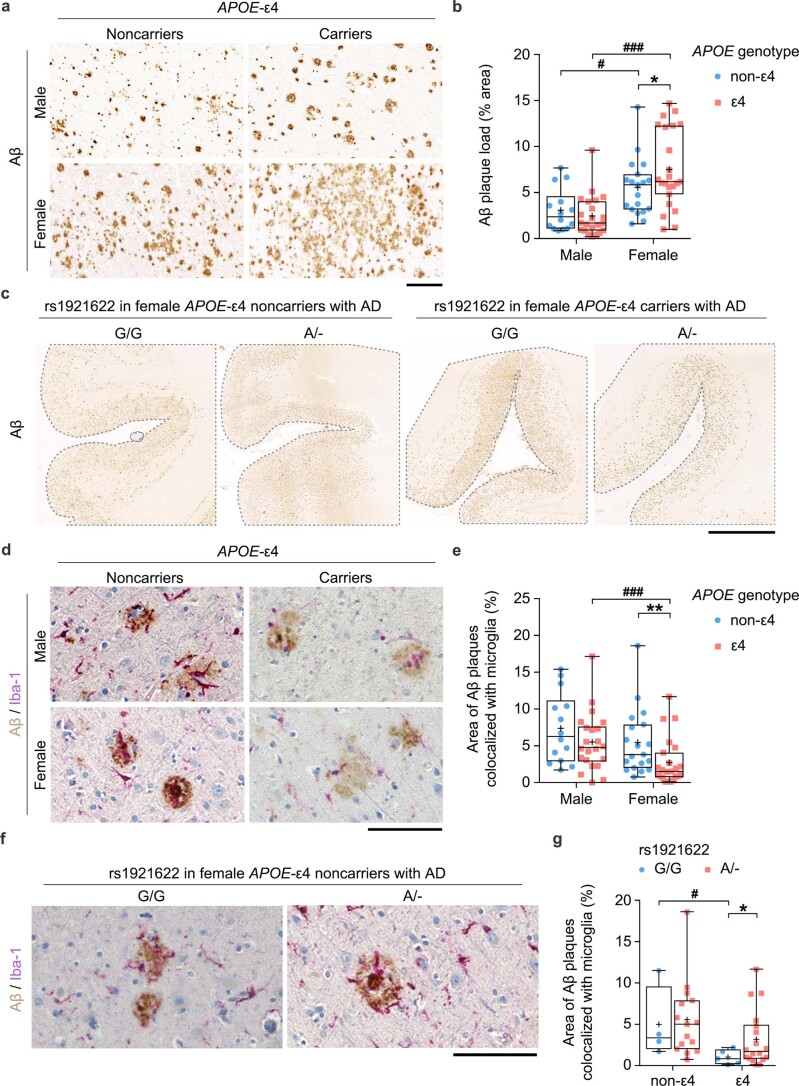
The rs1921622 A allele restores impaired microglial activities toward amyloid-beta in female *APOE*-ε4 carriers with Alzheimer’s disease. (a, b) Representative images (a) and quantification (b) of Aβ plaques in the frontal cortices of individuals with AD stratified by sex and *APOE*-ε4 genotype (*n* = 14 male *APOE-*ε4 noncarriers [non-ε4], 22 male *APOE*-ε4 carriers [ε4], 19 female non-ε4, and 23 female ε4 from UKBBN cohort). Test for sex: *β* = 2.871 (non-*APOE-*ε4) and 4.927 (*APOE-*ε4), *P* = 2.09E − 2 (non*-*ε4) and 4.61E − 5 (ε4); for *APOE*-ε4: *β* = −0.621 (males) and 1.967 (females), *P* = 4.22E − 1 (males) and 4.84E − 2 (females). Scale bar, 200 μm. (c) Representative images of Aβ plaques in the frontal cortices of females with AD stratified by *APOE*-ε4 and rs1921622 genotype. Scale bar, 5 mm. (d, e) Representative image (d) and quantification (e) of Aβ plaques (brown) and Iba-1^+^ microglia (purple) in the frontal cortices of individuals with AD stratified by sex and *APOE*-ε4 genotype (*n* = 14 male non-ε4, 22 male ε4, 19 female non-ε4, and 23 female ε4). Test for sex: *β* = −1.214 (non-ε4) and −4.133 (ε4), *P* = 4.61E − 1 (non-ε4) and 3.52E − 4 (ε4); for *APOE*-ε4: *β* = −0.965 (males) and −2.818 (females), *P* = 3.27E − 1 (males) and 6.00E − 3 (females). Scale bar, 100 μm. (f, g) Representative image (f) and quantification (g) of Aβ plaques and microglia in the frontal cortices of females with AD stratified by *APOE*-ε4 and rs1921622 genotype (*n* = 4 G/G and 15 A/- carriers among non-ε4, 5 G/G carriers and 18 A/- carriers among ε4, respectively). Test for *APOE*-ε4: *β* = −3.745 (G/G) and −1.944 (A/-), *P* = 3.09E − 2 (G/G) and 9.32E − 2 (A/-); for rs1921622: *β* = 0.781 (non-ε4) and 2.017 (ε4), *P* = 7.70E − 1 (non-ε4) and 2.53E − 2 (ε4). Scale bar, 100 μm. Data in box-and-whisker plots are presented with maximum, 75^th^ percentile, median, 25^th^ percentile, and minimum values; plus signs (+) denote mean values. Statistical tests were performed by linear regression analysis, adjusted for age, PMD. *P < 0.05, **P < 0.01, ***P < 0.001; ^#^*P* < 0.05, ^##^*P* < 0.01, ^###^*P* < 0.001.

Given our findings that the rs1921622 A allele exerts AD-protective effects in female *APOE-*ε4 carriers, we subsequently examined whether this variant modulates Aβ deposition in postmortem human brains. Among individuals with AD, specifically in females, the *APOE*-ε4 carriers exhibited greater Aβ deposition in the frontal cortex than that in the *APOE*-ε4 noncarriers (females, *β* = 1.967, *P* < 0.05; males, *β* = −0.621, *P* = 0.422; Extended Data Fig. 7a,b). However, upon further stratification by rs1921622 genotype, female *APOE*-ε4 carriers harboring the rs1921622 A allele exhibited less Aβ deposition than those without the allele (*β* = −2.262, *P* < 0.05; Fig. 6a,b and Extended Data Fig. 7c), suggesting that this allele attenuates the effects of *APOE*-ε4 on Aβ-related pathological changes in females. Moreover, immunohistochemical analysis revealed that harboring *APOE*-ε4 in AD is associated with less microglial coverage of Aβ plaques (that is, decreased proportion of Aβ colocalized with Iba-1^+^ microglia) in females (females, *β* = −2.818, *P* < 0.01; males, *β* = −0.965, *P* = 0.327; Extended Data Fig. 7d,e). In contrast, female *APOE*-ε4 carriers with AD who co-harbored the rs1921622 A allele showed increased colocalization between Iba-1^+^ microglia and Aβ plaques (*β* = 2.017, *P* < 0.05; Fig. 6c,d and Extended Data Fig. 7f,g). Thus, these results suggest that in female *APOE-*ε4 carriers with AD, the rs1921622 A allele is associated with enhanced microglia–Aβ interaction and decreased Aβ pathological lesions.

**Extended Data Fig. 8:**
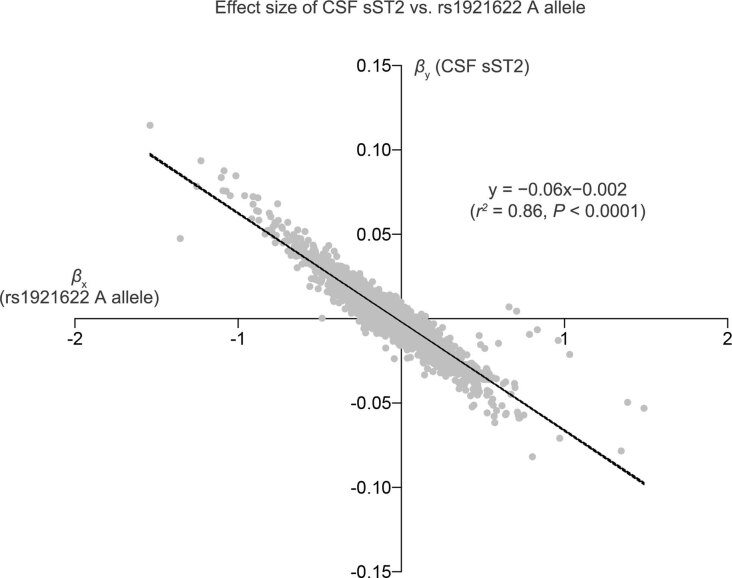
The rs1921622 A allele and cerebrospinal fluid soluble ST2 level exhibit opposite effects on the microglial transcriptome in female *APOE*-ε4 carriers with Alzheimer’s disease. Scatterplot showing the correlation between the normalized effect size (*β*) of cerebrospinal fluid (CSF) soluble ST2 (sST2) levels and the rs1921622 A allele on microglial gene expression in the frontal cortices of female *APOE*-ε4 carriers with Alzheimer’s disease (AD) (*n* = 2,636 microglia from 8 individuals from the UKBBN cohort).

Next, to investigate the regulatory effects of rs1921622 on microglial activities at the molecular level, we conducted an association analysis using the microglial snRNA-seq dataset in the frontal cortices^44^. We found a negative correlation between the effects of the rs1921622 A allele and the effects of CSF sST2 level on modulating microglial gene expression in female *APOE*-ε4 carriers with AD (*R*^*2*^ = 0.860, *P* < 0.0001; Extended Data Fig. 8), supporting the notion that the variant exerts its modulatory effects on microglia through the regulation of CSF sST2 level. Specifically, we identified 1,639 microglial genes that were associated with rs1921622 genotype: 428 and 1,211 genes were upregulated and downregulated, respectively, in individuals carrying the rs1921622 A allele compared to noncarriers (FDR < 0.05; Fig. 6e). Gene Ontology (GO) analysis showed that those upregulated genes are associated with leukocyte migration (FDR = 1.2 × 10^−4^) and innate immune response (FDR = 5.4 × 10^−3^), whereas those with downregulated expression are mainly involved in protein refolding (FDR = 8.1 × 10^−3^) or mRNA splicing (FDR = 7.7 × 10^−2^; Fig. 6f).

Recent studies of microglia in mouse and human brains revealed a subset of ‘microglial activation genes’, including *CD74*, *APOE* and *TREM2*, whose expressions are upregulated in AD^53,54^ and which are involved in microglial Aβ phagocytosis^4,55,56^. Therefore, we investigated whether these genes are associated with rs1921622. Interestingly, among female *APOE*-ε4 carriers with AD, the rs1921622 A allele was associated with increased expression of these microglial activation genes—specifically, increased transcript levels of *CD74*, *APOE* and *TREM2* in microglia as well as an increased proportion of *TMEM163*^+^ microglia (Fig. 6g). In contrast, the rs1921622 A allele was associated with decreased expression of homeostatic genes including *SRGAP2*, *TMEM119* and *P2RY12*, whose expressions commonly indicate a less-reactive microglial state^4,55^ (Fig. 6g). Therefore, these results suggest that the rs1921622 A allele promotes the transition of microglia to a more activated state in female *APOE*-ε4 carriers with AD.

### sST2 treatment impairs microglial Aβ-clearance capacity in female mouse brains

**Fig. 7:**
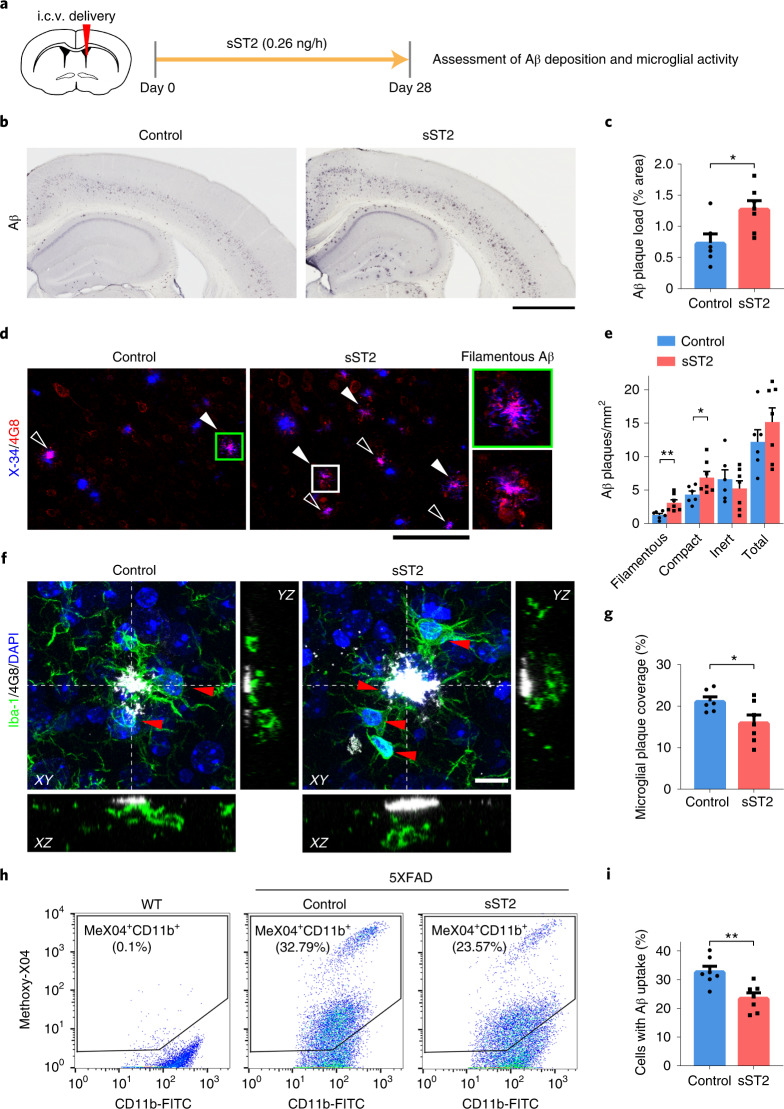
Increased brain soluble ST2 levels exacerbate Aβ accumulation and impair microglial Aβ clearance in female 5XFAD mice. **a**, Intracerebroventricular (i.c.v.) delivery of sST2 to 3-month-old 5XFAD mice. **b**–**e**, Aβ deposition in the cortices of 4-month-old female 5XFAD mice after i.c.v. delivery of sST2-Fc or Fc as a control. **b**,**c**, Representative images (**b**) and quantification (**c**) of Aβ plaques (percentage of total cortical area; control *n* = 6 mice, sST2 *n* = 7 mice). *T* = 2.758, *P* = 1.86 × 10^−2^. Scale bar, 1 mm. **d**, Confocal images of X-34-stained (blue) and 4G8-labeled (red) Aβ. Filamentous (filled arrowheads) and compact (hollow arrowheads) Aβ plaques. Scale bar, 100 μm. **e**, Quantification of filamentous, compact, inert and total Aβ plaques (control *n* = 6 mice, sST2 *n* = 7 mice). *T* = 3.235, 2.274, −0.770 and 1.050, respectively; *P* = 7.95 × 10^−3^, 4.40 × 10^−2^, 4.57 × 10^−1^ and 3.16 × 10^−1^, respectively. **f**, Merged confocal *z*-stack images with orthogonal *X–Z* and *Y*–*Z* views showing co-staining of 4G8-labeled Aβ plaques (white) and Iba-1^+^ microglia (green; red arrowheads indicate microglial soma) in the cortices of 4-month-old female 5XFAD mice after i.c.v. delivery of sST2-Fc or Fc as a control (single-channel images in Supplementary Fig. 7). Scale bar, 10 μm. **g**, Quantification of microglial coverage of Aβ plaques (control *n* = 6 mice, sST2 *n* = 7 mice). *T* = −2.298, *P* = 4.22 × 10^−2^. **h**,**i**, Microglial Aβ uptake activity in the cortices of 4-month-old female 5XFAD mice after i.c.v. delivery of sST2-Fc or Fc as a control. Representative scatterplots (**h**) and quantification (**i**) show the percentages of CD11b^+^ cells containing methoxy-X04-labeled Aβ (control *n* = 7 mice, sST2 *n* = 7 mice; gating strategy in Supplementary Fig. 8). *T* = −3.620, *P* = 3.50 × 10^−3^. The scatterplots of wild-type (WT) mice in **h** were used to gate methoxy-X04^+^ microglia (that is, MeX04^+^CD11b^+^ cells). Data in bar charts are the mean + s.e.m. Statistical tests were performed as two-sided unpaired Student’s *t*-tests. *P < 0.05, **P < 0.01, ***P < 0.001.

**Extended Data Fig. 9:**
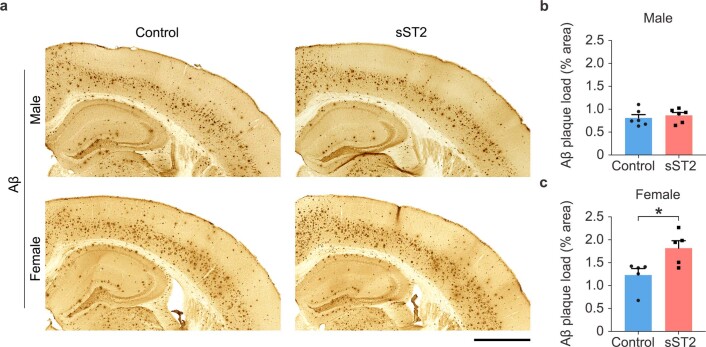
Elevated brain soluble ST2 level exacerbates amyloid-beta deposition in female but not male 5XFAD mice. (a, b) Amyloid-beta (Aβ) deposition in the cortices of 4-month-old male and female 5XFAD mice after 28-day intracerebroventricular delivery of soluble sST2 (sST2)-Fc or Fc as a control. (a) Representative images of Aβ staining. Scale bar, 1 mm. (b) Quantification of Aβ plaques (% of total cortical area) (control: *n* = 6 male and 5 female mice, sST2: *n* = 6 male and 5 female mice). *T* = 0.590 (males) and 2.721 (females); *P* = 0.568 (males) and 0.026 (females). Data in bar charts are mean + SEM. Statistical tests were performed as two-sided unpaired Student’s *t*-tests. *P < 0.05, **P < 0.01, ***P < 0.001.

Our genetic analyses showed that sST2 and its genetic modulators modulate AD risk and related pathologies possibly by regulating microglial activation and Aβ clearance. To verify the pathogenic roles of sST2 in AD, we examined the effect of sST2 injection on Aβ-associated pathological changes in an amyloidosis transgenic 5XFAD mouse model (Fig. 7a). Intracerebroventricular administration of mouse sST2 to 3-month-old 5XFAD mice for 28 d increased the Aβ plaque burden in the cortical regions of female (*P* < 0.05; Fig. 7b,c) but not male transgenic mice (Extended Data Fig. 9). In particular, the quantities of both the filamentous form (that is, X-34^+^ diffuse fibrils without a dense core^57^) and the compact form (that is, X-34^+^ dense cores with 4G8 halos) of Aβ plaques were increased in sST2-treated female 5XFAD mice (*P* < 0.05; Fig. 7d,e and Supplementary Fig. 5). However, sST2 administration did not affect the burden of less-toxic inert Aβ plaques (that is, X-34^+^ dense cores without 4G8 labeling) or the total number of Aβ plaques.

**Extended Data Fig. 10:**
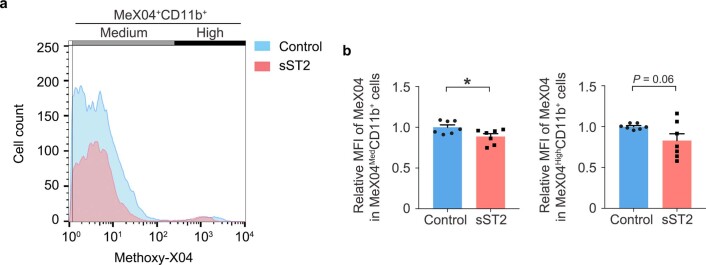
Elevated brain soluble ST2 level leads to decreased amyloid-beta phagocytosis by microglia in female 5XFAD mice. (a, b) Microglial amyloid-beta (Aβ) uptake activity in the cortices of 4-month-old female 5XFAD mice after 28-day intracerebroventricular delivery of soluble ST2 (sST2)-Fc or Fc as a control. Representative distribution (a) and quantification (b) show the mean fluorescent intensity (MFI) of methoxy-X04-labeled Aβ in the methoxy-X04^+^ CD11b^+^ microglia (control: *n* = 7 mice, sST2: *n* = 7 mice). *T* = − 2.490 and −2.014 for the tests in microglial with medium Aβ uptake (MeX04^Med^CD11b^+^; *left*) and high Aβ uptake (MeX04^High^CD11b^+^; *right*), respectively; *P* = 0.028 and 0.067, respectively. Data in bar charts are mean + SEM. Statistical tests were performed as two-sided unpaired Student’s *t*-tests. *P < 0.05, **P < 0.01, ***P < 0.001.

Next, we examined how sST2 regulates the interactions between microglia and Aβ plaques. In female 5XFAD mice, sST2 injection increased the total number of microglia, the clustering of microglia around Aβ plaques, and the number of proliferating (Ki67^+^) microglia in the cortical regions (all *P* < 0.05; Supplementary Fig. 6), suggesting that sST2 treatment promotes the proliferation of microglia in the brain. However, compared to the vehicle-injected condition, the cortical regions in sST2-injected female 5XFAD mice showed reduced microglial coverage of Aβ plaques (Fig. 7f,g, Supplementary Fig. 7 and Supplementary Video 1), suggesting that sST2 injection inhibits the microglial barrier formation around Aβ plaques. To verify the inhibitory effects of sST2 on microglial Aβ clearance, flow cytometry analysis showed that sST2 administration decreases the Aβ-phagocytic capacity of microglia as indicated by a decrease in the percentage of Aβ^+^ microglia (that is, 23.57% methoxy-X04^+^CD11b^+^ microglia in the sST2-treated group versus 32.79% in the control group; *P* < 0.01; Fig. 7h,i and Supplementary Fig. 8) and an 11.3–17.0% decrease in Aβ uptake by microglia (*P* < 0.05; Extended Data Fig. 10). Together, these findings demonstrate that increased brain sST2 level impairs microglial Aβ-clearance capacity and exacerbates Aβ accumulation in female transgenic mice, suggesting its pathogenic roles in female AD.

## Discussion

Here, we report that an endothelial cell-secreted protein, sST2, is upregulated in female individuals with AD. Meanwhile, endothelial expression of sST2 can be downregulated by the rs1921622 A allele, a genetic variant that exerts an AD-protective effect specifically in female *APOE*-ε4 carriers, a subgroup of people accounting for 24.3–33.6% and 32.5–50.4% of individuals with AD in Chinese and European-descent populations, respectively (Supplementary Tables 1 and 5). This variant is also associated with enhanced microglial activation, increased microglia–Aβ plaque interaction and decreased Aβ deposition. Taken together, we demonstrate that sST2 regulates microglial activities and that changes in sST2 levels in the brain milieu impair microglial Aβ-clearance capacity, thus modulating AD risk and its pathological changes.

As sST2 only comprises the extracellular domain of the functional receptor ST2L and is independently transcribed^22^, it is a decoy receptor for IL-33–ST2 signaling. ST2L expression is mainly found in microglia in the brains of both humans and mice^58–60^, and IL-33–ST2 signaling regulates microglial activities in tissue repair, Aβ clearance and synapse engulfment^24,25,61,62^. The expression profile of sST2 differs between humans and mice: in humans, sST2 is expressed by brain and peripheral endothelial cells^63–65^ and monocytes/macrophages^60,66^, mast cells^67^ and T cells^68^ in peripheral systems; while in mice, sST2 is expressed by microglia in the brain^59^ and fibroblasts^69^, mast cells^67^ and T cells^68^ in peripheral systems. However, given that sST2 is a secreted protein in the circulatory system^24,70^, increased sST2 level likely impacts microglial functions and AD-related pathological changes in both humans and mice by blocking the binding of IL-33 to ST2L on microglia. In this study, sST2 treatment perturbed the interaction between microglia and Aβ as well as the subsequent Aβ phagocytosis in a mouse model of amyloidosis (Fig. 7). This finding is consistent with our previous observation that in these mice, IL-33 administration activates IL-33–ST2 signaling and initiates microglial chemotaxis toward Aβ plaques, subsequently enhancing Aβ phagocytosis^24,25^. Further corroborating the pathological role of sST2 in microglia in AD, among female *APOE*-ε4 carriers with AD, those carrying the rs1921622 A allele (who have lower sST2 levels) exhibit enhanced microglia–Aβ plaque interactions and a smaller Aβ plaque area than noncarriers. Thus, perturbation of endogenous IL-33–ST2 signaling by sST2 may lead to impaired microglial chemotaxis, Aβ uptake and barrier formation, which subsequently contribute to AD pathogenesis.

What are the regulatory mechanisms of sST2? In mice, sST2 expression is regulated by a proximal promoter located upstream of the 5′ untranslated region of sST2, which is increased by oncogenes, serum and other mitogenic stimuli^69^. While the regulatory mechanisms of sST2 expression might be different between humans and mice, sST2 regulation in humans remains largely unknown. Our analyses in Chinese and European-descent populations corroborate findings from previous studies that sST2 levels in humans are associated with age, sex and genetic variants^37,38^, and reveal that genetic components play a dominant role in the regulation of sST2 levels, accounting for 54.86% of the variance of these levels. In particular, our GWAS and fine-mapping analyses identified rs1921622 as a putative causal variant associated with sST2 (causal probability of 99.99%), and our ChIP assay and CRISPR–Cas9 genome editing verified that the rs1921622-containing region is an enhancer element that regulates the sST2 gene in endothelial cells. Thus, as rs1921622 is a key genetic modulator of sST2, future investigations of the epigenetic events at the rs1921622 locus may help elucidate the regulatory mechanisms of sST2.

Single-nucleus transcriptomic profiling of postmortem human brains shows that sST2 is mainly expressed in brain endothelial cells (Fig. 3). Dysregulation of sST2 in the brain exacerbates AD-associated pathological changes, specifically by impairing the phagocytic capacity of microglia. Thus, we demonstrate a new pathogenic role of the brain vasculature in AD that regulates microglial activities via sST2. Indeed, apart from Aβ and tau-related pathological changes, neurovascular dysfunction occurs early in AD and is implicated in its pathogenesis^71^. While the exact pathological functions of this brain vasculature remain unclear, vasculature-secreted cytokines (for example, CXCL1) in the peripheral system regulate the activation and migration of immune cells, thereby mediating the immune response in tissues^72^. Similarly, emerging studies suggest that the brain vasculature serves as a critical source of soluble inflammatory proteins such as IL-1β, IL-6, IL-8, tumor necrosis factor, transforming growth factor and monocyte chemoattractant protein-1 (refs. ^73,74^). Therefore, besides sST2, other soluble factor-based cross-talk between the vasculature and other cell types likely occurs in the brain. Accordingly, identifying the proteins involved in this cross-talk may provide insights into novel pathological mechanisms of AD.

We show that sST2 plays a disease-causing role in AD in a female-specific manner. Indeed, emerging evidence suggests that sex is an important factor that underlies the heterogeneity of AD;^75^ compared to males, females are more likely to develop AD^76^ and experience faster cognitive decline after an MCI or AD diagnosis^76,77^. Given that sST2 inhibits IL-33–ST2 signaling and microglial activities, this sex-specific causal effect of sST2 in AD may be attributed to the differential regulation of IL-33–ST2 signaling and differential microglial activities between sexes. Concordant with previous studies^78^, we show that microglia in female individuals with AD, particularly those who carry *APOE*-ε4, have significantly lower Aβ phagocytic capacity when compared to male individuals (Extended Data Fig. 7). Meanwhile, compared to males, females show a greater increase of sST2 upon AD development. Hence, it would be interesting to examine whether increased sST2 level in female brains exhibits a stronger inhibitory effect on microglial activities and impairs their Aβ-clearance capacity. Moreover, sex hormones (for example, testosterone) regulate IL-33 expression in mast cells, maintaining a higher activity of IL-33–ST2 signaling in men^79^, which might protect men from being affected by increased sST2 levels. Together, increased sST2 levels, resulting from aging or the absence of the rs1921622 A allele, might have more detrimental impacts in microglial activities and functions in females, eventually leading to a greater risk of AD. Therefore, investigating the underlying mechanisms of this female-specific, disease-causing role of sST2 may be key to understanding the differential progression of AD between sexes.

In addition to its female-specific effects, our genetic analyses demonstrate that sST2/rs1921622 plays disease-causing effects in females with AD, specifically those carrying *APOE*-ε4, suggesting a potential interaction between IL-33–ST2 signaling and ApoE. In the brain, ApoE is mainly produced by astrocytes, and its expression is upregulated in microglia in AD^53,80^. Single-cell RNA-seq of amyloidosis mouse models revealed that a microglial subpopulation transitions from a homeostatic state to an activated state termed ‘disease-associated microglia’ or ‘activated response microglia’^4,55^, with increased expression of microglial activation genes (including *APOE*, *AXL*, *TREM2* and *CD74*)^53–56^ that are crucial regulators of phagocytic processes^81–84^. In contrast, perturbation of ApoE functions in the brain abolishes the induction of this microglial activation state, resulting in reduced Aβ phagocytic capacity^4,55^. Thus, ApoE-mediated microglial activation may protect against AD and be required for Aβ clearance and brain homeostasis. Notably, the presence of the rs1921622 A allele modulates the transition of microglia from a homeostatic state to a more activated state in female *APOE*-ε4 carriers with AD, characterized by the increased expression of the aforementioned microglial activation genes (Fig. 6). Therefore, IL-33–ST2 signaling and ApoE might converge to regulate the expression of these specific genes in microglia, thereby modulating the activation state and Aβ-clearance capacity of microglia. Interestingly, in the periphery, IL-33 administration ameliorates the formation of macrophage foam cells (lipid-laden macrophages) and the development of atherosclerotic plaques in the ApoE^−/−^ model of atherosclerosis^85^. Therefore, it would be of interest to determine whether the modulation of IL-33–ST2 signaling reduces the detrimental effects of *APOE*-ε4 on Aβ accumulation through the regulation of lipid metabolism in AD.

Our findings suggest that sST2 is a promising therapeutic target for AD. First, as sST2 is mainly expressed by endothelial cells, this enables cell-type-specific manipulation of sST2 expression, and that manipulation may not need to cross the blood–brain barrier. Second, sST2 levels are elevated in individuals with MCI or early-stage AD^24,32^, suggesting the potential applicability of sST2 in early intervention strategies. Third, the deletion of the rs1921622 locus in a human brain endothelial cell line with high efficacy demonstrates the feasibility of this approach to silence sST2 expression and secretion without disrupting the activities of ST2L; this is because the epigenetic and transcriptional controls of sST2 are distinct from those of ST2L^86^, and the rs1921622 A allele only modulates sST2 but not ST2L expression. Fourth, given our finding that the rs1921622 A allele is a common AD-associated variant, manipulations of sST2 by targeting this genetic variant could be developed for specific subgroups of individuals who have high sST2 levels (for example, female individuals who carry *APOE*-ε4 but not the rs1921622 A allele, accounting for 6.2–12.2% of individuals with AD), enabling patient stratification and precision medicine.

Nonetheless, there are some knowledge gaps regarding the functions and regulation of sST2 in AD. First, while MR analysis of cross-sectional studies shows that sST2 plays a causal role in AD, longitudinal studies investigating the associations between sST2 level and cognitive decline and/or AD risk in cognitively normal populations would help support the notion that sST2 has disease-causing effects in AD. Meanwhile, given that such effects are only observed in female *APOE*-ε4 carriers, they are likely masked in recent genetic studies of AD, in both the overall population (*P* = 0.881)^87^ and *APOE*-ε4 carriers (*P* = 0.798)^88^. Therefore, stratification of AD datasets according to sex and *APOE*-ε4 genotypes may help to better investigate the disease effects of sST2 or other AD-associated factors. Moreover, as we observed that sST2/rs1921622 has stronger modulatory effects on AD risks in the Chinese population than in the European-descent populations, validation of this finding by future studies using larger datasets would help clarify this potential ethnic-specific effect of sST2. Lastly, while an increased brain level of sST2 contributes to AD pathogenesis, given the positive correlation between plasma and CSF sST2 levels, it would be interesting to examine whether peripheral sST2, produced by peripheral endothelial cells^65^, monocytes/macrophages^60,66^, T cells^68^ and mast cells^67^, is blood–brain barrier permeable and plays a pathogenic role in AD. Indeed, recent studies show that several drugs (for example, sacubitril/valsartan) can reduce peripheral sST2 levels in individuals with heart failure^89^. It is of interest to examine whether these drugs can also regulate peripheral sST2 levels in individuals with AD and ameliorate the disease-related pathological changes.

In summary, we uncovered an alternative pathogenic mechanism of AD that involves microglial dysfunctions mediated by sST2. Dysregulation of endothelial cell-secreted sST2 leads to increased plasma and CSF levels of sST2 and impairs Aβ clearance by microglia, resulting in increased Aβ accumulation in AD. Furthermore, we identified an AD-protective genetic variant, rs1921622, which downregulates sST2 expression and attenuates the *APOE*-ε4-related risk and pathological changes of AD through the regulation of microglial signaling. Thus, a better understanding of how sST2—a biomarker and potential drug target for AD—is genetically regulated can aid the design of AD intervention strategies and clinical trials.

## Methods

### Recruitment for Chinese_cohort_1

This study was approved by the Joint Chinese University of Hong Kong–New Territories East Cluster Clinical Research Ethics Committee at the Prince of Wales Hospital, the Chinese University of Hong Kong and the Hong Kong University of Science and Technology (HKUST). All individuals provided written informed consent for both study enrollment and sample collection. Specifically, we recruited a total of 690 Hong Kong Chinese individuals ≥ 60 years old, including 345 individuals with AD and 345 HCs, who visited the Specialist Outpatient Department of the Prince of Wales Hospital at the Chinese University of Hong Kong from April 2013 to February 2018. We established a clinical diagnosis of AD based on the American Psychiatric Association’s Diagnostic and Statistical Manual of Mental Disorders, Fifth Edition^90^. All individuals underwent a medical history assessment, clinical assessment, cognitive and functional assessments using the MoCA test, and neuroimaging assessment by MRI^91,92^, and only those with cognitive dysfunctions (that is, those having Alzheimer’s dementia with MoCA score < 21) were included in the AD group in this study. Individuals with any notable neurological disease besides AD or a psychiatric disorder were excluded. We recorded age, sex, years of education, medical history and history of CVDs. We prepared DNA and plasma samples from whole-blood samples and stored them at −80 °C until use. We used T1-weighted MRI to retrieve brain imaging data from 192 individuals (*n* = 77 individuals with AD, *n* = 115 HCs) from the Prince of Wales Hospital. Raw imaging files were deidentified and processed by AccuBrain IV1.2 (BrainNow Medical Technology) for the analysis of gray matter volumes.

### UK Brain Banks Network dataset

We obtained the following samples of the Medical Research Council (MRC) UKBBN dataset (Bristol Brain Bank): CSF samples, frontal cortex sections, frozen frontal cortex tissues and genomic DNA samples (Supplementary Data 1), from South West Dementia Brain Bank (SWDBB), which receives approval from North Somerset and South Bristol Research Ethics Committee to operate as a research tissue bank. For our initial sample selection from the UKBBN dataset, we excluded individuals with other neurodegenerative diseases, severe vascular diseases (for example, stroke), an intoxicated state, infection, prions, severe inflammatory diseases (for example, autoimmune diseases), structural brain disorders, metabolic/nutritional diseases, trauma, delirium, genetic disorders (for example, Down syndrome) or other systemic diseases. For CSF samples, we further selected samples with a PMD ≤ 30 h, yielding a total of 86 individuals (*n* = 75 individuals with AD, *n* = 11 HCs). In addition, we obtained snRNA-seq data from frozen frontal cortical samples from the UKBBN (*n* = 12 individuals with AD, *n* = 9 HCs) from our previously published dataset^44^.

### Other cohorts and data for association studies

We obtained the following data for replication studies: (i) genomic, demographic and clinical data from Chinese_cohort_2 (ref. ^47^); (ii) genomic, demographic and clinical data from the LOAD Family Study^48^; (iii) genomic, demographic and clinical data from the National Institute on Aging (NIA) ADC cohorts^49,50^; (iv) genomic, demographic, clinical and brain imaging data from the ADNI cohort; (v) genomic, demographic and transcriptomic data from the GTEx dataset;^42,43^ (vi) plasma biomarker, CSF biomarker, demographic and clinical data from the Stanford ADRC cohort;^35^ (vii) plasma biomarker and demographic data from the INTERVAL and LonGenity cohorts^37^ retrieved from the online database (https://twc-stanford.shinyapps.io/aging_plasma_proteome/); (viii) CSF biomarker and demographic data from a Japanese cohort;^36^ and (ix) genomic, demographic, clinical and brain imaging data from the AIBL cohort^52^. Details related to sample and data collection are presented in the Supplementary Notes.

### DNA and plasma extraction from human blood samples

We collected whole-blood samples (3 ml) from individuals into K3EDTA tubes (VACUETTE). We centrifuged the samples at 2,000*g* for 15 min to separate the cell pellet and plasma. The plasma was collected, aliquoted and stored at −80 °C until use. We sent the cell pellets to the Centre for PanorOmic Sciences (Genomics and Bioinformatics Cores, University of Hong Kong) for genomic DNA extraction using the QIAsymphony DSP DNA Midi Kit (Qiagen) on a QIAsymphony SP platform (Qiagen). Genomic DNA was eluted with water or Elution Buffer ATE (Qiagen) and stored at 4 °C. We determined the DNA concentration by BioDrop µLITE+ (BioDrop).

### Measurement of protein levels in human samples and cell lines

We measured the plasma level of sST2 in 613 individuals from Chinese_cohort_1 (*n* = 277 individuals with AD, *n* = 336 HCs); CSF level of sST2 in 86 individuals from the UKBBN cohort (*n* = 75 individuals with AD, *n* = 11 HCs); and the level of sST2 secreted by hCMEC/D3 cells using the Human ST2/IL-33 R Quantikine ELISA Kit (DST200; R&D Systems). The plasma levels of NfL (*n* = 154 individuals with AD, *n* = 135 HCs) and P-tau181 (*n* = 156 individuals with AD, *n* = 134 HCs) in individuals from Chinese_cohort_1 were measured by the Quanterix Accelerator Laboratory using the Quanterix NF-light SIMOA Assay Advantage Kit (103186) and P-Tau 181 Advantage V2 Kit (103714), respectively^93^.

### Whole-genome sequencing and single-nucleotide polymorphism array for genotyping

We submitted DNA samples from 427 individuals from Chinese_cohort_1 (*n* = 233 individuals with AD, *n* = 194 HCs) to Novogene for library construction and WGS. The samples were sequenced on the Illumina HiSeq X platform (average depth: 5×), and individual genotypes were analyzed using the GotCloud pipeline^41^. The genotype results, which were stored in VCF files, were used for principal-component analysis. The top five principal components were generated by PLINK software (v1.9)^94^ with the following parameters: --pca header tabs, --maf 0.05, --hwe 0.00001 and --not-chr x y.

We subjected the rest of the genomic DNA samples, including 263 from Chinese_cohort_1 (*n* = 112 individuals with AD, *n* = 151 HCs) and 113 from the UKBBN cohort (*n* = 102 individuals with AD, *n* = 11 HCs), to SNP array for the genotyping of chr2: 102966067 (GRCh37/hg19), *APOE*-ε2 and *APOE*-ε4 using TaqMan Assays rs1921622, C___1226146_10, 4351376; rs7412, C___904973_10, 4351376; and rs429358, C___3084793_20, 4351376, respectively (Thermo Fisher Scientific). We performed real-time quantitative PCR (qPCR) using the 7500 Fast and QuantStudio 7 Flex Real-Time PCR System (Applied Biosystems). We stored the results in EDS files and input them into TaqMan Genotyper Software (Applied Biosystems) for the joint genotyping of SNPs.

### Immunohistochemical staining of postmortem human brain sections

We obtained formalin-fixed, paraffin-embedded, postmortem, frontal cortex sections from 78 individuals with AD from the UKBBN cohort. We first deparaffinized and rehydrated the sections with xylene and graded ethanol solutions. To stain Aβ, we treated the sections with formic acid at room temperature (RT) for 5 min and quenched endogenous peroxidase activity with a 3% hydrogen peroxide solution. We then incubated the sections with a mouse anti-human Aβ antibody (1:500 dilution; clone NAB228, SC-32277, Santa Cruz Biotechnology) overnight at 4 °C. After washing, we incubated the sections with horseradish peroxidase (HRP)-labeled anti-mouse IgG (QD440-XAKE, RTU, BioGenex) and developed signals with 3,3′-diaminobenzidine (DAB) substrate (QD440-XAKE, BioGenex). To co-stain microglia and Aβ protein, we performed double immunohistochemical staining; after deparaffinization and rehydration, the sections were treated with sodium citrate buffer (10 mM sodium citrate, pH 6.0) for 25 min and blocked, then endogenous peroxidase activity was quenched by 3% hydrogen peroxide solution. We then incubated the sections with the mouse anti-human Aβ antibody (SC-32277) and rabbit anti-human Iba-1 (1:100 dilution; 019-19741, polyclonal, FUJIFILM Wako Pure Chemical) overnight at 4 °C. After washing, we incubated the sections with HRP-labeled anti-mouse and AP-labeled anti-rabbit (HK597-50K, Double Staining kit, BioGenex) followed by substrate development with DAB (QD440-XAKE, BioGenex) and Fast Red Substrate (HK182-5KE, BioGenex). We then counterstained the sections with Mayer’s hematoxylin (HK100-9K, BioGenex) and mounted them with coverslips. We used Tris-buffered saline with 0.01% Triton X-100 as the buffer for washing and to dilute primary antibodies. We took images with a ZEISS Axio Scan.Z1 scanner and processed them with ZEN microscope software v3.2 (ZEISS).

To quantify Aβ plaques, we took ten random images of each section. After background subtraction and threshold adjustment, we analyzed the Aβ plaques using the Analyze Particles function in Fiji (ImageJ v1.53c). We determined the total Aβ area, number of Aβ plaques and median plaque size for each section. We calculated Aβ plaque load (percentage area) by dividing the total Aβ area by the total image area (10 mm^2^). To quantify microglia–Aβ co-staining, we selected 20 random images of each section and processed them with the Colour Deconvolution function to separate the data into three color channels (that is, DAB, Fast Red and hematoxylin). After adjusting the threshold, we selected Aβ plaques and microglia using the Create Selection function, then analyzed them using the Analyze function. We determined the total Aβ area and Aβ area colocalized with Iba-1 staining. We calculated the Aβ plaque area colocalized with microglia (percentage total Aβ) by dividing the Aβ area colocalized with Iba-1 staining by the total Aβ area. Two independent researchers performed section staining, image acquisition and image quantification; they also randomly selected and quantified images in a blinded manner.

### Association analysis and data visualization for the GWAS

We performed the association analysis between SNPs and plasma sST2 level at the genome-wide level in Chinese_cohort_1 with PLINK software (v1.9)^94^, adjusting for age, sex, AD diagnosis and the top five principal components as covariates (given that they have relatively larger eigenvalues with higher power in explaining the population variation in the initially calculated 20 principal components; Supplementary Data 3), using the following parameters: --keep-allele-order, --linear, --ci 0.95, --hwe 0.00001 and --maf 0.05. To visualize the data, we generated a Manhattan plot and quantile–quantile plot using the manhattan() function and qq() function of the R qqman package (v0.1.4), respectively. We generated regional plots for the *IL1RL1* locus using LocusZoom. We performed fine-mapping analysis of the effects of the *IL1RL1* locus on plasma sST2 level using CAVIAR software (v2.2)^95^ and generated association test results and pairwise LD information using PLINK software (v1.9) with the following parameters: --hwe 0.00001, --maf 0.05, --r, --matrix, --chr 2, --from-bp 102000000 and --to-bp 104000000. We generated the fine-mapped regional plot using the plot_ly() function of the R plotly package (v4.9.1) and plotted LD and haplotype structures using Haploview (v4.2). To identify all independent sST2-associated variants (*r*^*2*^ < 0.2), we subjected variants with *P* < 1 × 10^−5^ according to the sST2 GWAS to analysis by PLINK software (v1.9; parameters: --hwe 0.00001, --maf 0.05, --clump-p1 0.00001, --clump-r2 0.2, --chr 2 and --clump-kb 2000), yielding 29 independent sST2-associated variants (Supplementary Data 2). We used the calc.relimp() function of the R relaimpo package (v2.2-3)^96,97^ to quantify the contributions of genetic factors (that is, the 29 independent sST2-associated variants) and nongenetic factors (that is, age and sex) to sST2-level variance.

### Association analysis of rs1921622 in transcriptome datasets

We used human tissue sST2 and ST2L transcript levels as well as rs1921622 genotype data from the GTEx dataset^42,43^ for the genotype–expression association test, adjusting for age, sex, RNA integrity (that is, RNA integrity number) and population structure (that is, the top four principal components). We performed rank-based normalization of transcript levels using the rntransform() function of the R GenABEL package (v1.8).

We obtained the transcript levels of sST2 and ST2L in the human frontal cortex at the single-cell level by realigning the FASTQ files of our previously published snRNA-seq dataset^44^ using a modified reference genome. Specifically, we separated the *IL1RL1* region (chr2: 102,311,563–102,352,037) in the GTF file of the original GRCh38/hg38 pre-mRNA reference genome into three parts: the sST2-specific region (chr2: 102,343,416–102,346,100), the ST2L-specific region (chr2: 102,311,563–102,337,147 and 102,346,101–102,352,037) and the overlapping region (chr2: 102,337,148–102,343,415). We generated a modified reference genome with Cell Ranger (v3.0.1) using the new GTF file and original FASTA file. In the subsequent quality-control step, we performed the quantification of gene levels and cell-type identification using Seurat (v3.0)^44^. For the association analysis between genotype and candidate gene expression in each cell cluster, we performed a linear regression analysis, adjusting for age, sex, AD diagnosis and PMD. The level of significance was set at an FDR < 0.05. We performed a GO analysis of associated genes using DAVID Bioinformatics Resources^98,99^.

### Analysis of the association between rs1921622 and Alzheimer’s disease risk

We performed a meta-analysis to examine the effects of rs1921622 genotype on AD risk. Specifically, we determined the effect sizes (that is, log ORs) and standard errors from eight AD datasets (that is, the Chinese_cohort_1 dataset, the WGS and array datasets of Chinese_cohort_2 and the LOAD, ADC1, ADC2, ADC3 and ADNI datasets) using logistic regression with age, sex and the top five principal components as covariates. We summarized and processed the results by METASOFT (v2.0.0)^51^ to estimate the joint risk effects and significance levels under Han and Eskin’s random-effects model (RE2)^51^. We further performed multiple testing correction using the RE2 *P* values to generate the FDR values. We then input the results into ForestPMPlot (v1.0.2) to generate forest plots for data visualization.

### Two-sample Mendelian randomization analysis

We performed the two-sample MR analyses of the associations between sST2 level and AD risk in the overall population and subgroups of individuals in the Chinese and European-descent populations using the mr() function and inverse‐variance weighted method of the R TwoSampleMR package (v0.5.6), followed by multiple testing correction.

Accordingly, for the analysis in European-descent populations, we selected the *cis*-regulating (chr2: 102,820,000–103,100,000; GRCh37/hg19) protein quantitative trait loci (pQTL) for plasma sST2 levels^39^ for downstream filtering. Specifically, we used the statistical results from an independent plasma sST2 *cis*-regulating pQTL dataset from European-descent populations^38^ to filter out the loci that did not pass the genome-wide threshold (*P* < 5 × 10^−8^) or had the opposite direction of regulating effects in the two datasets. Moreover, we further used the statistical results from the GTEx dataset^42,43^ to filter the loci, and only kept the loci associated with the altered sST2 expression in the expressing tissues (that is, meta *P* < 1 × 10^−5^ in the brain regions and lungs) but not the altered ST2L expression in the expressing tissues (that is, *P* > 0.1 in the lungs) for LD clumping by PLINK software (v1.9) with the following parameters: --maf 0.05, --hwe 0.00001, --clump-p1 0.00000005, --clump-r2 0.2 and --clump-kb 2000. We obtained a total of seven independent instrumental variables (rs1921622, rs13001325, rs4851575, rs10515922, rs11123935, rs10200945 and rs951774), then generated their effects on AD risk by the meta-analysis in European-descent populations using the LOAD, ADC1, ADC2, ADC3 and ADNI datasets.

For the analysis in Chinese populations, we subjected the *cis*-regulating (chr2: 102,820,000–103,100,000; GRCh37/hg19) pQTL for plasma sST2 levels in the Chinese_cohort_1 (WGS) dataset to LD clumping by PLINK software (v1.9) with the following parameters: --maf 0.05, --hwe 0.00001, --clump-p1 0.00001, --clump-r2 0.5 and --clump-kb 2000. We obtained a total of seven independent instrumental variables (rs1921622, rs55664618, rs62151861, rs2241116, rs1468790, rs1523199 and rs56238602), then generated their effects on AD risk by the association analysis in the Chinese_cohort_2 (WGS) dataset.

### Mice

We housed all mice in the HKUST Animal and Plant Care Facility. All animal experiments were approved by the HKUST Animal Ethics Committee and conducted in accordance with the Guidelines of the Animal Care Facility of HKUST. We housed four mice of the same sex in each cage at 22 °C and at a relative humidity of 60%, with a 12-h light/dark cycle as well as food and water ad libitum. WT C57BL6J mice were obtained from the Jackson Laboratory. The 5XFAD mice were generated as previously described by overexpressing the p.Lys670Asn/p.Met671Leu (Swedish), p.Ile716Val (Florida) and p.Val717Ile (London) mutations in human *APP* as well as the p.Met146Leu and p.Leu286Val mutations in human *PSEN1* (ref. ^100^). We confirmed genotypes by PCR analysis of tail or ear biopsy specimens. We performed all in vivo experiments on age-matched groups and randomly assigned the mice to the experimental conditions. We chose our sample sizes primarily based on our experience with similar types of experiments. We conducted all animal experiments during the light phase.

### In vivo experiments in mice

We delivered murine recombinant sST2-Fc (1004-MR-050; R&D Systems) into 5XFAD mice (B6.Cg-Tg(APPSwFlLon,PSEN1*M146L*L286V)6799Vas/Mmjax) via mini-osmotic pumps (model 1004; Alzet) at 0.11 μl h^−1^. Specifically, we implanted the pumps intracerebroventricularly above the right hemisphere and loaded them with murine recombinant sST2-Fc protein (240 ng per pump; 10 μg ml^−1^) or human IgG (as a control; 009-000-008; Jackson ImmunoResearch) in artificial CSF (119 mM NaCl, 2.5 mM KCl, 2.5 mM CaCl_2_·2H_2_O, 1 mM NaH_2_PO_4_·2H_2_O, 1.3 mM MgCl_2_·6H_2_O, 26.2 mM NaHCO_3_ and 11 mM d-glucose). After 28 d of administration, the mice were anesthetized with isoflurane and transcardially perfused with PBS, and their brains were collected.

### Immunohistochemical and immunofluorescence staining of mouse brains

The left hemispheres of the mouse brains were fixed in 4% paraformaldehyde at 4 °C for 24 h, transferred to 30% sucrose and stored at 4 °C until sectioning. We cut the brains coronally into 50-μm sections with a vibrating blade microtome (VT1000S, Leica) and stored them in cryoprotectant solution (30% glycerol, 30% ethylene glycol and PBS) at −20 °C until use. For immunohistochemistry, we rinsed the sections with PBST (that is, 0.1% Triton X-100 in PBS), then treated them with formic acid at RT for 5 min for antigen retrieval, followed by 3% hydrogen peroxide solution for 30 min to quench endogenous peroxidase activity. We blocked the sections in 5% horse serum in PBST for 2 h, then labeled them with 4G8 antibody (1:1,000 dilution; 800703, BioLegend) or D54D2 antibody (1:1,000 dilution; 8243S, CST) in blocking buffer overnight at 4 °C. The next day, we incubated the sections with biotin-conjugated anti-mouse secondary antibodies (1:1,000 dilution; BA2000, Vector Laboratories) followed by an avidin–biotin–HRP complex (PK-6100, Vector Laboratories), and we developed signals with DAB kits (SK-4100, Vector Laboratories or QD430-XAKE, BioGenex). Imaging was performed using a Zeiss Axio Scan.Z1 Slide Scanner and processed in ZEN v3.3 (ZEISS). We analyzed the Aβ plaque areas in cortical sections using the Analyze Particles function in Fiji (ImageJ v1.53c). Specifically, we analyzed four brain sections from each mouse in cortical regions (~200–300 μm apart) and calculated the average percentage of the cortical area occupied by Aβ plaques.

For immunofluorescence analysis, we washed sections and incubated them in 1 μM X-34 for 10 min. We then washed them in X-34 buffer (40% ethanol in PBS), and then in PBS. We then blocked sections for 2 h in blocking buffer (4% horse serum, 1% BSA and 0.3% Triton X-100 in PBS). Primary antibodies used in experiments include mouse anti-Aβ (1:1,000 dilution; clone 4G8, 800703, BioLegend), rabbit anti-Iba-1 (1:1,000 dilution; 019-19741, Wako) and rat anti-Ki67 (1:200 dilution; clone SolA15, 14-5698-80, eBioscience); we diluted these in blocking buffer and incubated sections overnight at 4 °C. Sections were subsequently incubated with fluorophore-conjugated secondary antibodies against mouse, rabbit and rat Ig (Alexa Fluor 488, 568 and 647; 1:1,000 dilution; Life Technologies) in blocking buffer for 2 h at RT, extensively washed in PBST, stained with SYTOX Green (1:300,000 dilution; S7020, Life Technologies) or DAPI (1:5,000 dilution; D3571, Life Technologies) and mounted using FluorSave Reagent (345789, Millipore).

We performed imaging using a Leica TCS SP8 confocal microscope with a Leica ×40 oil immersion objective. We took five images from each mouse cortex with a step size of 1 μm for a total of 40 μm, then merged them into a single image with maximum intensity *z*-projection. We identified three different plaque morphologies using anti-Aβ immunolabeling (that is, 4G8) and X-34 staining: (i) filamentous plaques characterized by filamentous X-34 and 4G8 labeling with no plaque core, (ii) compact plaques characterized by 4G8 amyloid fibrils projecting radially outward with an X-34–labeled core and (iii) inert plaques characterized by an X-34-labeled core with no 4G8 labeling. We used a custom macro to segment individual Aβ plaques and identified the type of each plaque manually. For plaque-associated microglia, we counted the numbers of microglia surrounding small plaques (that is, radius ≤ 8 μm) and large plaques (that is, radius > 8 μm) manually, as defined by DAPI^+^ nucleus staining within the barrier surrounding the plaques and processes in contact with the plaques. We defined the Ki67^+^ microglia as microglia with Ki67 signals within the nucleus. To quantify microglial coverage of Aβ plaques, we selected only compact plaques. We used ten optical slices 0.5 μm apart through the center of the plaque for analysis. We processed all images with a customized macro in Fiji (ImageJ v1.53c). On each slice, after adjusting the threshold, we determined the perimeters of the plaque using the Analyse Particles and Area to Line functions. We also determined plaque perimeters and the arcs of plaque perimeters colocalized with microglial staining. We calculated the proportion of the plaque perimeter covered by microglia by summing the arcs of the plaque perimeter across three-dimensional (3D) stacks in close contact (within 1 μm) with Iba-1-immunolabeled cells (~25 plaques per group). We conducted the 3D reconstruction of microglia–plaque interaction using Imaris v9.7.2 (Oxford Instruments).

### Assessment of microglial amyloid-beta phagocytic capacity

We examined microglial Aβ phagocytic capacity as previously described^25^. Briefly, we injected 4-month-old 5XFAD or WT mice intraperitoneally with methoxy-X04 (10 mg per kg body weight) to label the Aβ. We anesthetized the mice with isoflurane 3 h after methoxy-X04 injection and perfused the left ventricle with ice-cold PBS. We isolated, minced and incubated their forebrains at 37 °C for 30 min in 5 U ml^−1^ papain (LS003126) and 35 U ml^−1^ DNase I (LS002140; Worthington Biochemical) for enzymatic digestion. After incubation, we depleted myelin debris by 30% isotonic Percoll (P1644; Sigma-Aldrich) gradient centrifugation and obtained mononuclear cell suspensions in DMEM/F12 medium with ice-cold 10% heat-inactivated fetal bovine serum. We prepared unstained controls from mixtures of different sample cell suspensions for cell population identification. To label microglia, we used an Alexa Fluor 488-conjugated mouse CD11b antibody (1:200 dilution; 53-0112-82; eBioscience) to stain the cell suspensions for 45 min at 4 °C. We analyzed the resultant labeled cell suspensions using a BD Influx cell sorter flow cytometer. We analyzed the recorded scatterplot data for the microglial cell population using FlowJo software v10.5.0 (TreeStar).

### Cell culture

The hCMEC/D3 cell line was purchased from Cedarlane and cultured as previously described^101^. Briefly, we coated a tissue culture plate with 100 μg ml^−1^ type I collagen (Millipore) at 37 °C in 5% CO_2_ for 1 h. We subsequently washed the plate with Dulbecco’s phosphate-buffered saline (DPBS) and replaced it with a complete culture medium (endothelial cell growth medium-2 (Lonza) supplemented with 5% fetal bovine serum (HyClone), 1% chemically defined lipid concentrate (Gibco), 10 mM HEPES (Gibco), 5 μg ml^−1^ ascorbic acid (Sigma), 1.4 μM hydrocortisone (Sigma), 1 ng ml^−1^ basic fibroblast growth factor bFGF (PeproTech), 10 U ml^−1^ penicillin and 10 μg ml^−1^ streptomycin (Gibco)). Cultured cells were dissociated with 0.05% trypsin for 5 min, replated at 25,000 cells per cm^2^ and returned to culture at 37 °C in a 5% CO_2_ incubator. Three to four days after seeding, the cells reached confluence and could be passaged. We used cells at passages 27–35 for our experiments.

For the ChIP–qPCR experiment, we fully changed the medium of the hCMEC/D3 cells 2 h before treatment. We then treated cultured cells with recombinant human IL-33 (BioLegend) or DPBS as a vehicle control for 24 h.

To evaluate the efficiency of the sgRNA editing in endothelial cells, we transfected 5 × 10^5^ hCMEC/D3 cells with a single CRISPR construct by nucleofection using the Human Umbilical Vein Endothelial Cell Nucleofector Kit (Lonza) with a Nucleofector 2b device (Lonza). One day after transfection, we changed the cultured medium to a complete culture medium with 1 μg ml^−1^ puromycin (Thermo Fisher Scientific). After 3 d of puromycin selection, we extracted genomic DNA using QuickExtract DNA Extraction Solution (Lucigen) followed by a T7EI (NEB) editing efficiency test. All four sgRNAs exhibited high editing efficiency (data not shown).

### CRISPR/Cas9-mediated genome deletion of the rs1921622-harboring region

To delete the region harboring rs1921622 in hCMEC/D3 cells, we used a dual-guide, RNA-mediated knockout approach. The cells were genotyped from 300 bp upstream and downstream of rs1921622 by Sanger sequencing. We performed screening of potential *Streptococcus pyogenes* Cas9 (SpCas9)-guided RNAs using the CRISPR design tool (https://crispr.mit.edu/) 100 bp upstream and downstream of rs1921622. We used the following sgRNAs: sgRNA-1, 5′-TTATGGACAGAATTAAGAAG-3′; sgRNA-2, 5′-CTGTCCATAAGATTTGAAAG-3′; sgRNA-3, 5′-AATTTTGTTCTGGTAGCCAT-3′; and sgRNA-4, 5′-GGTATTTCAGCTAGTGCCTA-3′. We subcloned the sgRNAs into PX459v2, which contains an sgRNA cassette, human codon-optimized SpCas9 and a puromycin resistance gene.

To generate a dual-gRNA-mediated deletion cell line, we transfected the hCMEC/D3 cells with plasmids containing sgRNA-1/sgRNA-4 (targeted 67-bp deletion), sgRNA-2/sgRNA-3 (targeted 38-bp deletion) or PX459v2 as a no-sgRNA control. After 3 d of puromycin selection (1 μg ml^−1^) starting from the day after transfection, we seeded the puromycin-resistant cells in two 24-well plates. We changed the culture medium twice a week. After 3 weeks, when a single colony was observed, we passaged the wells containing only one colony into a 12-well plate. We genotyped each clone and subjected those in which the targeted deletion may have occurred to Sanger sequencing. This protocol generated six control lines, eight lines with a 38-bp deletion and ten lines with a 67-bp deletion.

### RNA extraction and real-time PCR

We extracted RNA using a NucleoSpin RNA column (MACHEREY-NAGEL) according to the manufacturer’s instructions. We first synthesized single-stranded cDNA using the PrimeScript RT Reagent Kit (Takara Bio). We performed quantitative real-time PCR with TaqMan probes (Thermo Fisher Scientific) and a Premix Ex Taq Kit (Takara Bio) using the 7500 Fast Real-Time PCR System (Applied Biosystems). The mRNA expression was normalized to that of *HPRT1*.

### Chromatin immunoprecipitation coupled to quantitative PCR

We modified the ChIP–qPCR from the chromatin immunoprecipitation sequencing protocol as previously described^25^. In brief, we trypsinized cultured cells and washed them once with DPBS. Then, we immediately fixed 4 × 10^5^ cells in suspension with 1.5% paraformaldehyde at RT for 10 min and quenched them with 0.125 M glycine for 5 min. We subsequently centrifuged the cells at 500*g* for 5 min. We resuspended the cell pellet in 0.25% SDS sonication buffer (10 mM Tris (pH 8.0), 0.25% SDS, 2 mM EDTA and protease inhibitors) and subjected it to sonication into 200–500-bp fragments using the S220 Focused-ultrasonicator system (Covaris). We diluted the DNA–protein mixture at a 1:1.5 ratio in equilibration buffer (10 mM Tris, 233 mM NaCl, 1.67% Triton X-100, 0.167% sodium-deoxycholate (Na-DOC), 1 mM EDTA and protease inhibitors). We spared 5% of the DNA–protein mixture as input, while we incubated the remainder with antibodies against H3K4me3 (3 μl; 39159; Active Motif), H3K27ac (5 μl; 39133; Active Motif) or normal IgG (6 μl; AB-105-C; R&D) overnight at 4 °C. Simultaneously, Protein A Dynabeads (Thermo Fisher Scientific) were washed twice and incubated with RIPA-LS buffer (10 mM Tris-HCl (pH 8.0), 140 mM NaCl, 1 mM EDTA (pH 8.0), 0.1% SDS, 0.1% Na-DOC, 1% Triton X-100 and protease inhibitors), then supplemented with 0.1% BSA overnight at 4 °C. The next day, we added 10 μl pre-blocked beads to each sample and incubated them for 2 h at 4 °C. We then washed protein-bound beads twice with ice-cold RIPA-LS buffer, twice with ice-cold RIPA-HS buffer (10 mM Tris-HCl (pH 8.0), 500 mM NaCl, 1 mM EDTA (pH 8.0), 0.1% SDS, 0.1% Na-DOC, 1% Triton X-100 and protease inhibitors), twice with ice-cold RIPA-LiCl buffer (10 mM Tris-HCl (pH 8.0), 250 mM LiCl, 1 mM EDTA (pH 8.0), 0.5% NP-40, 0.5% Na-DOC and protease inhibitors) and twice with ice-cold 10 mM Tris (pH 8.0) buffer on a precooled magnetic stand. We then reverse-crosslinked the protein-bound and chromatin-bound beads with protease K (NEB) in ChIP elution buffer (10 mM Tris-HCl (pH 8.0), 300 mM NaCl, 5 mM EDTA (pH 8.0) and 10% SDS) at 55 °C for 1 h, then at 65 °C for 6 h. We diluted the input to a final concentration of 0.4% SDS and 300 mM NaCl. Next, 2 μl protease K was added to the input and incubated at 55 °C for 1 h, then at 65 °C for 6 h. We purified both the eluted ChIP and input DNA with a MinElute column (Qiagen).

We performed real-time PCR for ChIP and input DNA using Power SYBR Green Master Mix (Applied Biosystems) with the following primer pairs: sST2_promoter_F, 5′-GGGAAAAAAGCTTGACTTTG-3′; sST2_promoter_R, 5′-ATTTGACAAAGTTCACCCAG-3′; rs192_F, 5′-GCCACTTCTTAATTCTGTCC-3′; and rs192_R, 5′-GGTATTTCAGCTAGTGCCTA-3′.

### Statistics and reproducibility

No statistical methods were used to predetermine sample sizes, but our samples sizes are similar to those reported in previous publications^24,25,41,47^. No data were excluded from the analyses, and the data distribution was assumed to be normal, but this was not formally tested. We assigned random codes to human samples before experiments and analyses; and we randomly assigned nonhuman samples (for example, mice and cultured cells) into experimental groups before experiments and sample collection. The investigators were blinded to disease diagnosis and other demographic information (for humans) and experimental groups and conditions (for mice and cultured cells) during experiments.

For the remaining statistical analyses of human samples that are not mentioned in the above methods, we conducted a linear regression analysis to determine the significance of the associations between AD-associated endophenotypes (that is, cognitive performance, brain region volume and AD biomarkers) and sST2 level and/or rs1921622 genotype, adjusted for age, sex and other covariates as indicated in the main text and figure legends. We obtained the CSF sST2 level cutoff based on the CSF sST2 level with the maximum value of Youden’s index using the optimal.cutpoints() function and the Youden method of the OptimalCutpoints package (v1.1-4) in R^102^. We performed Cox regression to examine the association between onset age of dementia and the rs1921622 A allele using the coxph() function of the R survival package (v1.3-24), with sex and the top five principal components as covariates. We tested the proportional hazard assumptions using the cox.zph() function of the R survival package (v1.3-24; Supplementary Data 4). The level of significance was set to *P* < 0.05 or FDR < 0.05. For data visualization, we used the plot() function of R to generate a volcano plot and the ggplot() function of the R ggplot2 package (v3.2.1) to generate dot plots. For the data obtained from mouse and cell culture system experiments, we assessed the significance of differences by unpaired Student’s *t*-tests, or one-way or two-way analysis of variance followed by the Bonferroni post hoc test as indicated. The level of significance was set at *P* < 0.05. We generated all statistical plots using Prism v8.0 (GraphPad).

### Reporting summary

Further information on research design is available in the Nature Research Reporting Summary linked to this article.

## Supplementary information

Supplementary Information Supplementary Notes 1–9, Figs. 1–8 and Tables 1–9

Reporting Summary

Supplementary Data 1 Supplementary Data 1–4.

Supplementary Video 1 3D reconstruction for Fig. 7f.

## Data Availability

Source data are available with this paper. All statistical data associated with this study are contained in the main text, Supplementary Information or Supplementary Data. All other data are available from the corresponding author upon reasonable request. The consent forms signed by individuals from Chinese_cohort_1 state that the research content will be kept private under the supervision of the hospital and research team. Therefore, the phenotypic, genomic and proteomic data of individuals will only be available and shared in formal collaborations. A review panel hosted at HKUST will process and review any applications for data sharing and project collaboration and promptly notify applicants with the decision. Researchers may contact HKUST (sklneurosci@ust.hk) for details about data sharing and project collaboration related to the present study. The GRCh38/hg38 reference genome is available at https://hgdownload.soe.ucsc.edu/downloads.html. The human frontal cortex snRNA-seq dataset of the UKBBN cohort has been deposited in the Gene Expression Omnibus (accession no. GSE157827). The genomic, demographic and clinical data of the LOAD cohort are available on the National Institutes of Health (NIH) database of Genotypes and Phenotypes (dbGaP) project (accession no. phs000168.v2.p2). The genomic, demographic and clinical data of the ADC1–3 cohorts are available on the NIH dbGaP project database (accession no. phs000372.v2.p1). The genomic, demographic, clinical and brain imaging data of the ADNI cohort are available at http://adni.loni.usc.edu/ upon request. The proteomic and demographic data from the INTERVAL and LonGenity cohorts are available at https://twc-stanford.shinyapps.io/aging_plasma_proteome/. The genomic, demographic and transcriptomic data from the GTEx cohort are available on the NIH dbGaP project database (accession no. phs000424.v6.p1). The genomic, demographic, clinical and brain imaging data of the AIBL cohort are available at https://aibl.csiro.au/ upon request.

## Source data

Source Data Fig. 2 Statistical source data for Fig. 2a.
Source Data Fig. 6 Statistical source data for Fig. 6e–g.

## Code availability

The codes for our statistical analyses and data visualization are available at https://github.com/yjiangah/sST2-in-AD/.

## References

[1] Alzheimer’s Association (2019). Alzheimer’s disease facts and figures. Alzheimers Dement..

[2] Jack CR (2018). NIA‐AA research framework: toward a biological definition of Alzheimer’s disease. Alzheimers Dement..

[3] Lambert J-C (2013). Meta-analysis of 74,046 individuals identifies 11 new susceptibility loci for Alzheimer’s disease. Nat. Genet..

[4] Frigerio CS (2019). The major risk factors for Alzheimer’s disease: age, sex and genes modulate the microglia response to Aβ plaques. Cell Rep..

[5] Yeh FL, Wang Y, Tom I, Gonzalez LC, Sheng M (2016). TREM2 binds to apolipoproteins, including APOE and CLU/APOJ, and thereby facilitates uptake of amyloid-beta by microglia. Neuron.

[6] Rebeck GW, Reiter JS, Strickland DK, Hyman BT (1993). Apolipoprotein E in sporadic Alzheimer’s disease: allelic variation and receptor interactions. Neuron.

[7] Kok E (2009). Apolipoprotein E–dependent accumulation of Alzheimer disease–related lesions begins in middle age. Ann. Neurol..

[8] Castellano JM (2011). Human apoE isoforms differentially regulate brain amyloid-β peptide clearance. Sci. Transl. Med..

[9] Stephen, T. L. et al. APOE genotype and sex affect microglial interactions with plaques in Alzheimer’s disease mice. *Acta Neuropathol. Commun*. **7**, 82 (2019). 10.1186/s40478-019-0729-z PMC6528326 31113487

[10] Hickman SE, Allison EK, El Khoury J (2008). Microglial dysfunction and defective β-amyloid clearance pathways in aging Alzheimer’s disease mice. J. Neurosci..

[11] Guillot-Sestier M-V (2015). Il10 deficiency rebalances innate immunity to mitigate Alzheimer-like pathology. Neuron.

[12] Suárez-Calvet M (2016). Early changes in CSF sTREM2 in dominantly inherited Alzheimer’s disease occur after amyloid deposition and neuronal injury. Sci. Transl. Med..

[13] Ewers, M. et al. Increased soluble TREM2 in cerebrospinal fluid is associated with reduced cognitive and clinical decline in Alzheimer’s disease. *Sci. Transl. Med.* **11**, eaav6221 (2019). 10.1126/scitranslmed.aav6221 PMC7050285 31462511

[14] Wood H (2017). Soluble TREM2 in CSF sheds light on microglial activation in AD. Nat. Rev. Neurol..

[15] Zhong L (2017). Soluble TREM2 induces inflammatory responses and enhances microglial survival. J. Exp. Med..

[16] Zhong L (2019). Soluble TREM2 ameliorates pathological phenotypes by modulating microglial functions in an Alzheimer’s disease model. Nat. Commun..

[17] Janelidze S (2018). CSF biomarkers of neuroinflammation and cerebrovascular dysfunction in early Alzheimer disease. Neurology.

[18] Huang C-W (2015). Clinical significance of circulating vascular cell adhesion molecule-1 to white matter disintegrity in Alzheimer’s dementia. Thromb. Haemost..

[19] Yousef H (2019). Aged blood impairs hippocampal neural precursor activity and activates microglia via brain endothelial cell VCAM1. Nat. Med..

[20] Chakrabarty P (2018). TLR5 decoy receptor as a novel anti-amyloid therapeutic for Alzheimer’s disease. J. Exp. Med..

[21] Liu, Y. -L. et al. Amelioration of amyloid-β-induced deficits by DcR3 in an Alzheimer’s disease model. *Mol. Neurodegener*. **12**, 30 (2017). 10.1186/s13024-017-0173-0 PMC5402663 28438208

[22] Griesenauer B, Paczesny S (2017). The ST2/IL-33 axis in immune cells during inflammatory diseases. Front. Immunol..

[23] Yasuoka S (2011). Production and functions of IL-33 in the central nervous system. Brain Res..

[24] Fu AK (2016). IL-33 ameliorates Alzheimer’s disease-like pathology and cognitive decline. Proc. Natl Acad. Sci. USA.

[25] Lau S-F (2020). IL-33-PU.1 transcriptome reprogramming drives functional state transition and clearance activity of microglia in Alzheimer’s disease. Cell Rep..

[26] Trajkovic V, Sweet MJ, Xu D (2004). T1/ST2—an IL-1 receptor-like modulator of immune responses. Cytokine Growth Factor Rev..

[27] Kakkar R, Lee RT (2008). The IL-33/ST2 pathway: therapeutic target and novel biomarker. Nat. Rev. Drug Discov..

[28] Homsak, E. & Gruson, D. Soluble ST2: a complex and diverse role in several diseases. *Clin. Chim. Acta* **507**, 75–87 (2020). 10.1016/j.cca.2020.04.011 32305537

[29] Oshikawa K (2001). Elevated soluble ST2 protein levels in sera of patients with asthma with an acute exacerbation. Am. J. Respir. Crit. Care Med..

[30] Bergis D, Kassis V, Radeke HH (2016). High plasma sST2 levels in gastric cancer and their association with metastatic disease. Cancer Biomark..

[31] Villacorta H, Maisel AS (2016). Soluble ST2 testing: a promising biomarker in the management of heart failure. Arq. Bras. Cardiol..

[32] Saresella M (2020). IL-33 and its decoy sST2 in patients with Alzheimer’s disease and mild cognitive impairment. J. Neuroinflammation.

[33] Karikari TK (2020). Blood phosphorylated tau 181 as a biomarker for Alzheimer’s disease: a diagnostic performance and prediction modelling study using data from four prospective cohorts. Lancet Neurol..

[34] Preische O (2019). Serum neurofilament dynamics predicts neurodegeneration and clinical progression in presymptomatic Alzheimer’s disease. Nat. Med..

[35] Gate D (2020). Clonally expanded CD8 T cells patrol the cerebrospinal fluid in Alzheimer’s disease. Nature.

[36] Sasayama D (2013). Increased cerebrospinal fluid interleukin-6 levels in patients with schizophrenia and those with major depressive disorder. J. Psychiatric Res..

[37] Lehallier B (2019). Undulating changes in human plasma proteome profiles across the lifespan. Nat. Med..

[38] Ho JE (2013). Common genetic variation at the IL1RL1 locus regulates IL-33/ST2 signaling. J. Clin. Invest..

[39] Sun BB (2018). Genomic atlas of the human plasma proteome. Nature.

[40] Grotenboer NS, Ketelaar ME, Koppelman GH, Nawijn MC (2013). Decoding asthma: translating genetic variation in IL33 and IL1RL1 into disease pathophysiology. J. Allergy Clin. Immunol..

[41] Zhou X (2019). Non-coding variability at the APOE locus contributes to the Alzheimer’s risk. Nat. Commun..

[42] Lonsdale J (2013). The Genotype-Tissue Expression project. Nat. Genet..

[43] Consortium G (2015). The Genotype-Tissue Expression pilot analysis: multitissue gene regulation in humans. Science.

[44] Lau S-F, Cao H, Fu AK, Ip NY (2020). Single-nucleus transcriptome analysis reveals dysregulation of angiogenic endothelial cells and neuroprotective glia in Alzheimer’s disease. Proc. Natl Acad. Sci. USA.

[45] Li W, Notani D, Rosenfeld MG (2016). Enhancers as non-coding RNA transcription units: recent insights and future perspectives. Nat. Rev. Genet..

[46] Davies, N. M., Holmes, M. V. & Smith, G. D. Reading Mendelian randomisation studies: a guide, glossary and checklist for clinicians. *BMJ* **362**, k601 (2018). 10.1136/bmj.k601 PMC6041728 30002074

[47] Zhou X (2018). Identification of genetic risk factors in the Chinese population implicates a role of immune system in Alzheimer’s disease pathogenesis. Proc. Natl Acad. Sci. USA.

[48] Lee JH, Cheng R, Graff-Radford N, Foroud T, Mayeux R (2008). Analyses of the National Institute on Aging late-onset alzheimer’s disease family study: implication of additional loci. Arch. Neurol..

[49] Naj AC (2011). Common variants at MS4A4/MS4A6E, CD2AP, CD33 and EPHA1 are associated with late-onset Alzheimer’s disease. Nat. Genet..

[50] Jun G (2010). Meta-analysis confirms CR1, CLU and PICALM as alzheimer disease risk loci and reveals interactions with APOE genotypes. Arch. Neurol..

[51] Han B, Eskin E (2011). Random-effects model aimed at discovering associations in meta-analysis of genome-wide association studies. Am. J. Hum. Genet..

[52] Kathryn, A. E. et al. The Australian Imaging, Biomarkers and Lifestyle (AIBL) study of aging: methodology and baseline characteristics of 1,112 individuals recruited for a longitudinal study of Alzheimer’s disease. *Int. Psychogeriatr.* **21**, 672–687 (2009). 10.1017/S1041610209009405 19470201

[53] Mathys H (2019). Single-cell transcriptomic analysis of Alzheimer’s disease. Nature.

[54] Zhou Y (2020). Human and mouse single-nucleus transcriptomics reveal TREM2-dependent and TREM2-independent cellular responses in Alzheimer’s disease. Nat. Med..

[55] Keren-Shaul H (2017). A unique microglia type associated with restricting development of Alzheimer’s disease. Cell.

[56] Thrupp N (2020). Single-nucleus RNA-seq is not suitable for detection of microglial activation genes in humans. Cell Rep..

[57] Yuan P (2016). TREM2 haplodeficiency in mice and humans impairs the microglia barrier function leading to decreased amyloid compaction and severe axonal dystrophy. Neuron.

[58] Wicher G (2017). Interleukin-33 promotes recruitment of microglia/macrophages in response to traumatic brain injury. J. Neurotrauma.

[59] Zhang Y (2014). An RNA-sequencing transcriptome and splicing database of glia, neurons and vascular cells of the cerebral cortex. J. Neurosci..

[60] Gosselin, D. et al. An environment-dependent transcriptional network specifies human microglia identity. *Science* **356**, eaal3222 (2017). 10.1126/science.aal3222 PMC5858585 28546318

[61] Wang, Y. et al. Astrocyte-secreted IL-33 mediates homeostatic synaptic plasticity in the adult hippocampus. *Proc. Natl Acad. Sci. USA* **118**, e2020810118 (2021). 10.1073/pnas.2020810118 PMC7817131 33443211

[62] Nguyen PT (2020). Microglial remodeling of the extracellular matrix promotes synapse plasticity. Cell.

[63] Zhang Y (2016). Purification and characterization of progenitor and mature human astrocytes reveals transcriptional and functional differences with mouse. Neuron.

[64] Kalari KR (2016). BBBomics—human blood–brain barrier transcriptomics hub. Front. Neurosci..

[65] Demyanets S (2013). Components of the interleukin-33/ST2 system are differentially expressed and regulated in human cardiac cells and in cells of the cardiac vasculature. J. Mol. Cellular Cardiol..

[66] Joshi AD (2010). Interleukin-33 contributes to both M1 and M2 chemokine marker expression in human macrophages. BMC Immunol..

[67] Bandara G, Beaven MA, Olivera A, Gilfillan AM, Metcalfe DD (2015). Activated mast cells synthesize and release soluble ST2‐a decoy receptor for IL‐33. Eur. J. Immunol..

[68] Zhang J (2015). ST2 blockade reduces sST2-producing T cells while maintaining protective mST2-expressing T cells during graft-versus-host disease. Sci. Transl. Med..

[69] Lipsky BP, Toy DY, Swart DA, Smithgall MD, Smith D (2012). Deletion of the ST2 proximal promoter disrupts fibroblast‐specific expression but does not reduce the amount of soluble ST2 in circulation. Eur. J. Immunol..

[70] Kikuchi, M. et al. Altered behavior in mice overexpressing soluble ST2. *Mol. Brain* **13**, 74 (2020). 10.1186/s13041-020-00606-4 PMC7216579 32393354

[71] Zlokovic BV (2011). Neurovascular pathways to neurodegeneration in Alzheimer’s disease and other disorders. Nat. Rev. Neurosci..

[72] Hilscher MB (2019). Mechanical stretch increases expression of CXCL1 in liver sinusoidal endothelial cells to recruit neutrophils, generate sinusoidal microthombi and promote portal hypertension. Gastroenterology.

[73] Grammas P, Ovase R (2001). Inflammatory factors are elevated in brain microvessels in Alzheimer’s disease. Neurobiol. Aging.

[74] Grammas P, Ovase R (2002). Cerebrovascular transforming growth factor-β contributes to inflammation in the Alzheimer’s disease brain. Am. J. Pathol..

[75] Ferretti MT (2018). Sex differences in Alzheimer disease—the gateway to precision medicine. Nat. Rev. Neurol..

[76] Laws KR, Irvine K, Gale TM (2018). Sex differences in Alzheimer’s disease. Curr. Opin. Psych..

[77] Laws KR, Irvine K, Gale TM (2016). Sex differences in cognitive impairment in Alzheimer’s disease. World J. Psychiatry.

[78] Guillot-Sestier, M. -V. et al. Microglial metabolism is a pivotal factor in sexual dimorphism in Alzheimer’s disease. *Commun. Biol*. **4**, 711 (2021). 10.1038/s42003-021-02259-y PMC8192523 34112929

[79] Russi AE, Ebel ME, Yang Y, Brown MA (2018). Male-specific IL-33 expression regulates sex-dimorphic EAE susceptibility. Proc. Natl Acad. Sci. USA.

[80] Fernandez CG, Hamby ME, McReynolds ML, Ray WJ (2019). The role of APOE4 in disrupting the homeostatic functions of astrocytes and microglia in aging and Alzheimer’s disease. Front. Aging Neurosci..

[81] Fourgeaud L (2016). TAM receptors regulate multiple features of microglial physiology. Nature.

[82] Kim J, Basak JM, Holtzman DM (2009). The role of apolipoprotein E in Alzheimer’s disease. Neuron.

[83] Wang Y (2015). TREM2 lipid sensing sustains the microglial response in an Alzheimer’s disease model. Cell.

[84] Mittal K (2019). CD4 T cells induce a subset of MHCII-expressing microglia that attenuates Alzheimer pathology. iScience.

[85] Miller AM (2008). IL-33 reduces the development of atherosclerosis. J. Exp. Med..

[86] Baba Y (2012). Involvement of PU.1 in mast cell/basophil-specific function of the human IL1RL1/ST2 promoter. Allergol. Int..

[87] Kunkle BW (2019). Genetic meta-analysis of diagnosed Alzheimer’s disease identifies new risk loci and implicates Aβ, tau, immunity and lipid processing. Nat. Genet..

[88] Jun G (2016). A novel Alzheimer disease locus located near the gene encoding tau protein. Mol. Psychiatry.

[89] O’Meara E (2018). Independent prognostic value of serum soluble ST2 measurements in patients with heart failure and a reduced ejection fraction in the PARADIGM-HF trial (prospective comparison of ARNI with ACEI to determine impact on global mortality and morbidity in heart failure). Circ. Heart Fail..

[90] American Psychiatric Association. *Diagnostic and Statistical Manual of Mental Disorders* 5th edn. (American Psychiatric Association, 2013).

[91] Pangman VC, Sloan J, Guse L (2000). An examination of psychometric properties of the Mini-Mental State Examination and the standardized mini-mental state examination: implications for clinical practice. Appl. Nurs. Res..

[92] Nasreddine ZS (2005). The Montreal Cognitive Assessment, MoCA: a brief screening tool for mild cognitive impairment. J. Am. Geriatr. Soc..

[93] Jiang, Y. et al. Large‐scale plasma proteomic profiling identifies a high‐performance biomarker panel for Alzheimer’s disease screening and staging. *Alzheimers Dement.* **18**, 88–102 (2021). 10.1002/alz.12369 PMC9292367 34032364

[94] Purcell S (2007). PLINK: a tool set for whole-genome association and population-based linkage analyses. Am. J. Hum. Genet..

[95] Hormozdiari F, Kostem E, Kang EY, Pasaniuc B, Eskin E (2014). Identifying causal variants at loci with multiple signals of association. Genetics.

[96] Grömping U (2006). Relative importance for linear regression in R: the package relaimpo. J. Stat. Softw..

[97] Patin E (2018). Natural variation in the parameters of innate immune cells is preferentially driven by genetic factors. Nat. Immunol..

[98] Huang DW, Sherman BT, Lempicki RA (2009). Bioinformatics enrichment tools: paths toward the comprehensive functional analysis of large gene lists. Nucleic Acids Res..

[99] Sherman BT, Lempicki RA (2009). Systematic and integrative analysis of large gene lists using DAVID bioinformatics resources. Nat. Protoc..

[100] Oakley H (2006). Intraneuronal β-amyloid aggregates, neurodegeneration, and neuron loss in transgenic mice with five familial Alzheimer’s disease mutations: potential factors in amyloid plaque formation. J. Neurosci..

[101] Weksler B (2005). Blood–brain barrier‐specific properties of a human adult brain endothelial cell line. FASEB J..

[102] Youden WJ (1950). Index for rating diagnostic tests. Cancer.

