# Supplementary material for: An IL1RL1 genetic variant lowers soluble ST2 levels and the risk effects of APOE-ε4 in female patients with Alzheimer’s disease
Source: Nat Aging. 2022 Jul 15;2(7):616–34. doi: 10.1038/s43587-022-00241-9 (PMC10154240; doi:10.1038/s43587-022-00241-9)
Supplement: Supplementary file 1 — Supplementary Notes 1–9, Figs. 1–8 and Tables 1–9 [file 43587_2022_241_MOESM1_ESM.pdf]

nature portfolio
https://doi.org/10.1038/s43587-022-00241-9

Supplementary information

An *IL1RL1* genetic variant lowers soluble ST2 levels and the risk effects of *APOE*-ε4 in female patients with Alzheimer's disease

In the format provided by the authors and unedited

Supplementary Information

Table of Contents

1. Supplementary Notes

1. Late Onset Alzheimer's Disease Family Study
2. National Institute on Aging Alzheimer's Disease Centers cohorts
3. Alzheimer's Disease Neuroimaging Initiative cohort
4. Australian Imaging, Biomarkers and Lifestyle Study cohort
5. Genotype-Tissue Expression project dataset
6. Funding information of ADNI, LOAD, ADC and GTEx datasets
7. Investigators of the ADNI I, GO, II, and III studies
8. Investigators of ADNI from university and institutes
9. Biomarkers Consortium Project team members of ADNI

2. Supplementary Figures

1. Supplementary Fig. 1. Associations between cerebrospinal fluid soluble ST2 levels and number and size of amyloid-beta plaques in patients with Alzheimer's disease in the UK Brain Banks Network cohort.
2. Supplementary Fig. 2. Fine-mapping analysis of soluble ST2-associated genetic variants in the *IL1RL1* gene.
3. Supplementary Fig. 3. Interleukin 33 induces the expression and secretion of soluble ST2 in brain endothelial cells.
4. Supplementary Fig. 4. Validation of the CRISPR/Cas9-based target deletion at the rs1921622-containing region in hCMEC/D3 cells.
5. Supplementary Fig. 5. Representative images of filamentous, compact, and inert amyloid-beta plaques in 5XFAD mice.
6. Supplementary Fig. 6. Elevated brain soluble ST2 level leads to increased number of microglia in female 5XFAD mice.
7. Supplementary Fig. 7. Representative images showing the co-staining of microglia and amyloid-beta plaques in 5XFAD mice.
8. Supplementary Fig. 8. The gating strategy for amyloid-beta $(A\beta)^+$ microglia.

3. Supplementary Tables

1. Supplementary Table 1. Demographic characteristics of the Chinese_cohort_1.
2. Supplementary Table 2. Associations between plasma sST2 levels and Alzheimer's disease and its related endophenotypes in the Chinese_cohort_1.

3. Supplementary Table 3. Candidate genetic variants in the *IL1RL1* gene associated with plasma soluble ST2 level after fine mapping (causal probability >0.001).
4. Supplementary Table 4. Associations between the rs1921622 A allele and soluble ST2 and full-length ST2 transcript levels in human tissues.
5. Supplementary Table 5. Demographic characteristics of the seven Alzheimer's disease datasets for meta-analysis.
6. Supplementary Table 6. Two-sample Mendelian randomization analysis for the causal effects of soluble ST2 on Alzheimer's disease in Chinese and European-descent populations.
7. Supplementary Table 7. Meta-analysis of the rs1921622 A allele on Alzheimer's disease risk in overall, male, and female participants in the discovery cohorts.
8. Supplementary Table 8. Meta-analysis of the rs1921622 A allele on Alzheimer's disease risk in male and female *APOE*-ε4 carriers and noncarriers in the discovery cohorts.
9. Supplementary Table 9. Associations between the rs1921622 A allele and Alzheimer's disease-related endophenotypes in patients with Alzheimer's disease in the discovery cohorts.

Supplementary Notes

1. Late Onset Alzheimer's Disease Family Study

The Late Onset Alzheimer's Disease (LOAD) Family Study recruited families with 2 or more siblings with late-onset Alzheimer's disease (AD) as well as unrelated, age- and ethnicity-matched, non-demented controls. Patients with definite AD were diagnosed according to established neuropathological criteria (*i.e.,* CERAD score, Braak, Khachaturian, NIA-RI, or other established criteria). Probable AD or possible AD was determined according to the National Institute of Neurological and Communicative Disorders and Stroke (NINCDS)-Alzheimer's Disease and Related Disorders Association (ADRDA) criteria. Participants aged ≥55 years old were recruited. Please refer to the corresponding database of Genotypes and Phenotypes (dbGaP) project for details [1]. We retrieved genotype and phenotype data from the National Institutes of Health (NIH) dbGaP (accession number: phs000168.v2.p2), which includes 4 datasets. We merged the genotype information of 5,192 participants from datasets 1 (General Research Use); 3 (disease-specific, AD only); and 4 (disease-specific, AD and Non-Profit Use) before subsequent analysis (we excluded dataset 2 [$n = 28$] because of its small sample size). The individual genotypes were generated from the Human 610Quadv1_B Beadchip (Illumina). We only retained participants diagnosed with definite AD and healthy controls (HCs) for analysis, yielding a total of 2,695 participants ($n = 464$ patients with AD, $n = 2,231$ HCs) for the association study.

2. National Institute on Aging Alzheimer's Disease Centers cohorts

The study participants were ascertained and evaluated by the Clinical and Neuropathology Core of the 29 National Institute on Aging (NIA)-funded Alzheimer's Disease Centers (ADCs). These included autopsy-confirmed and clinically confirmed patients with Alzheimer's disease (AD) and cognitively normal healthy elderly. We retrieved genotype and phenotype data for these AD cohort participants ($n = 6,065$) from the National Institutes of Health (NIH) database of Genotypes and Phenotypes (dbGaP)

(accession number: phs000372.v2.p1); genotype information were generated from the Illumina Human660W-Quad Beadchip or HumanOmniExpress Array. All autopsied participants were ≥60 years old at death. The dementia in AD was determined using the Diagnostic and Statistical Manual of Mental Disorders, Fourth Edition (DSM-IV) criteria or a Clinical Dementia Rating ≥1. We retained all participants of European descent (≥60 years old, registered as definite AD as well as cognitively normal healthy controls [HCs]) for analysis, yielding a total of 4,902 participants (*n* = 3,431 patients with AD, *n* = 1,471 HCs) for the association study. Further details can be found in publications arising from the corresponding dbGaP project [2,3].

3. Alzheimer's Disease Neuroimaging Initiative cohort

We obtained genotype, biomarker, and magnetic resonance imaging (MRI) data from the Alzheimer's Disease Neuroimaging Initiative (ADNI) database (adni.loni.usc.edu). The ADNI was launched in 2003 as a public–private partnership and is led by Michael W. Weiner, MD. It is a 4-stage study that aims to assess the brain's structure and function using biomarker and clinical data from recruited participants between 55 and 90 years old from the United States and Canada. The ADNI study mainly comprises 3 sets of genetic data: ADNI-1 (*n* = 757; *n* = 499 for non-overlapped individuals with array genotyping data), ADNI-2/GO (*n* = 432), and ADNI whole-genome sequencing (WGS) (*n* = 808). We determined the phenotypes of the ADNI participants based on their latest diagnostic records (updated until July 2016). For the replication study of the effects of candidate genetic variants on Alzheimer's disease (AD) risk, we included all participants of European descent (≥60 years old, registered as definite AD as well as cognitively normal healthy controls [HCs] on the basis of the latest diagnostic records) in the ADNI WGS dataset and array genotyping data from ADNI-1, yielding a total of 668 participants (*n* = 378 patients with AD, *n* = 290 HCs). For the association studies between candidate genetic variants and brain volumetric data in the overall population and female *APOE-ε4* carriers, we only included

participants of European descent aged ≥60 years at the time of MRI assessment in the ADNI WGS dataset and array genotyping data from ADNI-1, yielding a total of 1,187 participants ($n = 206$ patients with AD, $n = 641$ patients with mild cognitive impairment [MCI], $n = 340$ HCs on the basis of diagnostic records at the time of MRI assessment).

4. Australian Imaging, Biomarkers and Lifestyle Study cohort

The Australian Imaging, Biomarkers and Lifestyle (AIBL) Study is a two-site (Melbourne and Perth), longitudinal cohort study that integrates neuroimaging, biomarker, neuropsychometric, and lifestyle data. The AIBL study population was selected from English-speaking volunteers who responded to media advertisements, or clinical cases that were referred to the study by a network of doctors. The AIBL study had strict selection criteria to eliminate, as much as possible, comorbidities such as vascular disease and diabetes, but no requirements on socio-economic status. Approximately 48% of the AIBL cohort reported more than 13 years of education. Clinical classification of the AIBL study was determined as previously described [4]. The study was approved by the Human Research Ethics Committee, Research Governance Unit, St Vincent's Health Australia, Australia, and was performed following all relevant ethical regulations. Written informed consent was obtained from all participants (or their legal guardians) before their participation in the study.

Amyloid-beta (Aβ)-positron emission tomography (PET) imaging for the AIBL Study was performed with three different radiotracers: PIB, FLUTE, or FBP. The PET methodology for each tracer has been previously described [5]. In brief, PET images were spatially normalized with CapAIBL using an adaptive atlas [6], and sampled using a preset template of narrow cortical regions of interest (ROIs). For semi-quantitative analysis, a volume of interest template was applied to the summed and spatially normalized PET images to obtain a standardized uptake value (SUV). The images were then scaled to the SUV of each tracer's recommended reference region to generate a tissue ratio termed "the SUV ratio"

(SUVR). A global measure of Aβ burden was computed using the mean SUVR in the frontal, superior parietal, lateral temporal, lateral occipital, and anterior and posterior cingulate regions. The SUVs were normalized to the cerebellar cortex for PIB, the whole cerebellum was used as the reference region for FBP [7], and the pons was used as the reference region for FLUTE [8] as suggested by the pharmaceutical companies that supplied each tracer. The SUVR was dichotomized as having a high ($A\beta^{+}$) or low ($A\beta^{-}$) Aβ burden, using a cut-off value that was determined for each tracer. Participants who underwent PIB were considered to have a high Aβ burden when SUVR ≥ 1.40, for FLUTE when SUVR ≥ 0.55, and for FBP when SUVR ≥ 1.05.

Other information we collected and used in this study includes results from cognitive assessments to derive the AIBL Preclinical Alzheimer's Cognitive Composite score, *APOE*-ε4 allele status, rs1921622 genotypes, age, sex, and relative information pertaining to the PET imaging. We also acquired individual magnetic resonance imaging (MRI) images with high-resolution 3D T1-weighted, T2-weighted, and fluid-attenuated inversion recovery images for AIBL participants. We examined the association between genotype and candidate endophenotypes by Wilcoxon rank-sum test. We used a linear mixed effect model to examine the effects of genotype on longitudinal brain atrophy, adjusting for age, sex, and scanner.

5. Genotype-Tissue Expression project dataset

The Genotype-Tissue Expression (GTEx) project was supported by the Common Fund of the Office of the Director of the National Institutes of Health as well as the NCI, NHGRI, NHLBI, NIDA, NIMH, and NINDS [9,10]. Data used for the analyses described herein were obtained from the database of Genotypes and Phenotypes (dbGaP) (phs000424.v6.p1). Among the donors, 83.1% were participants aged ≥40 years (40–49 years: 16.9%, 50–59 years: 34.6%, 60–69 years: 31.6%), and most donations were from people of European descent (84.3%). We incorporated genotypes and estimated transcript abundances at the gene and isoform levels into the present study.

6. Funding information of ADNI, LOAD, ADC and GTEx datasets

For the Alzheimer's Disease Neuroimaging Initiative (ADNI) dataset, data collection and sharing for this project were funded by ADNI (National Institutes of Health Grant number: U01-AG024904) and the Department of Defense (DOD) ADNI (DOD award number: W81XWH-12-2-0012). The ADNI is funded by the National Institute on Aging (NIA), the National Institute of Biomedical Imaging and Bioengineering, and through generous contributions from the following organizations: AbbVie, Alzheimer's Association; Alzheimer's Drug Discovery Foundation; Araclon Biotech; BioClinica, Inc.; Biogen; Bristol-Myers Squibb Company; CereSpir, Inc.; Cogstate; Eisai, Inc.; Elan Pharmaceuticals, Inc.; Eli Lilly and Company; EuroImmun; F. Hoffmann–La Roche Ltd. and its affiliated company, Genentech, Inc.; Fujirebio; GE Healthcare; IXICO, Ltd.; Janssen Alzheimer Immunotherapy Research & Development, LLC.; Johnson & Johnson Pharmaceutical Research & Development, LLC.; Lumosity; Lundbeck; Merck & Co., Inc.; Meso Scale Diagnostics, LLC.; NeuroRx Research; Neurotrack Technologies; Novartis Pharmaceuticals Corporation; Pfizer, Inc.; Piramal Imaging; Servier; Takeda Pharmaceutical Company; and Transition Therapeutics. The Canadian Institutes of Health Research provides funds to support ADNI clinical sites in Canada. Private-sector contributions are facilitated by the Foundation for the National Institutes of Health (www.fnih.org). The grantee organization is the Northern California Institute for Research and Education, and the study is coordinated by the Alzheimer's Therapeutic Research Institute at the University of Southern California. ADNI data are disseminated by the Laboratory for Neuro Imaging at the University of Southern California. For the Alzheimer's Disease Genetics Consortium (ADGC) Genome Wide Association Study–NIA Alzheimer's Disease Centers Cohorts (ADC datasets), funding support for the ADGC was provided through the NIA Division of Neuroscience (grant number: U01-AG032984). For the NIA–Late Onset Alzheimer's Disease Family Study (LOAD dataset), funding support for the "Genetic Consortium for Late Onset Alzheimer's Disease"

was provided through the Division of Neuroscience, NIA. The Genetic Consortium for Late Onset Alzheimer's Disease includes a genome-wide association study funded as part of the Division of Neuroscience, NIA. Finally, the Genetic Consortium for Late Onset Alzheimer's Disease provided assistance with phenotype harmonization and genotype cleaning as well as general study coordination. The Genotype-Tissue Expression (GTEx) project was supported by the Common Fund of the Office of the Director of the National Institutes of Health, and by NCI, NHGRI, NHLBI, NIDA, NIMH, and NINDS.

7. Investigators of the ADNI I, GO, II, and III studies

Andrew J. Saykin, Arthur W. Toga, Bret Borowski, Chad Ward, Charles DeCArli, Chet Mathis, Clifford R. Jack, Jr., Danielle Harvey, David Holtzman, David Jones, Devon Gessert, Eli Lilly, Eric M. Reiman, Erin Franklin, Franz Hefti, Greg Sorensen, Gustavo Jimenez, Howard Fillit, Jeff Gunter, Jennifer Salazar, John Hsiao, John Morris, John Q. Trojanowki, Karen Crawford, Kejal Kantarci, Kelley Faber, Kelly Harless, Kewei Chen, Kwangsik Nho, Laurel Beckett, Lean Thal, Leon Thal, Leslie M. Shaw, Lew Kuller, Li Shen, Lindsey Hergesheimer, Lisa Taylor-Reinwald, M. Marcel Mesulam, Magdalena Korecka, Marc Raichle, Maria Carrillo, Marilyn Albert, Matt Senjem, Matthew Bernstein, Michael Donohue, Michael Weiner, Michal Figurski, Neil Buckholtz, Nick Fox, Nigel J. Cairns, Norbert Schuff, Norm Foster, Paul Aisen, Paul Thompson, Peter Davies, Peter J. Snyder, Peter Snyder, Prashanthi Vemuri, Richard Frank, Robert A. Koeppe, Robert C. Green, Ronald Petersen, Sarah Walter, Scott Neu, Steven Paul, Steven Potkin, Sungeun Kim, Tatiana M. Foroud, Tom Montine, Virginia Lee, William Jagust, William Potter, Yuliana Cabrera, Zaven Khachaturian

8. Investigators of ADNI from university and institutes

Adam Fleisher, Aimee Pierce, Akiva Mintz, Alan Lerner, Alexander Norbash, Allan I. Levey, Allyson Rosen, Amanda Smith, Anaztasia Ulysse, Andrew E. Budson, Andrew Kertesz, Angela Oliver, Ann Marie Hake, Anna Burke, Antero Sarrael, Anton P. Porsteinsson, Ashley Lamb, Athena Lee, Balebail Ashok Raj, Barton Lane, Beatriz Yanez, Beau Ances, Benita Mudge, Betty Lind, Bojana Stefanovic, Bonnie S. Goldstein, Borna Bonakdarpour, Brandy R. Matthews, Brian R. Ott, Brigid Reynolds, Bruce L. Miller, Bryan M. Spann, Carl Sadowsky, Charles Bernick, Charles D. Smith, Chiadi Onyike, Chris (Chinthaka) Heyn, Chris Hosein, Christi Leach, Christine M. Belden, Christopher H. van Dyck, Christopher M. Clark, Chuang-Kuo Wu, Colleen S. Albers, Connie Brand, Courtney Bodge, Curtis Tatsuoka, Cynthia M. Carlsson, Dana Mathews, Daniel D'Agostino II, Daniel H.S. Silverman, Daniel Marson, David A. Wolk, David Bachman, David Clark, David Geldmacher, David Hart, David Knopman, David Perry, David Winkfield, Delwyn D. Miller, Diana Kerwin, Dick Drost, Donna M. Simpson, Donna Munic, Douglas W. Scharre, Dr. Rob Bartha, Dzintra Celmins, Earl A. Zimmerman, Edmond Teng, Edward Coleman, Edward Zamrini, Effie Mitsis, Elizabeth Finger, Elizabeth Oates, Elizabeth Sosa, Ellen Woo, Emily Rogalski, Evan Fletcher, Francine Parfitt, Gaby Thai, Gad A. Marshall, Gary Conrad, Geoffrey Tremont, George Bartzokis, Ging-Yuek Robin Hsiung, Gloria Chiang, Godfrey D. Pearlson, Greg Jicha, Helen Vanderswag, Hillel Grossman, Horacio Capote, Howard Bergman, Howard Chertkow, Howard Feldman, Howard J. Rosen, Hristina Koleva, Hyungsub Shim, Irina Rachinsky, Jacobo Mintzer, Jaimie Ziolkowski, James Brewer, James J. Lah, Jamika Singleton-Garvin, Janet S. Cellar, Jared R. Brosch, Jared Tinklenberg, Jason H. Karlawish, Javier Villanueva-Meyer, Jeffrey A. Kaye, Jeffrey M. Burns, Jeffrey R. Petrella, Jerome Yesavage, Joanne Allard, Joanne L. Lord, Joel Hetelle, John Brockington, John C. Morris, John Olichney, John Rogers, Joseph Quinn, Joseph S. Kass, Joy L. Taylor, Judith L. Heidebrink, Karen Anderson, Karen Blank, Karen Ekstam Smith, Karen L. Bell, Kathleen Johnson, Kathleen Tingus, Kathryn DeMarco, Kaycee M. Sink, Keith A. Johnson, Kelly M. Makino, Kenneth Spicer, Ki Won Nam, Kim Martin, Kim Poki-Walker, Kris Johnson, Kristin Fargher, Kristine

Lipowski, Kyle Womack, Laura A. Flashman, Lawrence S. Honig, Liana Apostolova, Liberty Teodoro, Lisa C. Silbert, Lisa Ravdin, Lon S. Schneider, Lori A. Daiello, M. Saleem Ismail, Marc Seltzer, Marek-Marsel Mesulam, Maria Carroll, Maria Kataki, Maria T. GreigCusto, Marissa Natelson Love, Mark A. Mintun, Martin R. Farlow, Martin Sadowski, Mary L. Creech, Mary L. Hynes, Mary Quiceno, MaryAnn Oakley, Mauricio Becerra, Megan Witbracht, Melanie Keltz, Melissa Lamar, Mia Yang, Michael Borrie, Michael Lin, Michele Assaly, Michelle Rainka, Mimi Dang, Mohammed O. Sheikh, Mrunalini Gaikwad, Munir Chowdhury, Nadira Trncic, Nancy Johnson, Nancy Kowalksi, Nathaniel Pacini, Neil Kowall, Neill R Graff-Radford, Norman Relkin, Ntekim E. Oyonumo, Nunzio Pomara, Olga James, Olu Ogunlana, Oscar L. Lopez, Owen Carmichael, P. Murali Doraiswamy, Parianne Fatica, Patricia Lynn Johnson, Patricia Samuels, Paul Malloy, Paula Ogrocki, Pauline Maillard, Peter Hardy, Pierre Tariot, Po H. Lu, Pradeep Varma, Rachelle S. Doody, Raina Carter, Raj C. Shah, Randall Griffith, Randy Yeh, Ranjan Duara, Rawan Tarawneh, Raymond Scott Turner, Raymundo Hernando, Reisa A., Richard E. Carson, Riham El Khouli, Robert B. Santulli, Ronald Killiany, Rosemarie Rodriguez, Russell H. Swerdlow, Salvador Borges-Neto, Sandra Black, Sandra Weintraub, Sanjay Asthana, Sanjeev Vaishnavi, Sara Dolen, Sara S. Mason, Scott Herring, Sherye A. Sirrel, Smita Kittur, Sonia Pawluczyk, Stacy Schneider, Stephanie Kielb, Stephanie Reeder, Stephen Correia, Stephen Pasternack, Stephen Pasternak, Stephen Salloway, Sterling Johnson, Steven Chao, Steven E. Arnold, Susan K. Schultz, Susan Rountree, T-Y Lee, Terence Z. Wong, Teresa Villena, Thomas O. Obisesan, Valory Pavlik, Vernice Bates, Vesna Sossi, Victoria Shibley, William M. Brooks, William Pavlosky, Yaakov Stern

9. Biomarkers Consortium Project team members of ADNI

Adam Simon, Ashok Dongre, Bob Dean, Brad Navia, Dan Spellman, David Lee, David Shera, Eric Siemers, Eve Pickering, Frank Swenson, Fred Immerman, George Nomikos, Holly Soares, Hong Wan, Jeff Seeburger, Jeff Waring, John Trojanowski, Judy Siuciak, Kevin Duffin, Les Shaw, Li-San Wang,

Madhav Thambisetty, Marc Walton, Mary Savage, Mats Ferm, Max Kuhn, Neil Buckholtz, Panos Zagouras, Patricia Cole, Ron Hendrickson, Sharon Xie, Sophie Allauzen, Walter Koroshetz, William Potter

Supplementary Figures

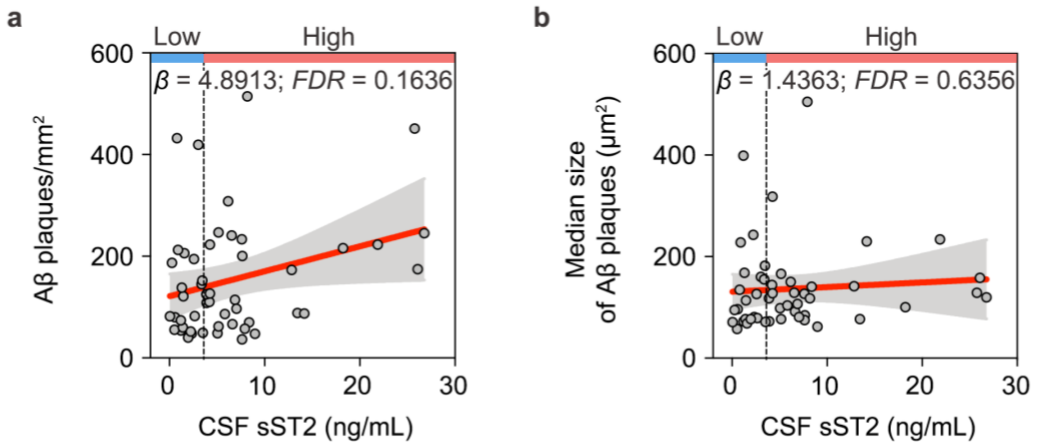

Supplementary Fig. 1. Associations between cerebrospinal fluid soluble ST2 levels and number and size of amyloid-beta plaques in patients with Alzheimer's disease in the UK Brain Banks Network cohort. Scatterplots showing the associations between cerebrospinal fluid **(**CSF) soluble ST2 (sST2) levels and the number (a) and median size (b) of amyloid-beta (Aβ) plaques (n = 51 patients with Alzheimer's disease [AD] from the UK Brain Banks Network [UKBBN] cohort). The regression lines and 95% confidence intervals are indicated in red and gray, respectively. The vertical dashed lines indicate the CSF sST2 level (3.6 ng/mL) with the largest Youden's index value for distinguishing healthy controls (HCs) from patients with Alzheimer's disease (AD). Linear regression test, adjusted for age, sex, and postmortem duration (PMD), with multiple testing correction. β, effect size; *FDR*, false discovery rate.

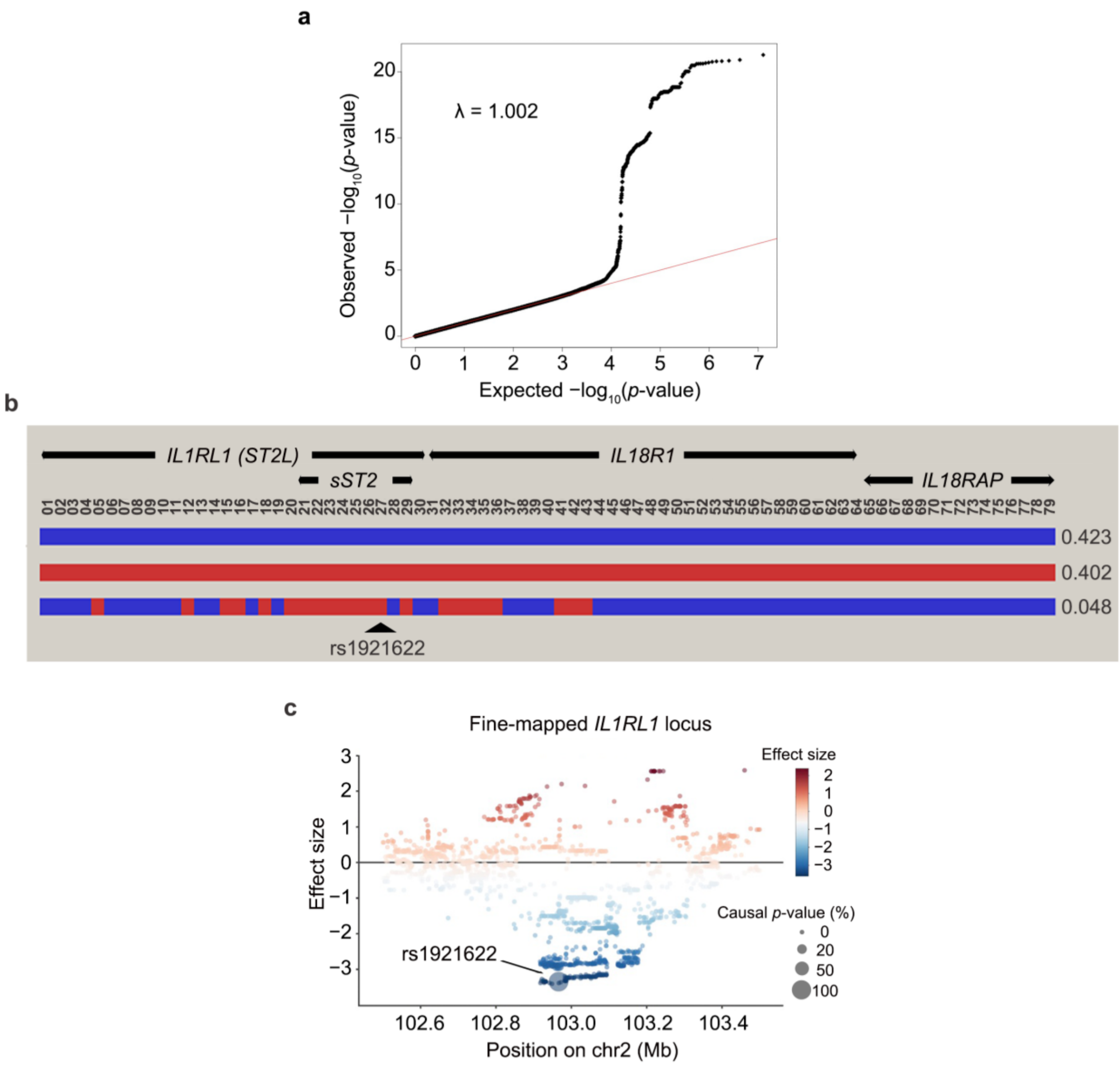

Supplementary Fig. 2. Fine-mapping analysis of soluble ST2-associated genetic variants in the *IL1RL1* gene. (a) Quantile-quantile (Q–Q) plot showing the *p*-value distribution of the genome-wide association study results. The genomic inflation factor (λ) is shown. (b) Haplotype analysis of the 79 genetic variants linked with rs1921622 ($r^2 > 0.7$). Each numbered column represents 1 of the 79 variants; red and blue indicate the minor and major alleles, respectively. Each row represents a particular haplotype defined by a specific combination of major and minor alleles in the haplotype block; the haplotype frequency is indicated on the right, and the gene loci (±10 kbp) is indicated on the top. The triangle indicates the location of rs1921622. (c) Fine-mapping plots showing the associations between variants in the *IL1RL1* gene and plasma soluble ST2 (sST2) level. Dot color intensity indicates the effect size of individual variants on plasma sST2 level, and dot size indicates the probability of a variant exerting its causal effect.

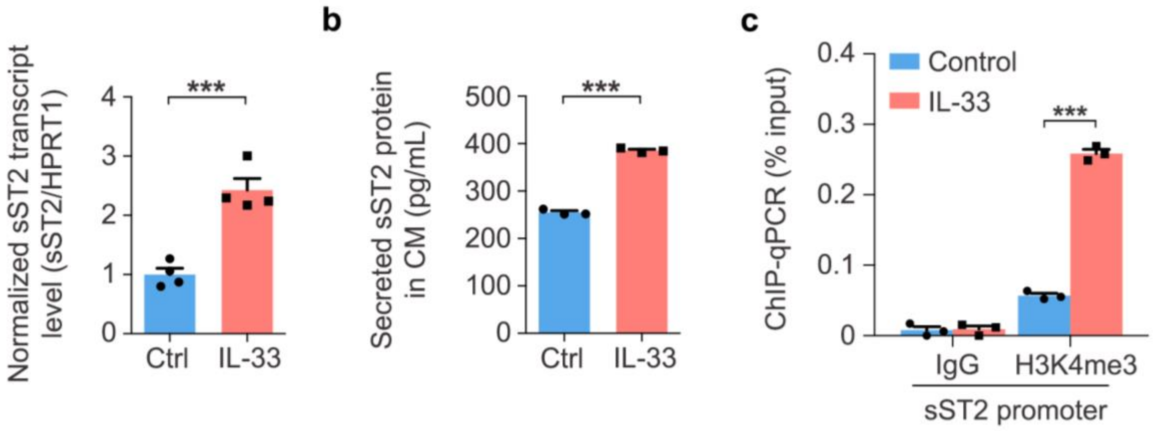

Supplementary Fig. 3. Interleukin 33 induces the expression and secretion of soluble ST2 in brain endothelial cells. (a, b) Administration of IL-33 enhances the expression (*n* = 4 per group) (a) and secretion (*n* = 3 per group) (b) of soluble ST2 (sST2) in hCMEC/D3 cells. *T* = 6.484 and 28.89, *P* = 6.00E−4 and *P* < 1.00E−4 for sST2 transcript and protein levels, respectively. CM, conditioned medium; Ctrl, control group. (c) Chromatin immunoprecipitation (ChIP)-quantitative PCR (qPCR) analysis of H3K4me3 changes at the sST2 promoter region after IL-33 administration for 24 h in hCMEC/D3 cells (*n* = 3 per group). *T* = 29.69, *P* < 1.00E−4. Data in bar charts are mean + SEM. Statistical tests are performed as two-sided unpaired Student's *t*-test. **P* < 0.05, ***P* < 0.01, ****P* < 0.001.

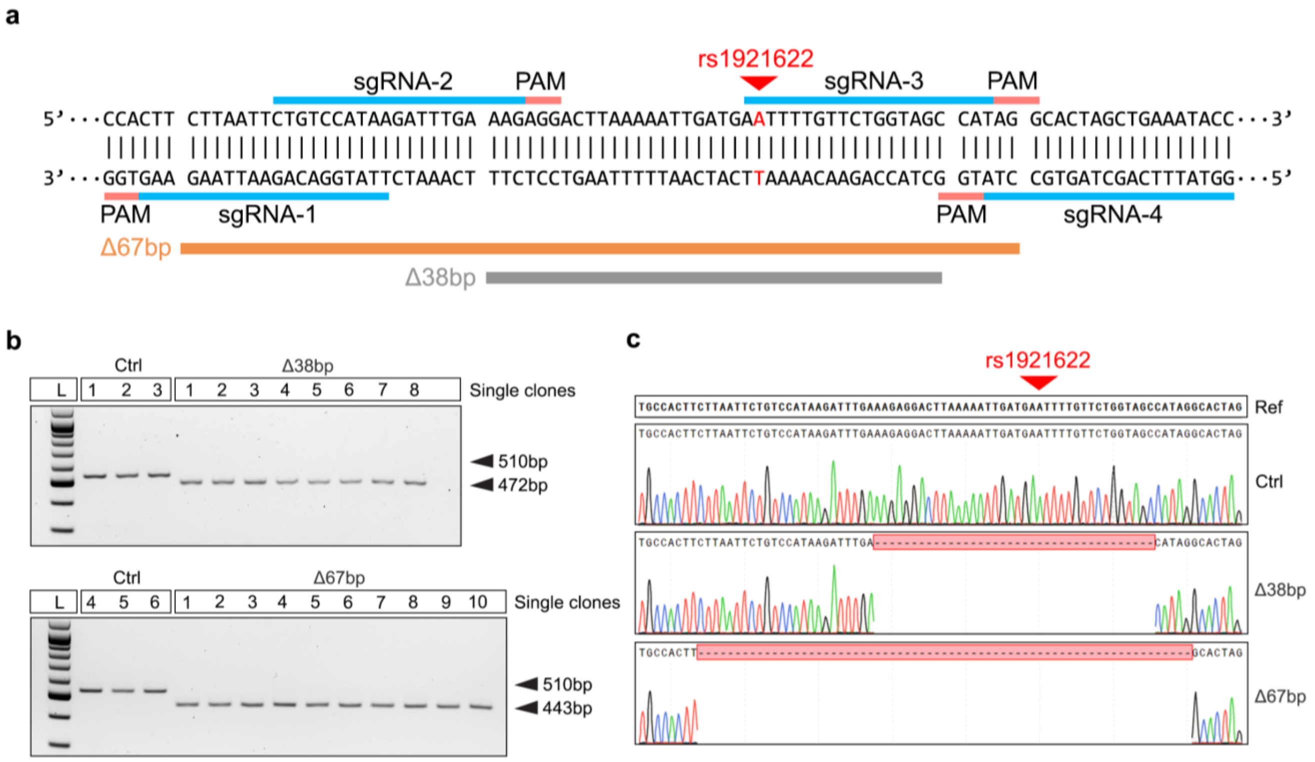

Supplementary Fig. 4. Validation of the CRISPR/Cas9-based target deletion at the rs1921622-containing region in hCMEC/D3 cells. (a) Diagram showing the locations of the 2 single guide RNA (sgRNA) pairs (*i.e.,* sgRNA-1 and sgRNA-4 for 67-bp deletion [Δ67bp], and sgRNA-2 and sgRNA-3 for 38-bp deletion [Δ38bp]) targeting the rs1921622-containing region (red). PAM, protospacer adjacent motif. (b) Gel images of single clones of isogenic control lines (Ctrl; $n = 6$), 38-bp deletion lines (Δ38bp; $n = 8$), and 67-bp deletion lines (Δ67bp; $n = 10$). L, DNA ladder. (c) Sanger validation of single clones.

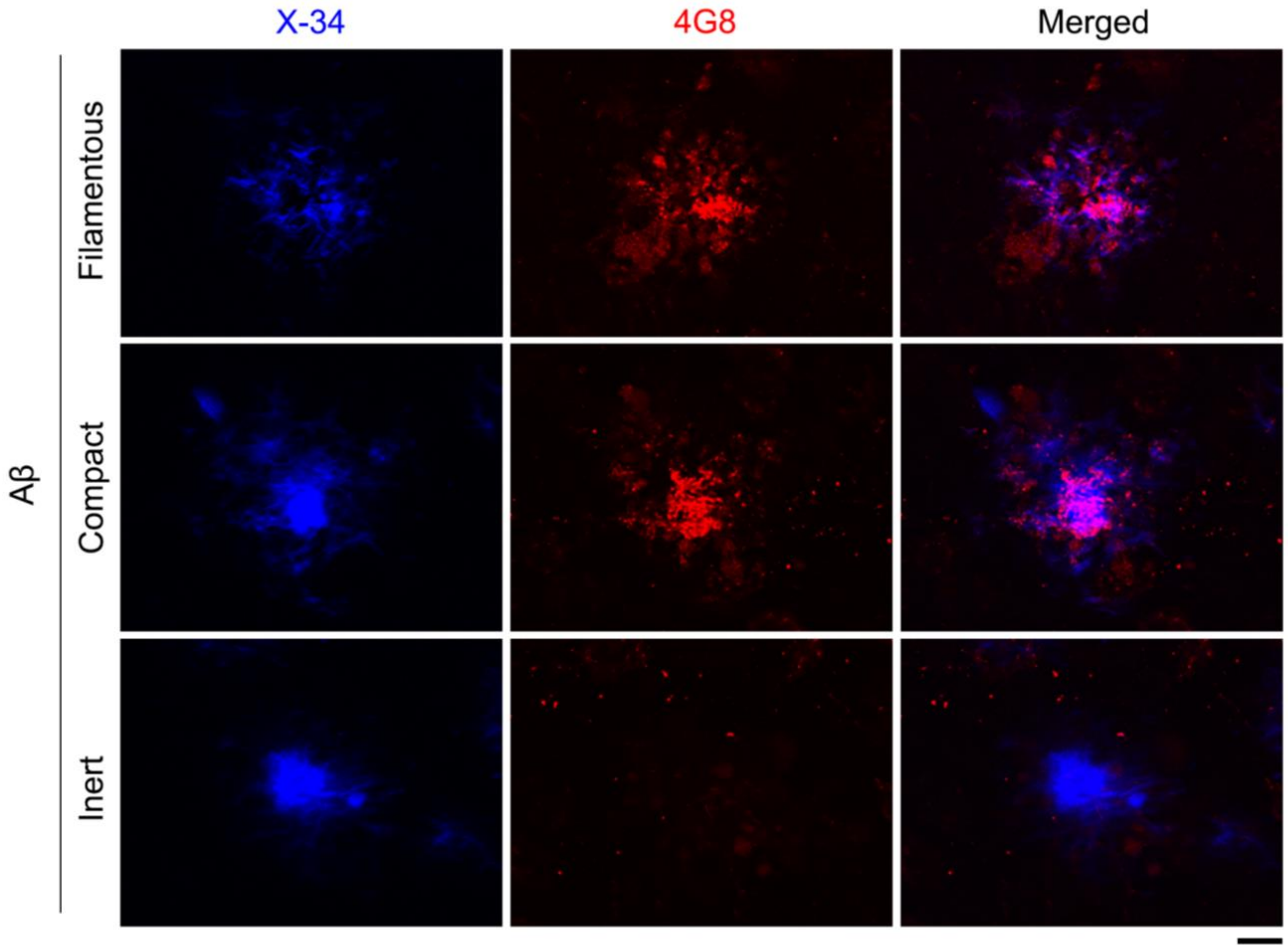

Supplementary Fig. 5. Representative images of filamentous, compact, and inert amyloid-beta plaques in 5XFAD mice. Confocal images of X-34–stained amyloid-beta (Aβ) deposits (blue) and 4G8-labeled Aβ (red) in the cortices of 4-month-old female 5XFAD mice (images from 6 control mice and 7 sST2-treated mice in two independent experiments were obtained). Scale bar, 10 μm.

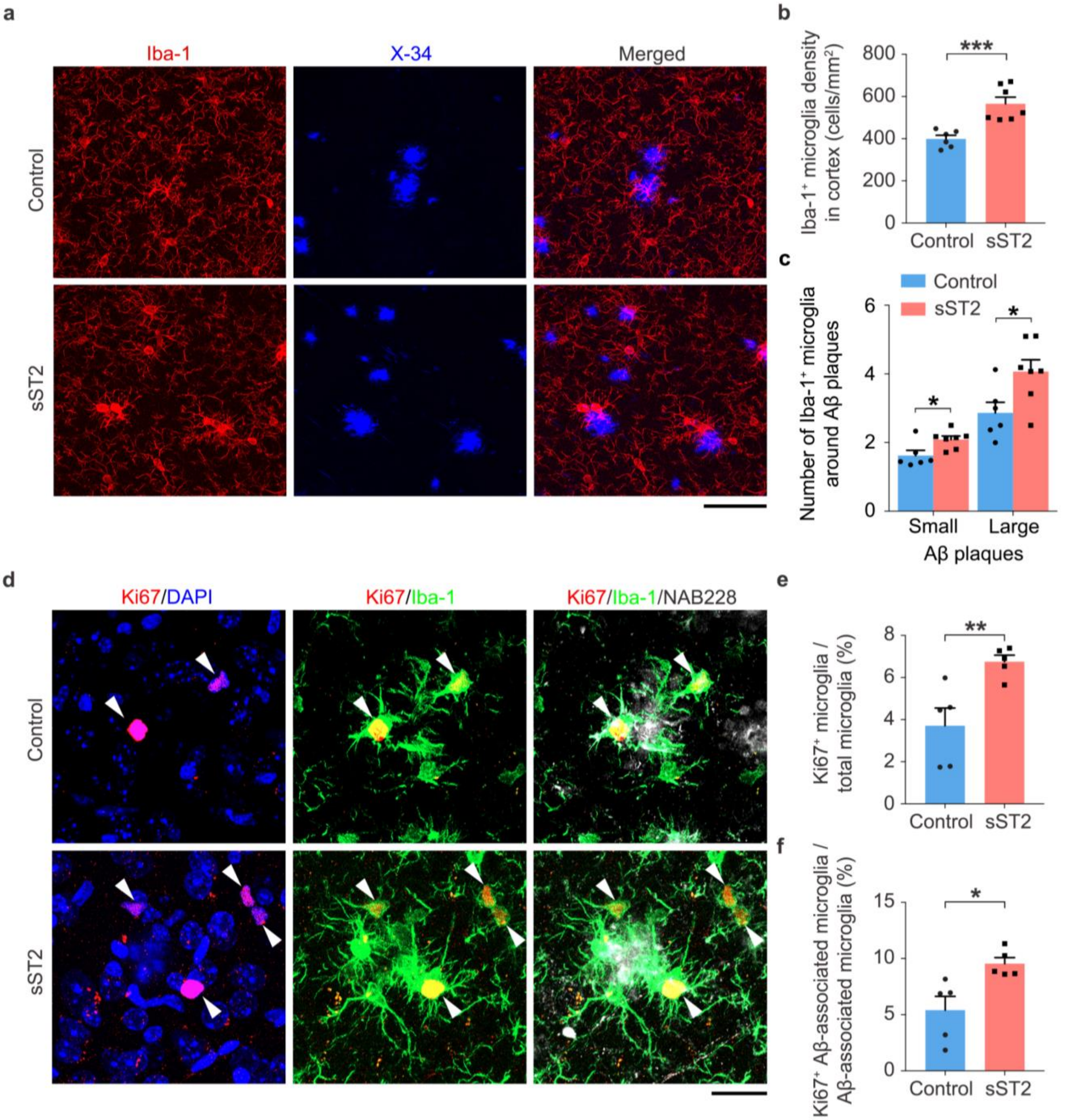

Supplementary Fig. 6. Elevated brain soluble ST2 level leads to increased number of microglia in female 5XFAD mice. (a, b) Brain sST2 increases the number of microglia in female 5XFAD mice. Representative images (a) and quantification (b) of Iba-1^+ microglia in the cortices of 4-month-old female 5XFAD mice after 28-day intracerebroventricular delivery of sST2-Fc or Fc as a control (control: n = 6 mice, sST2: n = 7 mice). T = 4.491, P = 9.00E−4. Scale bar, 50 µm. (c) Quantification of microglia around small (*i.e.,* radius ≤8 µm) and large (*i.e.,* radius >8 µm) Aβ plaques (control: n = 6 mice, sST2: n = 7 mice). T = 2.661 and 2.551, respectively; P = 0.022 and 0.027, respectively. (d−f) Brain sST2 increases microglial proliferation in female 5XFAD mice. Representative images (d) and quantification of $Ki67^+$ microglia (e; white arrowheads) and $Ki67^+$ Aβ-associated microglia (f) in the cortices of 4-month-old female 5XFAD mice after 28-day intracerebroventricular delivery of sST2-Fc or Fc as a control (control: n = 5 mice, sST2: n = 5 mice). T = 3.400 and 3.097, respectively; P = 0.009 and 0.014,

respectively. Scale bar, 20 μm. Data in bar charts are mean + SEM. Statistical tests are performed as two-sided unpaired Student's *t*-test. *$P < 0.05$, **$P < 0.01$, ***$P < 0.001$.

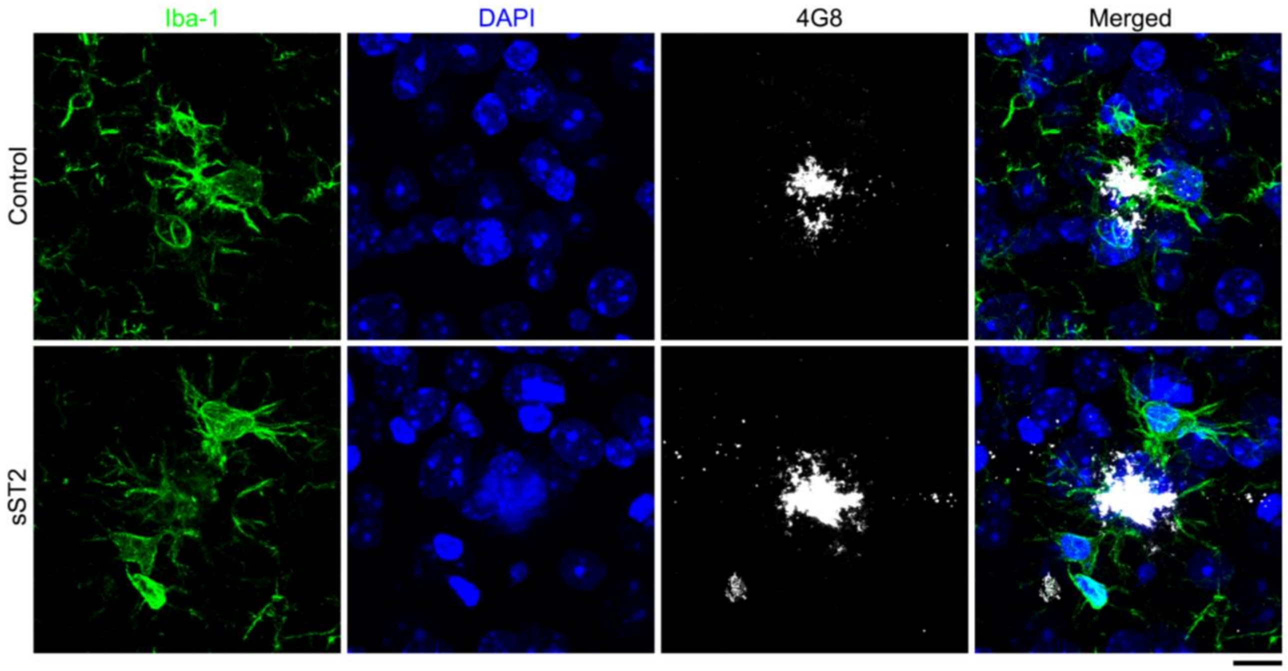

Supplementary Fig. 7. Representative images showing the co-staining of microglia and amyloid-beta plaques in 5XFAD mice. Confocal images of Iba-1$^+$ microglia (green), DAPI-stained nucleus (blue), and 4G8-stained Aβ deposits (white) in the cortices of 4-month-old female 5XFAD mice after 28-day intracerebroventricular delivery of sST2-Fc or Fc as a control (images from 6 control mice and 7 sST2-treated mice in two independent experiments were obtained). Scale bar, 10 μm.

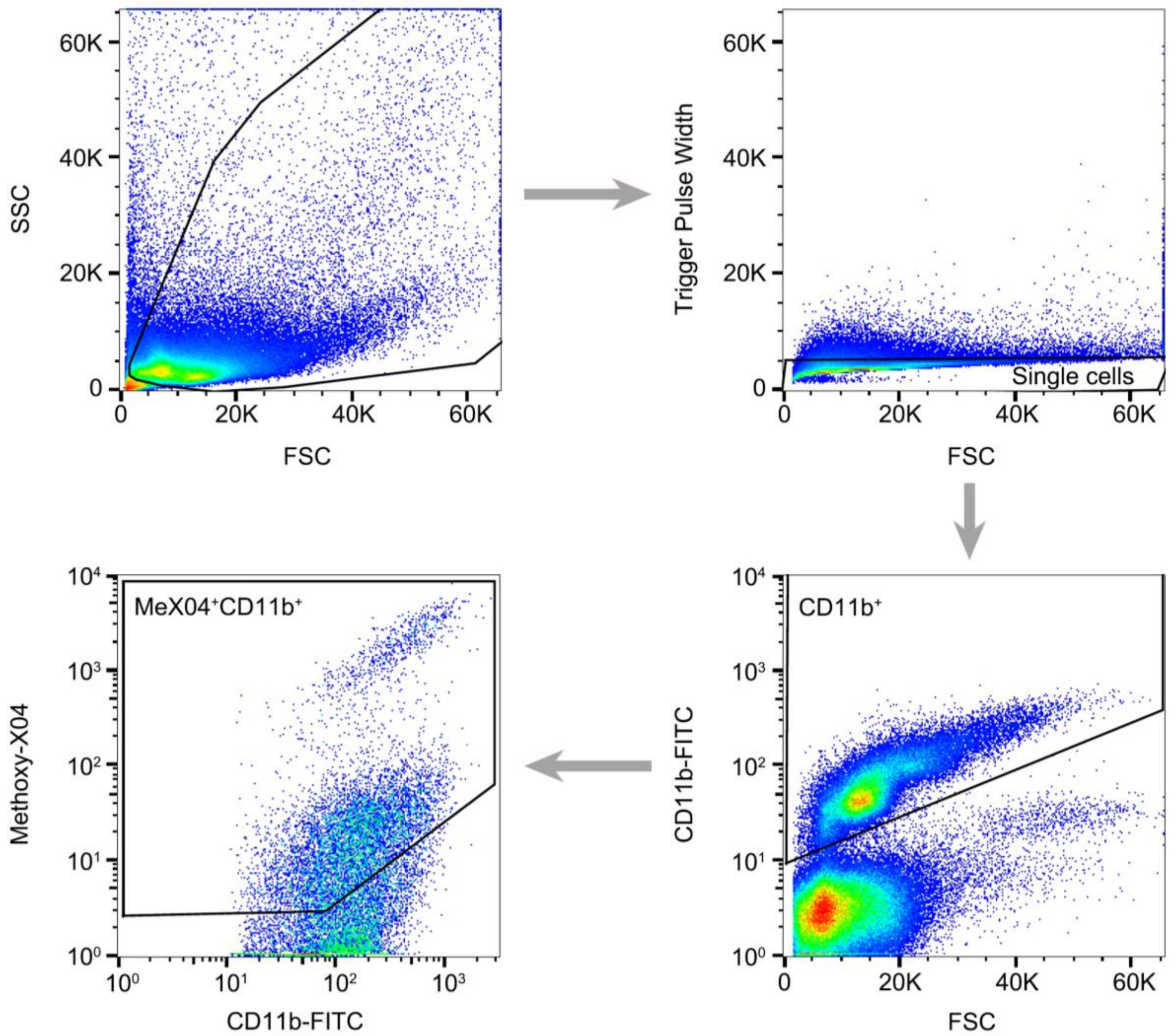

Supplementary Fig. 8. The gating strategy for amyloid-beta (Aβ)$^{+}$ microglia. Cells were gated on forward (FSC = size) and sideward scatter (SSC = internal structure). We used FSC and trigger pulse width to discriminate single cells from cell doublets or aggregates. We used unstained controls to identify CD11b^{+} cell populations. We used samples from C57BL6J mice to identify methoxy-X04^{+} cell populations.

Supplementary Tables

Supplementary Table 1. Demographic characteristics of the Chinese_cohort_1.

		**Healthy control**	**Alzheimer's disease**
Sample size (*n*)		345	345
Basic information	Age, years (SD)	73.12 (4.71)	80.53 (5.96)
	Sex, male (%)	140 (40.58%)	110 (31.88%)
	Education level, years (SD)	8.16 (5.06)	4.85 (4.70)
	MoCA score (SD)	23.62 (3.31)	12.61 (5.42)
	APOE-ε4, allele frequency	7.97%	19.71%
	APOE-ε2, allele frequency	10.43%	5.07%
CVD records	Heart disease (%)	30 (8.70%)	57 (16.52%)
	Hypertension (%)	211 (61.16%)	207 (60.00%)
	Diabetes Mellitus (%)	76 (22.03%)	106 (30.72%)
	Hyperlipidemia (%)	104 (30.14%)	146 (42.32%)
	No CVDs (%)	97 (28.12%)	86 (24.93%)
Data available (*n*)	Plasma sST2 (mean level)	336 (12.30 ng/mL)	277 (14.09 ng/mL)
	Plasma P-tau181 (mean level)	134 (2.35 pg/mL)	156 (4.10 pg/mL)
	Plasma NfL (mean level)	135 (16.19 pg/mL)	154 (29.12 pg/mL)
	Gray matter MRI (mean %ICV)	115 (40.75%)	77 (39.44%)
	Genotypes (WGS)	194	233
	Genotypes (*APOE*-ε2, *APOE*-ε4, rs1921622 array)	151	112

CVD, cardiovascular disease; ICV, intracranial volume; MoCA, Montreal Cognitive Assessment; MRI, magnetic resonance imaging; NfL, neurofilament light polypeptide; SD, standard deviation; sST2, soluble ST2; WGS, whole-genome sequencing.

Supplementary Table 2. Associations between plasma soluble ST2 levels and Alzheimer's disease and its related endophenotypes in the Chinese_cohort_1. Linear regression test, adjusted for age, sex, status of cardiovascular diseases (CVDs) (*i.e.,* heart disease, hypertension, diabetes mellitus, and hyperlipidemia), body mass index (BMI), and education level, followed by multiple testing correction.

	AD vs. HC			Gray matter volumes			Plasma P-tau181 levels			Plasma NfL levels		
**Sub-groups**	***n***	***β* (SE)**	***FDR***	***n***	***β* (SE)**	***FDR***	***n***	***β* (SE)**	***FDR***	***n***	***β* (SE)**	***FDR***
Overall	613	2.072 (0.560)	**8.31E−4**	192	−0.695 (0.192)	**0.003**	290	0.413 (0.121)	**0.005**	289	0.107 (0.031)	**0.004**
Males	216	1.926 (1.256)	0.148	106	−0.164 (0.399)	0.795	118	0.216 (0.172)	0.222	124	0.085 (0.044)	0.075
Females	397	2.235 (0.568)	**6.97E−4**	86	−0.482 (0.186)	**0.039**	172	0.642 (0.246)	**0.033**	165	0.130 (0.048)	**0.022**
Male *APOE*-ε4 carriers	59	2.034 (0.734)	**0.014**	29	−0.386 (0.631)	0.764	30	0.545 (0.436)	0.222	34	0.113 (0.156)	0.554
Male *APOE*-ε4 noncarriers	157	1.098 (1.537)	0.476	77	−0.016 (0.478)	0.973	88	−0.586 (0.330)	0.111	90	0.001 (0.057)	0.991
Female *APOE*-ε4 carriers	111	3.833 (1.192)	**0.004**	41	−0.528 (0.379)	0.402	54	0.668 (0.263)	**0.033**	58	0.153 (0.057)	**0.022**
Female *APOE*-ε4 noncarriers	286	1.811 (0.835)	**0.043**	45	−0.442 (0.467)	0.610	118	0.361 (0.203)	0.111	107	0.116 (0.053)	0.057

AD, Alzheimer's disease; HC, healthy control; NfL, neurofilament light polypeptide; *β*, effect size; SE, standard error; *FDR*, false discovery rate. Bold and red text indicates statistical significance at a cutoff of *FDR* < 0.05.

Supplementary Table 3. Candidate genetic variants in the *IL1RL1* gene associated with plasma soluble ST2 level after fine mapping (causal probability >0.001). Linear regression test, adjusted for age, sex, AD diagnosis, and population structure, with fine-mapping analysis.

Position	SNP	β (SE)	*P*-value	Causal probability[a]
chr2:102966067	rs1921622	−3.346 (0.327)	5.35E−22	0.9999
chr2:102960584	rs12712142	−2.919 (0.344)	4.41E−16	0.1745
chr2:102964742	rs13017455	−2.919 (0.344)	4.41E−16	0.1745
chr2:102967844	rs11123923	−2.919 (0.344)	4.41E−16	0.1745
chr2:102931826	rs13020553	−2.907 (0.344)	4.75E−16	0.0556
chr2:102932562	rs950880	−2.907 (0.344)	4.75E−16	0.0556
chr2:102939036	rs13001325	−2.890 (0.344)	7.72E−16	0.0534
chr2:102948470	rs1420104	−2.890 (0.344)	7.72E−16	0.0534
chr2:102949161	rs12479210	−2.890 (0.344)	7.72E−16	0.0534
chr2:102960485	rs13001714	−2.890 (0.344)	7.72E−16	0.0534
chr2:102963072	rs6543119	−2.904 (0.344)	5.91E−16	0.0508
chr2:102957716	rs1420101	−2.895 (0.344)	6.87E−16	0.0253
chr2:102950822	rs13019081	−2.905 (0.346)	8.44E−16	0.0250
chr2:102926362	rs12470864	−2.880 (0.343)	7.15E−16	0.0168
chr2:102969606	rs35224028	−2.878 (0.342)	5.99E−16	0.0154
chr2:102974417	rs12998521	−2.877 (0.346)	1.26E−15	0.0044
chr2:102974129	rs12999364	−2.858 (0.347)	2.30E−15	0.0022
chr2:102975336	rs12987977	−2.858 (0.347)	2.30E−15	0.0022
chr2:102979028	rs2287037	−2.858 (0.347)	2.30E−15	0.0022
chr2:102983247	rs4851569	−2.858 (0.347)	2.30E−15	0.0022
chr2:102984671	rs1882348	−2.858 (0.347)	2.30E−15	0.0022
chr2:102984279	rs1420098	−2.854 (0.346)	2.12E−15	0.0019

β, effect size; SE, standard error; SNP, single nucleotide polymorphism; sST2, soluble ST2.

[a] Probability of being a putative causal variant for plasma sST2 level.

Supplementary Table 4. Associations between the rs1921622 A allele and soluble ST2 and full-length ST2 transcript levels in human tissues. Linear regression test, adjusted for age, sex, RNA integrity, and population structure.

#	Tissue type	Tissue subregion	*n*	sST2 (ENST00000311734.2)[a]			ST2L (ENST00000233954.1)[a]		
				Avg. transcript level[b]	*β* (SE)	*P*	Avg. transcript level[b]	*β* (SE)	*P*
1	Adipose	Visceral (omentum)	213	11.485	0.035 (0.106)	0.741	0.270	−0.087 (0.107)	0.418
2		Subcutaneous	332	8.333	0.023 (0.077)	0.762	-	-	-
3	Adrenal gland	Adrenal gland	148	23.138	0.163 (0.135)	0.228	-	-	-
4	Artery	Aorta	226	3.024	−0.091 (0.098)	0.355	-	-	-
5		Tibial	319	1.168	−0.053 (0.080)	0.508	-	-	-
6		Coronary	124	4.329	0.065 (0.142)	0.647	-	-	-
7	Brain	Anterior cingulate cortex (BA24)	86	3.156	−0.179 (0.153)	0.241	-	-	-
8		Caudate (basal ganglia)	119	2.461	−0.265 (0.135)	0.051	-	-	-
9		Cerebellar hemisphere	104	1.031	−0.194 (0.134)	0.148	-	-	-
10		Cerebellum	121	1.229	−0.309 (0.121)	**0.011**	-	-	-
11		Cortex	110	2.576	−0.275 (0.135)	**0.046**	-	-	-
12		Amygdala	69	2.688	−0.388 (0.176)	**0.031**	-	-	-
13		Frontal Cortex (BA9)	108	2.848	−0.319 (0.143)	**0.027**	-	-	-
14		Spinal cord (cervical c-1)	67	3.306	−0.521 (0.184)	**0.006**	-	-	-
15		Hippocampus	92	2.226	−0.327 (0.161)	**0.045**	-	-	-
16		Nucleus accumbens (basal ganglia)	108	2.317	−0.390 (0.145)	**0.008**	-	-	-
17		Substantia nigra	64	5.066	−0.344 (0.155)	**0.029**	-	-	-
18		Putamen (basal ganglia)	91	2.205	−0.290 (0.131)	**0.030**	-	-	-
19		Hypothalamus	91	3.348	−0.258 (0.142)	0.071	-	-	-
20	Breast	Mammary tissue	193	8.720	−0.036 (0.096)	0.705	-	-	-

#	Tissue type	Tissue subregion	*n*	sST2 (ENST00000311734.2)[a]			ST2L (ENST00000233954.1)[a]		
				Avg. transcript level[b]	*β* (SE)	*P*	Avg. transcript level[b]	*β* (SE)	*P*
21	Cell line	Transformed fibroblasts	273	4.102	−0.034 (0.084)	0.684	-	-	-
22		EBV-transformed lymphocytes	120	-	-	-	-	-	-
23	Colon	Transverse	185	3.311	−0.127 (0.110)	0.249	-	-	-
24		Sigmoid	159	2.043	0.027 (0.122)	0.820	-	-	-
25	Esophagus	Gastroesophageal junction	155	3.690	−0.188 (0.120)	0.120	0.327	−0.056 (0.097)	0.565
26		Muscularis	260	3.059	−0.105 (0.087)	0.230	0.301	0.010 (0.078)	0.900
27		Mucosa	300	1.464	−0.090 (0.092)	0.326	-	-	-
28	Heart	Left ventricle	240	7.199	−0.022 (0.101)	0.824	-	-	-
29		Atrial appendage	194	8.955	0.017 (0.117)	0.885	-	-	-
30	Liver	Liver	120	9.797	0.169 (0.146)	0.251	-	-	-
31	Lung	Lung	331	98.940	−0.239 (0.077)	**0.002**	1.168	−0.021 (0.074)	0.772
32	Minor salivary gland	Minor salivary gland	65	1.333	−0.219 (0.216)	0.316	-	-	-
33	Muscle	Skeletal muscle	410	0.935	−0.113 (0.068)	0.099	-	-	-
34	Nerve	Tibial nerve	294	2.165	−0.036 (0.084)	0.669	-	-	-
35	Ovary	Ovary	100	0.895	−0.037 (0.152)	0.805	-	-	-
36	Pancreas	Pancreas	175	1.905	0.009 (0.116)	0.937	-	-	-
37	Pituitary	Pituitary	109	4.065	−0.059 (0.126)	0.635	-	-	-
38	Prostate	Prostate	105	3.961	−0.082 (0.137)	0.548	-	-	-
39	Skin	Not sun exposed (suprapubic) skin	239	2.698	0.116 (0.105)	0.269	-	-	-
40		Sun exposed (lower leg) skin	348	3.648	0.006 (0.078)	0.929	-	-	-
41	Small intestine	Terminal ileum	94	4.894	−0.172 (0.147)	0.245	-	-	-
42	Spleen	Spleen	110	0.937	0.153 (0.142)	0.282	-	-	-
43	Stomach	Stomach	189	11.447	−0.025 (0.143)	0.860	-	-	-
44	Testis	Testis	177	0.623	−0.137 (0.111)	0.220	-	-	-

#	Tissue type	Tissue subregion	*n*	sST2 (ENST00000311734.2)[a]			ST2L (ENST00000233954.1)[a]		
				Avg. transcript level[b]	*β* (SE)	*P*	Avg. transcript level[b]	*β* (SE)	*P*
45	Thyroid	Thyroid	322	2.764	−0.013 (0.079)	0.867	-	-	-
46	Uterus	Uterus	83	1.698	−0.081 (0.155)	0.617	-	-	-
47	Vagina	Vagina	90	2.105	−0.080 (0.146)	0.584	-	-	-
48	Whole blood	Whole blood	403	1.858	0.081 (0.076)	0.286	-	-	-

β, effect size; SE, standard error; sST2, soluble ST2; ST2L, full-length ST2. Bold and red text indicates statistical significance at a cutoff of $P < 0.05$.

[a] Only tissues in which >50% of individuals exhibited expression (reads per kilobase per million mapped reads [RPKM] > 0) were included to calculate the average expression of candidate genes and the association test between candidate gene levels and rs1921622 genotypes.

[b] Average expression of candidate genes among individuals in each tissue.

Supplementary Table 5. Demographic characteristics of the seven Alzheimer's disease datasets for meta-analysis.

**Chinese_cohort_2 (WGS)**	**Healthy control**	**Alzheimer's disease**
n	829	867
Age, years (SD)	69.14 (7.12)	71.15 (7.26)
Sex, male (%)	394 (47.53%)	391 (45.10%)
MMSE score (SD)	28.60 (1.41)	14.80 (6.27)
APOE-ε4, allele frequency	9.41%	26.07%
APOE-ε2, allele frequency	8.20%	4.84%
**Chinese_cohort_2 (Array)**	**Healthy control**	**Alzheimer's disease**
n	311	425
Age, years (SD)	69.82 (6.42)	71.17 (7.13)
Sex, male (%)	119 (38.26%)	169 (39.76%)
MMSE score (SD)	28.27 (1.77)	15.06 (6.08)
APOE-ε4, allele frequency	8.52%	32.35%
APOE-ε2, allele frequency	7.88%	5.29%
**LOAD**	**Healthy control**	**Alzheimer's disease**
n	2,231	464
Age, years (SD)	80.68 (10.75)	83.80 (6.62)
Sex, male (%)	871 (39.04%)	144 (31.03%)
APOE-ε4, allele frequency	20.77%	46.34%
APOE-ε2, allele frequency	6.90%	2.80%
**ADC1**	**Healthy control**	**Alzheimer's disease**
n	618	1,778
Age, years (SD)	75.73 (11.08)	79.39 (7.78)
Sex, male (%)	252 (40.78%)	847 (47.64%)
APOE-ε4, allele frequency	13.18%	40.07%
APOE-ε2, allele frequency	8.66%	2.92%
**ADC2**	**Healthy control**	**Alzheimer's disease**
n	308	772
Age, years (SD)	76.66 (7.03)	80.34 (6.68)
Sex, male (%)	90 (29.22%)	370 (47.93%)
APOE-ε4, allele frequency	15.26%	37.89%
APOE-ε2, allele frequency	9.74%	3.69%

**ADC3**	**Healthy control**	**Alzheimer's disease**
n	545	881
Age, years (SD)	75.72 (8.96)	79.98 (8.38)
Sex, male (%)	205 (37.61%)	402 (45.63%)
APOE-ε4, allele frequency	13.30%	37.97%
APOE-ε2, allele frequency	8.71%	3.86%
**ADNI**	**Healthy control**	**Alzheimer's disease**
n	290	378
Age, years (SD)	78.51 (6.64)	78.59 (6.95)
Sex, male (%)	147 (50.68%)	231 (61.11%)
APOE-ε4, allele frequency	13.45%	42.33%
APOE-ε2, allele frequency	7.24%	2.92%

ADC, Alzheimer's Disease Center dataset; ADNI, Alzheimer's Disease Neuroimaging Initiative dataset; LOAD, Late-Onset Alzheimer's Disease Study dataset; MMSE, Mini–Mental State Examination; SD, standard deviation; WGS, whole-genome sequencing.

Supplementary Table 6. Two-sample Mendelian randomization analysis for the causal effects of soluble ST2 on Alzheimer's disease in Chinese and European-descent populations.

sST2 level dataset	*n*	AD risk dataset	*n*	Instrumental variables	Sub-groups	*β* (SE)	*FDR*
**Chinese populations**							
Chinese_cohort_1 (WGS)	427	Chinese_cohort_2 (WGS)	1,696	rs1921622	Overall	−0.023 (0.066)	0.861
				rs55664618	Males	−0.027 (0.101)	0.861
				rs62151861	Females	−0.019 (0.083)	0.861
				rs2241116	Male *APOE*-ε4 carriers	−0.243 (0.115)	0.183
				rs1468790	Male *APOE*-ε4 noncarriers	0.179 (0.113)	0.199
				rs1523199	Female *APOE*-ε4 carriers	0.772 (0.163)	**1.70E−5**
				rs56238602	Female *APOE*-ε4 noncarriers	−0.200 (0.085)	0.065
**European-descent populations**							
**sST2 level dataset**	***n***	**AD risk dataset**	***n***	**Instrumental variables**	**Sub-groups**	***β* (SE)**	***FDR***
Sun et al., 2018 [11]	3,301	LOAD<br>ADC1<br>ADC2<br>ADC3<br>ADNI	8,265	rs1921622	Overall	0.077 (0.046)	0.131
				rs951774	Males	−0.007 (0.069)	0.919
				rs4851575	Females	0.140 (0.043)	**0.007**
				rs10515922	Male *APOE*-ε4 carriers	−0.176 (0.112)	0.138
				rs11123935	Male *APOE*-ε4 noncarriers	0.122 (0.072)	0.131
				rs10200945	Female *APOE*-ε4 carriers	0.168 (0.062)	**0.023**
				rs13001325	Female *APOE*-ε4 noncarriers	0.115 (0.063)	0.131

AD, Alzheimer's disease; ADC, Alzheimer's Disease Center; ADNI, Alzheimer's Disease Neuroimaging Initiative; LOAD, Late-Onset Alzheimer's Disease Study; sST2, soluble ST2; WGS, whole-genome sequencing; *β*, effect size; SE, standard error; *FDR*, false discovery rate. Bold and red text indicates statistical significance at a cutoff of *FDR* < 0.05.

Supplementary Table 7. Meta-analysis of the rs1921622 A allele on Alzheimer's disease risk in overall, male, and female participants in the discovery cohorts. Logistic regression test, adjusted for age, sex, and population structure, followed by meta-analysis and multiple testing correction.

	**Overall**						**Males**						**Females**					
	n	**HC**	**AD**	**β (SE)**	**OR**	***P***	***n***	**HC**	**AD**	**β (SE)**	**OR**	***P***	***n***	**HC**	**AD**	**β (SE)**	**OR**	***P***
**Chinese_cohort_1**	690	49.13%	51.01%	−0.167 (0.131)	0.84	0.20	250	42.50%	55.45%	0.221 (0.220)	1.24	0.32	440	53.66%	48.93%	−0.392 (0.166)	0.67	**0.01**
**Chinese_cohort_2 (WGS)**	1,696	42.58%	43.02%	0.015 (0.069)	1.01	0.82	785	43.27%	42.97%	−0.010 (0.100)	0.99	0.92	911	41.95%	43.06%	0.035 (0.095)	1.03	0.71
**Chinese_cohort_2 (Array)**	736	43.41%	42.94%	−0.019 (0.108)	0.98	0.86	288	40.76%	44.38%	0.165 (0.178)	1.18	0.35	448	45.05%	41.99%	−0.136 (0.137)	0.87	0.32
**LOAD**	2,695	52.84%	50.43%	−0.173 (0.073)	0.84	**0.01**	1,015	52.87%	51.74%	−0.130 (0.130)	0.88	0.32	1,680	52.83%	49.84%	−0.204 (0.089)	0.81	**0.02**
**ADC1**	2,396	52.92%	52.92%	−0.012 (0.066)	0.99	0.85	1,099	50.99%	53.13%	0.082 (0.104)	1.08	0.43	1,297	54.23%	52.73%	−0.077 (0.087)	0.92	0.37
**ADC2**	1,080	50.97%	54.27%	0.135 (0.098)	1.14	0.17	460	51.67%	56.08%	0.193 (0.171)	1.21	0.26	620	50.69%	52.61%	0.102 (0.120)	1.10	0.39
**ADC3**	1,426	55.87%	51.08%	−0.199 (0.079)	0.81	**0.01**	607	55.12%	50.99%	−0.188 (0.123)	0.83	0.13	819	56.32%	51.14%	−0.209 (0.104)	0.81	**0.04**
**ADNI**	668	53.45%	53.31%	−0.059 (0.119)	0.94	0.62	378	51.02%	55.19%	0.140 (0.166)	1.15	0.40	290	55.94%	50.34%	−0.310 (0.178)	0.73	0.08
**Meta-analysis in Chinese**	OR = 0.977; RE2 *P* = 0.721; *FDR* = 0.827						OR = 1.059; RE2 *P* = 0.532; *FDR* = 0.745						OR = 0.873; RE2 *P* = 0.196; *FDR* = 0.513					
**Meta-analysis in European**	OR = 0.933; RE2 *P* = **0.024**; *FDR* = 0.057						OR = 1.001; RE2 *P* = 0.852; *FDR* = 0.852						OR = 0.879; RE2 *P* = **0.009**; *FDR* = **0.049**					
**Overall meta-analysis**	OR = 0.942; RE2 *P* = **0.044**; *FDR* = 0.102						OR = 1.020; RE2 *P* = 0.773; *FDR* = 0.773						OR = 0.883; RE2 *P* = **0.004**; *FDR* = **0.014**					

AD, Alzheimer's disease; ADC, Alzheimer's Disease Center cohort; ADNI, Alzheimer's Disease Neuroimaging Initiative cohort; HC, healthy control; LOAD, Late-Onset Alzheimer's Disease Study cohort; WGS, whole-genome sequencing; β, effect size; SE, standard error; OR, odds ratio; RE2 *P*, Han and Eskin's random effects model-based meta *P*-value; *FDR*, false discovery rate . Bold and red text indicates statistical significance at a cutoff of $P < 0.05$ or $FDR < 0.05$.

Supplementary Table 8. Meta-analysis of the rs1921622 A allele on Alzheimer's disease risk in male and female *APOE*-ε4 carriers and noncarriers in the discovery cohorts. Logistic regression test, adjusted for age and population structure, followed by meta-analysis and multiple testing correction.

	Male *APOE*-ε4 carriers						Female *APOE*-ε4 carriers					
	n	**HC**	**AD**	***β*** **(SE)**	**OR**	***P***	***n***	**HC**	**AD**	***β*** **(SE)**	**OR**	***P***
**Chinese_cohort_1**	62	54.00%	60.81%	−0.861 (0.594)	0.42	0.14	109	60.00%	45.83%	−0.782 (0.358)	0.45	**0.02**
**Chinese_cohort_2 (WGS)**	225	35.91%	45.13%	0.371 (0.206)	1.44	0.07	292	52.74%	41.09%	−0.451 (0.188)	0.63	**0.01**
**Chinese_cohort_2 (Array)**	91	46.15%	44.87%	0.025 (0.457)	1.02	0.95	182	55.13%	39.51%	−0.673 (0.261)	0.51	**0.01**
**LOAD**	456	53.35%	53.98%	−0.053 (0.160)	0.95	0.74	738	55.75%	51.49%	−0.248 (0.119)	0.78	**0.03**
**ADC1**	633	52.34%	53.34%	0.045 (0.190)	1.05	0.81	670	52.17%	51.21%	−0.069 (0.160)	0.93	0.66
**ADC2**	243	43.75%	59.36%	0.646 (0.326)	1.90	**0.04**	316	57.94%	53.55%	−0.184 (0.202)	0.83	0.36
**ADC3**	309	59.00%	51.93%	−0.297 (0.221)	0.74	0.17	373	54.17%	51.04%	−0.122 (0.181)	0.88	0.50
**ADNI**	194	48.72%	56.77%	0.332 (0.279)	1.39	0.23	130	57.81%	50.00%	−0.376 (0.296)	0.68	0.20
**Meta-analysis in Chinese**	OR = 1.021; RE2 *P* = 0.293; *FDR* = 0.513						OR = 0.567; RE2 *P* = **7.60E−5**; *FDR* = **5.32E−4**					
**Meta-analysis in European**	OR = 1.068; RE2 *P* = 0.797; *FDR* = 0.852						OR = 0.830; RE2 *P* = **0.014**; *FDR* = **0.049**					
**Overall meta-analysis**	OR = 1.086; RE2 *P* = 0.412; *FDR* = 0.577						OR = 0.757; RE2 *P* = **7.78E−5**; *FDR* = **5.44E−4**					
	**Male *APOE*-ε4 noncarriers**						**Female *APOE*-ε4 noncarriers**					
	n	**HC**	**AD**	***β*** **(SE)**	**OR**	***P***	***n***	**HC**	**AD**	***β*** **(SE)**	**OR**	***P***
**Chinese_cohort_1**	188	40.00%	52.74%	0.359 (0.246)	1.43	0.15	331	52.78%	50.66%	−0.208 (0.203)	0.81	0.31
**Chinese_cohort_2 (WGS)**	560	44.89%	41.56%	−0.131 (0.121)	0.88	0.28	619	39.78%	44.74%	0.202 (0.120)	1.22	0.09
**Chinese_cohort_2 (Array)**	197	40.09%	43.96%	0.174 (0.213)	1.19	0.42	266	42.48%	45.13%	0.128 (0.184)	1.13	0.48
**LOAD**	559	52.56%	43.55%	−0.429 (0.272)	0.65	0.12	942	51.11%	45.35%	−0.353 (0.165)	0.70	**0.03**
**ADC1**	466	50.53%	52.69%	0.091 (0.137)	1.09	0.51	627	54.93%	55.24%	−0.006 (0.113)	0.99	0.95
**ADC2**	217	54.54%	51.32%	−0.140 (0.219)	0.87	0.52	304	47.74%	51.00%	0.219 (0.173)	1.24	0.21
**ADC3**	298	53.87%	49.30%	−0.204 (0.170)	0.82	0.23	446	57.03%	51.31%	−0.233 (0.142)	0.79	0.10
**ADNI**	184	51.85%	51.97%	−0.022 (0.243)	0.98	0.93	160	55.41%	51.02%	−0.221 (0.275)	0.80	0.42
**Meta-analysis in Chinese**	OR = 1.079; RE2 *P* = 0.827; *FDR* = 0.827						OR = 1.084; RE2 *P* = 0.290; *FDR* = 0.513					
**Meta-analysis in European**	OR = 0.921; RE2 *P* = 0.403; *FDR* = 0.565						OR = 0.898; RE2 *P* = 0.154; *FDR* = 0.271					
**Overall meta-analysis**	OR = 0.960; RE2 *P* = 0.586; *FDR* = 0.684						OR = 0.961; RE2 *P* = 0.233; *FDR* = 0.408					

AD, Alzheimer's disease; ADC, Alzheimer's Disease Center cohort; ADNI, Alzheimer's Disease Neuroimaging Initiative cohort; HC, healthy control; LOAD, Late-Onset Alzheimer's Disease cohort; WGS, whole-genome sequencing; *β*, effect size; SE, standard error; OR, odds ratio; RE2 *P*, Han and Eskin's random effects model-based meta *P*-value; *FDR*, false discovery rate. Bold and red text indicates statistical significance at a cutoff of $P < 0.05$ or $FDR < 0.05$.

Supplementary Table 9. Associations between the rs1921622 A allele and Alzheimer's disease-related endophenotypes in patients with Alzheimer's disease in the discovery cohorts. Linear regression test, adjusted for covariates such as age, sex, and population structure (see ***Methods*** for details), followed by multiple testing correction.

	**Onset age of dementia**			**MMSE scores**		**Volumes of the entorhinal cortex**		**Plasma P-tau181 levels**		**Plasma NfL levels**	
Datasets	LOAD, ADC1–3			Chinese_cohort_2		ADNI		Chinese_cohort_1		Chinese_cohort_1	
n	3,348			1,292		847		156		154	
Sub-groups	***β*** **(SE)**	**HR**	***FDR***	***β*** **(SE)**	***FDR***	***β*** **(SE)**	***FDR***	***β*** **(SE)**	***FDR***	***β*** **(SE)**	***FDR***
Overall	−0.035 (0.035)	0.965	0.293	0.596 (0.247)	**0.038**	0.034 (0.045)	0.624	−0.085 (0.800)	0.915	−0.435 (0.922)	0.886
Males	0.054 (0.053)	1.055	0.283	−0.089 (0.373)	0.811	−0.033 (0.059)	0.665	0.398 (0.381)	0.527	−0.257 (1.794)	0.886
Females	−0.090 (0.041)	0.913	**0.047**	1.130 (0.330)	**0.002**	0.165 (0.057)	**0.027**	−0.432 (0.330)	0.455	−0.438 (1.088)	0.886
Male *APOE*-ε4 carriers	0.078 (0.045)	1.082	0.241	−0.587 (0.612)	0.475	−0.075 (0.085)	0.624	0.461 (0.246)	0.280	−2.630 (2.975)	0.886
Male *APOE*-ε4 noncarriers	0.020 (0.152)	1.020	0.283	0.260 (0.467)	0.675	0.012 (0.086)	0.891	0.379 (0.474)	0.601	0.907 (2.078)	0.886
Female *APOE*-ε4 carriers	−0.134 (0.041)	0.874	**0.011**	1.622 (0.440)	**0.001**	0.214 (0.079)	**0.027**	−0.676 (0.334)	0.280	−2.970 (1.450)	0.327
Female *APOE*-ε4 noncarriers	−0.064 (0.058)	0.938	0.330	0.837 (0.492)	0.158	0.160 (0.121)	0.446	−0.082 (0.472)	0.915	0.358 (1.303)	0.886

AD, Alzheimer's disease; ADC, Alzheimer's Disease Center; ADNI, Alzheimer's Disease Neuroimaging Initiative; LOAD, Late-Onset Alzheimer's Disease Study; MMSE, Mini-Mental State Examination; NfL, neurofilament light polypeptide; *β*, effect size; SE, standard error; HR, hazard ratio; *FDR*, false discovery rate. Bold and red text indicates statistical significance at a cutoff of *FDR* < 0.05.

Uncropped gel images for Supplementary Fig. 4b. L, DNA ladder; Ctrl, isogenic control clones; Δ38bp, 38-bp deletion clones; Δ67bp, 67-bp deletion clones.

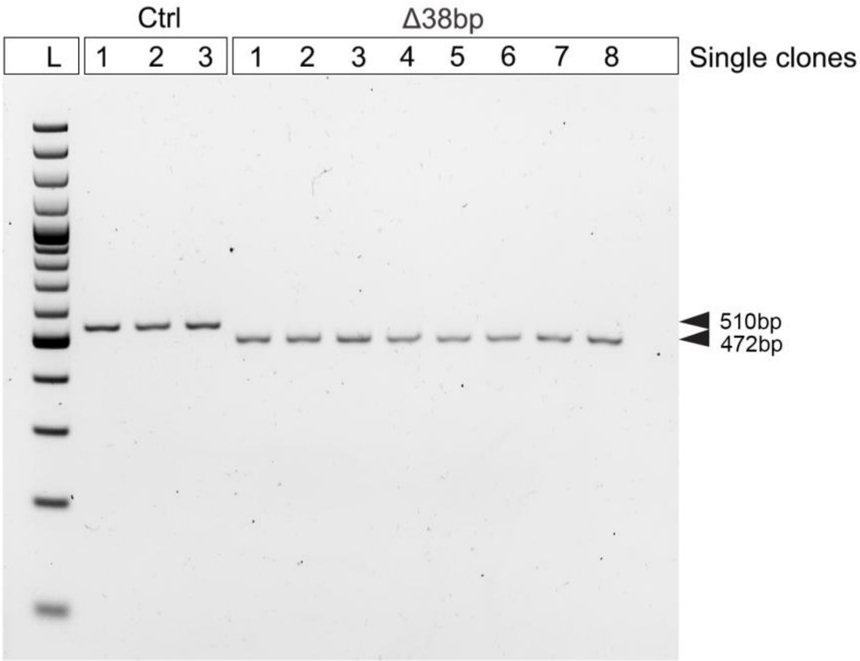

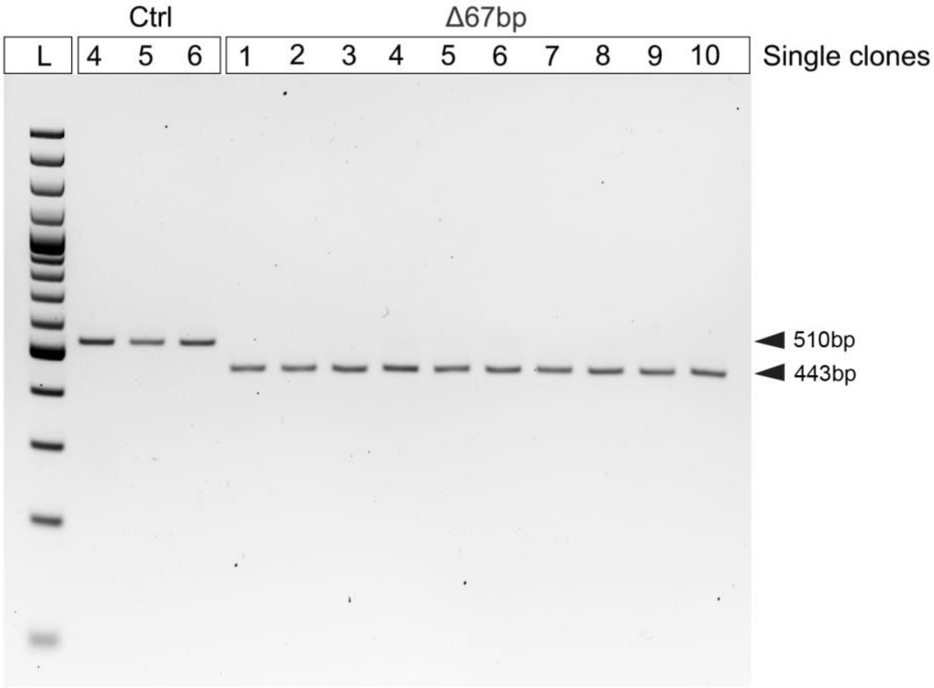

References

1. Lee, J.H., Cheng, R., Graff-Radford, N., Foroud, T. & Mayeux, R. Analyses of the national institute on aging late-onset alzheimer's disease family study: implication of additional loci. *Archives of neurology* **65**, 1518-1526 (2008).
2. Naj, A.C., *et al.* Common variants at MS4A4/MS4A6E, CD2AP, CD33 and EPHA1 are associated with late-onset Alzheimer's disease. *Nature genetics* **43**, 436-441 (2011).
3. Jun, G., *et al.* Meta-analysis confirms CR1, CLU, and PICALM as alzheimer disease risk loci and reveals interactions with APOE genotypes. *Archives of neurology* **67**, 1473-1484 (2010).
4. Kathryn, A.E., *et al.* The Australian Imaging, Biomarkers and Lifestyle (AIBL) study of aging: methodology and baseline characteristics of 1112 individuals recruited for a longitudinal study of Alzheimer's disease. *International Psychogeriatrics* (2009).
5. Rowe, C.C., *et al.* Amyloid imaging results from the Australian Imaging, Biomarkers and Lifestyle (AIBL) study of aging. *Neurobiology of aging* **31**, 1275-1283 (2010).
6. Bourgeat, P., *et al.* Comparison of MR-less PiB SUVR quantification methods. *Neurobiology of aging* **36**, S159-S166 (2015).
7. Clark, C.M., *et al.* Use of florbetapir-PET for imaging β-amyloid pathology. *Jama* **305**, 275-283 (2011).
8. Lundqvist, R., *et al.* Implementation and validation of an adaptive template registration method for 18F-flutemetamol imaging data. *Journal of Nuclear Medicine* **54**, 1472-1478 (2013).
9. Consortium, G. The Genotype-Tissue Expression (GTEx) pilot analysis: Multitissue gene regulation in humans. *Science* **348**, 648-660 (2015).
10. Lonsdale, J., *et al.* The genotype-tissue expression (GTEx) project. *Nature genetics* **45**, 580-585 (2013).
11. Sun, B.B., *et al.* Genomic atlas of the human plasma proteome. *Nature* **558**, 73-79 (2018).
