# Supplementary material for: An IL1RL1 genetic variant lowers soluble ST2 levels and the risk effects of APOE-ε4 in female patients with Alzheimer’s disease
Source: Nat Aging. 2022 Jul 15;2(7):616–34. doi: 10.1038/s43587-022-00241-9 (PMC10154240; doi:10.1038/s43587-022-00241-9)
Supplement: Supplementary file 2 — Reporting Summary [file 43587_2022_241_MOESM2_ESM.pdf]

Corresponding author(s): Nancy Y. Ip
Last updated by author(s): Apr 28, 2022

Reporting Summary

Nature Portfolio wishes to improve the reproducibility of the work that we publish. This form provides structure for consistency and transparency in reporting. For further information on Nature Portfolio policies, see our Editorial Policies and the Editorial Policy Checklist.

Statistics

For all statistical analyses, confirm that the following items are present in the figure legend, table legend, main text, or Methods section.

n/a	Confirmed	
☐	☒	The exact sample size (*n*) for each experimental group/condition, given as a discrete number and unit of measurement
☐	☒	A statement on whether measurements were taken from distinct samples or whether the same sample was measured repeatedly
☐	☒	The statistical test(s) used AND whether they are one- or two-sided<br>*Only common tests should be described solely by name; describe more complex techniques in the Methods section.*
☐	☒	A description of all covariates tested
☐	☒	A description of any assumptions or corrections, such as tests of normality and adjustment for multiple comparisons
☐	☒	A full description of the statistical parameters including central tendency (e.g. means) or other basic estimates (e.g. regression coefficient) AND variation (e.g. standard deviation) or associated estimates of uncertainty (e.g. confidence intervals)
☐	☒	For null hypothesis testing, the test statistic (e.g. *F*, *t*, *r*) with confidence intervals, effect sizes, degrees of freedom and *P* value noted<br>*Give P values as exact values whenever suitable.*
☒	☐	For Bayesian analysis, information on the choice of priors and Markov chain Monte Carlo settings
☒	☐	For hierarchical and complex designs, identification of the appropriate level for tests and full reporting of outcomes
☐	☒	Estimates of effect sizes (e.g. Cohen's *d*, Pearson's *r*), indicating how they were calculated

Our web collection on statistics for biologists contains articles on many of the points above.

Software and code

Policy information about availability of computer code

Data collection	We worked with the commercial software integrated in Simoa HD-X, VersaMax microplate reader, illumina HiSeq X, QuantStudio 7 Flex Real-Time PCR system, BD Influx cell sorter flow cytometer, MRI imaging, and Amyloid-PET technologies<br>Image Acquisition: ZEN microscope software (v3.2 and v3.3)
Data analysis	SNP array genotyping: TaqMan genotyper software (Applied Biosystems)<br>Imaging analysis: GraphPad Prism (v8.0), Fiji-ImageJ (v1.53c), Imaris (v9.7.2)<br>Flow Cytometry analysis: FlowJo software (v10.5.0)<br>SnRNAseq analysis: Cell Ranger (v3.0.1), Seurat (v3.0)<br>Haplotype and fine-mapping analysis: Haploview (v4.2), CAVIAR software (v2.2)<br>Association analysis: PLINK (v1.9), METASOFT (v2.0.0), R (v3.6.2) with packages installed including plotly (v4.9.1), relaimpo (v2.2-3), GenABEL (v1.8), TwoSampleMR (v0.5.6), ForestPMPlot (v1.0.2), OptimalCutpoints (v1.1-4), survival (v1.3-24), ggplot2 (v3.2.1), qqman (v0.1.4).<br>Additional details on parameters can be found in the Methods section.<br>The custom codes for statistical analyses and data visualization are available on GitHub (https://github.com/yjiangah/sST2-in-AD)

For manuscripts utilizing custom algorithms or software that are central to the research but not yet described in published literature, software must be made available to editors and reviewers. We strongly encourage code deposition in a community repository (e.g. GitHub). See the Nature Portfolio guidelines for submitting code & software for further information.

Data

Policy information about availability of data

All manuscripts must include a data availability statement. This statement should provide the following information, where applicable:

- Accession codes, unique identifiers, or web links for publicly available datasets
- A description of any restrictions on data availability
- For clinical datasets or third party data, please ensure that the statement adheres to our policy

All statistical data associated with this study are contained in the Main Text, Supplementary Information, Supplementary Data Files, or Source Data Files. The consent forms signed by individual participants from the Chinese_cohort_1 state that the research content will be kept private under the supervision of the hospital and research team. Therefore, the phenotypic, genomic, and proteomic data of individual participants will only be available and shared in formal collaborations. A review panel hosted at HKUST will process and review any applications for data sharing and project collaboration and promptly notify applicants with the decision. Researchers may contact [sklneurosci@ust.hk] for details about data sharing and project collaboration related to the present study. The GRCh38/hg38 reference genome is available at https://hgdownload.soe.ucsc.edu/downloads.html. The human frontal cortex snRNA-seq dataset of the UKBBN cohort has been deposited in GEO (accession no. GSE157827). The genomic, demographic, and clinical data of the LOAD cohort are available on the National Institutes of Health (NIH) database of Genotypes and Phenotypes (dbGaP) project (accession number: phs000168.v2.p2). The genomic, demographic, and clinical data of the ADC1–3 cohorts are available on the NIH dbGaP project (accession number: phs000372.v2.p1). The genomic, demographic, clinical and brain imaging data of the ADNI cohort are available at adni.loni.usc.edu upon request. The proteomic and demographic data from the INTERVAL and LonGenity cohorts are available at https://twc-stanford.shinyapps.io/aging_plasma_proteome/. The genomic, demographic, and transcriptomic data from the GTEx cohort are available on the NIH dbGaP project (accession number: phs000424.v6.p1). The genomic, demographic, clinical and brain imaging data of the AIBL cohort are available at www.aibl.csiro.au upon request. All other data are available from the corresponding authors upon reasonable request.

Field-specific reporting

Please select the one below that is the best fit for your research. If you are not sure, read the appropriate sections before making your selection.

☒ Life sciences ☐ Behavioural & social sciences ☐ Ecological, evolutionary & environmental sciences

For a reference copy of the document with all sections, see nature.com/documents/nr-reporting-summary-flat.pdf

Life sciences study design

All studies must disclose on these points even when the disclosure is negative.

Sample size	No statistical methods were used to predetermine sample size. For human subjects, we took all available genomic, proteomic/transcriptomic, brain imaging and phenotypic data from the Chinese_cohort_1, Chinese_cohort_2, LOAD cohort, ADC cohorts, ADNI cohort, UKBBN cohort, AIBL cohort, GTEx cohort, ADRC cohort and Japanese cohort. Sample sizes are either listed in Supplementary Table 1, Supplementary Table 5, Supplementary Data, or in corresponding main text and figure legends. For mouse model studies, sample size was determined based on the number of animals used in prior experiments conducted (Fu et al., PNAS 2016; Lau et al., Cell Reports 2020.)
Data exclusions	No data was excluded.
Replication	All data presented were obtained from two to three independent experiments with consistent outcomes.
Randomization	All human samples in different disease conditions (e.g., healthy controls, patients with AD etc.) were collected and randomly assigned with a code, with a corresponding decoding file. All non-human samples (e.g,. mice, cultured cells) were randomly assigned into experimental groups and conditions before the experiments and sample collection.<br>The operator was blinded towards the code and decoding file.
Blinding	All samples had a random code and the operator was blinded towards the code.

Reporting for specific materials, systems and methods

We require information from authors about some types of materials, experimental systems and methods used in many studies. Here, indicate whether each material, system or method listed is relevant to your study. If you are not sure if a list item applies to your research, read the appropriate section before selecting a response.

Materials & experimental systems

n/a	Involved in the study
☐	☒ Antibodies
☐	☒ Eukaryotic cell lines
☒	☐ Palaeontology and archaeology
☐	☒ Animals and other organisms
☐	☒ Human research participants
☒	☐ Clinical data
☒	☐ Dual use research of concern

Methods

n/a	Involved in the study
☒	☐ ChIP-seq
☐	☒ Flow cytometry
☐	☒ MRI-based neuroimaging

Antibodies

Antibodies used

Primary Antibodies:
Mouse anti-Aβ monoclonal antibody (clone NAB228; SC-32277; Santa Cruz Biotechnology)
Mouse anti-Aβ monoclonal antibody (clone 4G8; 800703; BioLegend)
Rabbit anti-Aβ monoclonal antibody (clone D54D2; 8243S; Cell Signaling Technology)
Rabbit anti-Iba-1 polyclonal antibody (019-19741; FUJIFILM Wako Pure Chemical Corporation)
Rat anti-Ki67 monoclonal antibody (clone SolA15; 14-5698-80; eBioscience)
Alexa Fluor 488-conjugated mouse CD11b antibody (53-0112-82; eBioscience)
Rabbit Histone H3K4me3 polyclonal antibody (39159; Active Motif)
Rabbit Histone H3K27ac polyclonal antibody (39133; Active Motif)
Human IgG, Fc fragment (009-000-008; Jackson ImmunoResearch)
Normal Rabbit IgG Control (AB-105-C; R&D Systems)

Secondary Antibodies:
HRP-labelled anti-mouse IgG (QD440-XAKE; BioGenex)
HRP-labelled anti-mouse Ig and AP-labelled anti-rabbit Ig detection cocktail (HK597-50K; BioGenex)
Biotin-conjugated anti-mouse secondary antibody (BA2000; Vector Laboratories)
Goat anti-mouse IgG (H+L) Alexa Fluor488-conjugate (A-11001; Invitrogen Life Technologies)
Goat anti-rabbit IgG (H+L) Alexa Fluor568-conjugate (A-11011; Invitrogen Life Technologies)
Goat anti-rat IgG (H+L) Alexa Fluor647-conjugate (A-21247; Invitrogen Life Technologies)

Validation

All antibodies used were validated by manufacturers. Data sheet is available from the web links as described below.
Mouse anti-Aβ monoclonal antibody (clone NAB228; SC-32277; Santa Cruz Biotechnology): https://datasheets.scbt.com/sc-32277.pdf
Mouse anti-Aβ monoclonal antibody (clone 4G8; 800703; BioLegend): https://www.biolegend.com/en-us/global-elements/pdf-popup/purified-anti-beta-amyloid-17-24-antibody-11233?filename=Purified%20anti-beta-Amyloid%2017-24%20%20Antibody.pdf&pdfgen=true
Rabbit anti-Aβ monoclonal antibody (clone D54D2; 8243S; Cell Signaling Technology): https://www.cellsignal.com/products/primary-antibodies/b-amyloid-d54d2-xp-rabbit-mab/8243
Rabbit anti-Iba-1 polyclonal antibody (019-19741; FUJIFILM Wako Pure Chemical Corporation): https://labchem-wako.fujifilm.com/us/product/detail/W01W0101-1974.html
Rat anti-Ki67 monoclonal antibody (clone SolA15; 14-5698-80; eBioscience): https://www.thermofisher.com/order/genome-database/generatePdf?productName=Ki-67&assayType=PRANT&detailed=true&productId=14-5698-82
Alexa Fluor 488-conjugated mouse CD11b antibody (53-0112-82; eBioscience): https://www.thermofisher.com/order/genome-database/generatePdf?productName=CD11b&assayType=PRANT&detailed=true&productId=53-0112-82
Rabbit Histone H3K4me3 polyclonal antibody (39159; Active Motif): https://www.activemotif.com/documents/tds/39159.pdf
Rabbit Histone H3K27ac polyclonal antibody (39133; Active Motif): https://www.activemotif.com/documents/tds/39133.pdf
Human IgG, Fc fragment (009-000-008; Jackson ImmunoResearch): https://www.jacksonimmuno.com/lots/000000142895
Normal Rabbit IgG Control (AB-105-C; R&D Systems): https://resources.rndsystems.com/pdfs/datasheets/ab105c.pdf?v=20220413&_ga=2.85229085.521716042.1649913381-812846025.1649913381

Eukaryotic cell lines

Policy information about cell lines

Cell line source(s)	The human cerebral microvascular endothelial cell line (hCMEC/D3) (Cedarlane)
Authentication	Immunofluorescence staining of hCMEC/D3 for endothelial cell specific markers, specifically Tight junction protein ZO-1 and VECadherin was used to validate the cell line.
Mycoplasma contamination	The hCMEC/D3 cells were tested negative for mycoplasma contamination.
Commonly misidentified lines (See ICLAC register)	No commonly misidentified cell lines were used.

Animals and other organisms

Policy information about studies involving animals; ARRIVE guidelines recommended for reporting animal research

Laboratory animals	C57BL6J male and female mice (3 months of age) were purchased from the Jackson Laboratory.<br>5XFAD male and female mice (B6.Cg-Tg(APPSwFlLon,PSEN1*M146L*L286V)6799Vas/Mmjax) (3 months of age) were provided by Sookja Kim Chung (The University of Hong Kong).<br>All mice were housed in the HKUST Animal and Plant Care Facility. We housed 4 mice of the same sex per cage at 22 degrees Celsius and at a relative humidity of 60%, with a 12-h light/dark cycle as well as food and water ad libitum
Wild animals	The study did not involve wild animals.
Field-collected samples	The study did not involve samples collected from the field.
Ethics oversight	All animal experiments were approved by the HKUST Animal Ethics Committee and conducted in accordance with the Guidelines of the Animal Care Facility of HKUST.

Note that full information on the approval of the study protocol must also be provided in the manuscript.

Human research participants

Policy information about studies involving human research participants

Population characteristics	The Chinese_cohort_1 consisted of 345 patients with AD and 345 healthy controls (all ≥60 years old). All participants underwent a medical history assessment, clinical assessment, cognitive and functional assessments using the Montreal Cognitive Assessment (MoCA) test, and neuroimaging assessment by magnetic resonance imaging (MRI), and only those with cognitive dysfunctions (i.e., those having Alzheimer's dementia with MoCA score < 21) were included in the AD group in this study. Participants with any significant neurological disease besides AD or psychiatric disorder were excluded. We recorded age, sex, years of education, medical history, and history of CVDs (i.e., heart disease, hypertension, diabetes mellitus, and hyperlipidemia).The detailed characteristics of the cohort including age, gender, genotypic information and phenotypic information are listed in Supplementary Table 1. The characteristics of other datasets are listed in Supplementary Table 5, Supplementary Data, or in corresponding main text and figure legends.
Recruitment	The Chinese_cohort_1 consisted of 690 Hong Kong Chinese participants who visited the Specialist Outpatient Department of the Prince of Wales Hospital of the Chinese University of Hong Kong from April 2013 to February 2018 . All participants underwent medical history assessment, clinical assessment, cognitive and functional assessment using the Montreal Cognitive Assessment (MoCA) test and neuroimaging assessment by MRI. The clinical diagnosis of AD was established on the basis of the American Psychiatric Association's Diagnostic and Statistical Manual of Mental Disorders, Fifth Edition (DSM-5). Participants with any significant neurologic disease besides AD or psychiatric disorder were excluded. All participants provided written informed consent for both study enrollment and sample collection. The recruitment of other cohorts are described in Supplementary Notes section. No selection biases were identified.
Ethics oversight	The study of Chinese_cohort_1 was approved by the Joint Chinese University of Hong Kong-New Territories East Cluster Clinical Research Ethics Committee at the Prince of Wales Hospital, the Chinese University of Hong Kong, and the Hong Kong University of Science and Technology. All participants provided written informed consent for both study enrollment and sample collection.<br>The samples of UKBBN cohort are provided by South West Dementia Brain Bank (SWDBB), which gets approval from North Somerset and South Bristol Research Ethics Committee to operate as a research tissue bank. The SWDBB is part of the Brains for Dementia Research program, jointly funded by Alzheimer's Research UK and Alzheimer's Society and supported by BRACE (Bristol Research into Alzheimer's and Care of the Elderly) and the Medical Research Council.

Note that full information on the approval of the study protocol must also be provided in the manuscript.

Flow Cytometry

Plots

Confirm that:

☒ The axis labels state the marker and fluorochrome used (e.g. CD4-FITC).

☒ The axis scales are clearly visible. Include numbers along axes only for bottom left plot of group (a 'group' is an analysis of identical markers).

☒ All plots are contour plots with outliers or pseudocolor plots.

☒ A numerical value for number of cells or percentage (with statistics) is provided.

Methodology

Sample preparation	5XFAD or C57BL6J (4 months of age) mice were intraperitoneally injected with methoxy-X04 (10 mg/kg) to label Aβ. The mice were anesthetized with isoflurane 3 h after methoxy-X04 injection, and the left ventricle was perfused with ice-cold PBS. Their forebrains were isolated, minced, and incubated at 37 °C for 30 min in 5 U/mL papain (LS003126) and 35 U/mL DNase I (LS002140; Worthington Biochemical) for enzymatic digestion. After incubation, myelin debris was depleted by 30% isotonic Percoll (P1644; Sigma-Aldrich) gradient centrifugation, and mononuclear cell suspensions were obtained in DMEM/F12

	medium with ice-cold 10% heat-inactivated FBS. Unstained controls were prepared from mixtures of different sample cell suspensions for cell population identification. To label microglia, an Alexa Fluor 488-conjugated mouse CD11b antibody (1:200; 53-0112-82; eBioscience) was used to stain the cell suspensions for 45 min at 4 °C.
Instrument	BD Influx cell sorter
Software	FlowJo software (v10.5.0)
Cell population abundance	Post-sort fractions for methoxy-X04+ CD11b+ cells were >95%
Gating strategy	Cells were gated on forward (FSC = size) and sideward scatter (SSC = internal structure). FSC and trigger pulse width were used to discriminate single cells from cell doublets/aggregates. Unstained controls were used to identify CD11b+ cell populations. Samples from C57BL6J mice were used to identify methoxy-X04+ cell populations.

☒ Tick this box to confirm that a figure exemplifying the gating strategy is provided in the Supplementary Information.

Magnetic resonance imaging

Experimental design

Design type	Cross-sectional morphometry analysis (structural MRI only)
Design specifications	One T1-weighted structural MRI was conducted per participant for relationships with sST2 protein levels and genotypes
Behavioral performance measures	None

Acquisition

Imaging type(s)	Structural MRI
Field strength	3T
Sequence & imaging parameters	In Chinese_cohort_1, 3D FFE (gradient echo) pulse sequence was used. The images were acquired from coronal view, with slice thickness 5mm, TE/TR/flip angle = 3ms /7ms /8. The acquired image matrix size was 240 × 25 × 240. In ADNI, structural MRI was recorded using a 3D T1 weighted MPRAGE sequence with 1mm isotropic voxel-size and a TR=2300ms
Area of acquisition	Whole brain

Diffusion MRI ☐ Used ☒ Not used

Preprocessing

Preprocessing software	In Chinese_cohort_1, the MRI data were preprocessed by AccuBrain® IV1.2 (BrainNow Medical Technology Ltd), a brain quantification tool that performs brain structure and tissue segmentation and quantification in a fully automatic mode. Given the T1-weighted MRI data, several brain structures (e.g., hippocampus, lateral ventricle, amygdala, etc) and three major brain tissues (i.e., white matter, gray matter and CSF) are segmented automatically based on prior anatomical knowledge specified by experienced radiologists. The anatomical information is automatically transformed into the individual brain. In ADNI, FSL, ANTs, AFNI were used
Normalization	In Chinese_cohort_1, for details please refer to Abrigo et al., Acta Radiologica 2018. In ADNI, non-linear spatial normalization parameters were estimated based on structural T1-weighted images using Advanced Normalization Tools (ANTs), to normalize all images to Montreal Neurological Institute (MNI) standard space.
Normalization template	In Chinese_cohort_1, for details please refer to Abrigo et al., Acta Radiologica 2018. In ADNI, MNI was used.
Noise and artifact removal	For details please refer to Abrigo et al., Acta Radiologica 2018
Volume censoring	Not applicable to structural MRI

Statistical modeling & inference

Model type and settings	Only structural MRI was used in analyses with ROI-based approach. Additional details on statistical methods, including all model parameters can be found in the Methods section.
Effect(s) tested	We tested for associations between imaging measures (gray matter volume) and sST2 levels or genotypes

Specify type of analysis: ☐ Whole brain ☒ ROI-based ☐ Both

Anatomical location(s): Automated labelling by Accubrain

Statistic type for inference (See Eklund et al. 2016) | Neither voxel-wise or cluster-wise was used

Correction | FDR

Models & analysis

n/a	Involved in the study
☒	☐ Functional and/or effective connectivity
☒	☐ Graph analysis
☒	☐ Multivariate modeling or predictive analysis
